# *Haloquadratum walsbyi* : Limited Diversity in a Global Pond

**DOI:** 10.1371/journal.pone.0020968

**Published:** 2011-06-20

**Authors:** Mike L. Dyall-Smith, Friedhelm Pfeiffer, Kathrin Klee, Peter Palm, Karin Gross, Stephan C. Schuster, Markus Rampp, Dieter Oesterhelt

**Affiliations:** 1 Department of Membrane Biochemistry, Max-Planck-Institute of Biochemistry, Martinsried, Germany; 2 Computing Center (RZG) of the Max-Planck-Society, Max Planck Institute of Plasma Physics, Garching, Germany; 3 Center for Comparative Genomics and Bioinformatics, Penn State University, University Park, Pennsylvania, United States of America; Université Paris Sud, France

## Abstract

**Background:**

*Haloquadratum walsbyi* commonly dominates the microbial flora of hypersaline waters. Its cells are extremely fragile squares requiring >14%(w/v) salt for growth, properties that should limit its dispersal and promote geographical isolation and divergence. To assess this, the genome sequences of two isolates recovered from sites at near maximum distance on Earth, were compared.

**Principal Findings:**

Both chromosomes are 3.1 MB in size, and 84% of each sequence was highly similar to the other (98.6% identity), comprising the core sequence. ORFs of this shared sequence were completely synteneic (conserved in genomic orientation and order), without inversion or rearrangement. Strain-specific insertions/deletions could be precisely mapped, often allowing the genetic events to be inferred. Many inferred deletions were associated with short direct repeats (4–20 bp). Deletion-coupled insertions are frequent, producing different sequences at identical positions. In cases where the inserted and deleted sequences are homologous, this leads to variant genes in a common synteneic background (as already described by others). Cas/CRISPR systems are present in C23^T^ but have been lost in HBSQ001 except for a few spacer remnants. Numerous types of mobile genetic elements occur in both strains, most of which appear to be active, and with some specifically targetting others. Strain C23^T^ carries two ∼6 kb plasmids that show similarity to halovirus His1 and to sequences nearby halovirus/plasmid gene clusters commonly found in haloarchaea.

**Conclusions:**

Deletion-coupled insertions show that *Hqr. walsbyi* evolves by uptake and precise integration of foreign DNA, probably originating from close relatives. Change is also driven by mobile genetic elements but these do not by themselves explain the atypically low gene coding density found in this species. The remarkable genome conservation despite the presence of active systems for genome rearrangement implies both an efficient global dispersal system, and a high selective fitness for this species.

## Introduction

First described in 1980 [1], the square haloarchaeon, *Haloquadratum walsbyi,* is commonly the dominant species found in hypersaline waters such as salt lakes and saltern crystallizer ponds [2], [3]. Its cells are highly distinctive, being thin squares or rectangles, usually containing gas vesicles and polyhydroxybutyrate (PHA) granules [4], [5]. It thrives at saturating salt concentrations, where it can represent ≥80% of the microbial population [6], and its cytoplasm is completely adapted to function optimally at similarly high levels of potassium chloride. Cell growth requires salt concentrations of at least 14% w/v, or more than 4-fold higher than seawater, and it can also tolerate molar concentrations of Mg^2+^ making it one of a limited number of organisms able to cope at such extremely low water activity [7]. In fact, it achieves higher cell densities in media with >1 M MgCl_2_ [8]. The genome has a G+C content of 48%, considerably lower than all the other known species of the family *Halobacteriaceae*, which have values of 61–70% [9].

It took until 2004 for these organisms to be cultivated in the laboratory [10], [11], and two isolates were formally described as a new species in 2007 [8]. They were recovered from saltern crystallizer ponds, one in Australia (isolate C23^T^) and the other in Spain (isolate HBSQ001), using very different isolation methods. The Australian isolate was obtained as a clonal population after serial-dilution to single cells and incubation for 3 weeks (extinction dilution method), while the Spanish isolate was recovered after serial enrichment over 2 years followed by colony purification on agar plates. Neither group aimed to select a specific strain or sequence type, but rather to isolate any member of the square haloarchaea of Walsby (SHOW group). The isolates had very similar 16S rRNA gene sequences, and a DNA-DNA cross-hybridization similarity of 80%, [8]. One notable difference between the isolates was in the structure of their cell walls. Strain HBSQ001 displayed an atypical triple-layered cell wall whereas C23^T^ possessed a conventional two-layered structure consisting of the cell membrane covered by a single, external protein S-layer [8].

The Spanish isolate (HBSQ001) was sequenced in 2006 [7], allowing the first description of its overall characteristics and general metabolism [12]. Its gene density is only 76%, much lower than in other haloarchaea and most prokaryotes, and this was attributed to a plethora of repeat sequences and pseudogenes. The reason for the low gene density is not clear. Features noted as likely to be related to survival up to salt saturation included (a) multiple uptake systems for phosphates and phosphonates, which are limiting nutrients in these environments (b) halomucin, an extraordinary long (9,159 aa), secreted protein that probably protects against cell desiccation, and (c) the presence of genes specifying two different bacteriorhodopsins (BopI, BopII).

Variation within a local population of *Hqr. walsbyi* has been studied by comparing the genome of HBSQ001 with environmental DNA sequences recovered from the same Spanish saltern from which this organism was isolated i.e., with autochthonous DNA [13], [14]. While divergence within the *Haloquadratum* affiliated population, as measured by the metagenomic 16S rRNA gene sequences, was very low (≤1.6%), comparison of the genomic and metagenomic sequences revealed both highly conserved and hypervariable regions, denoted as ‘genomic islands’. The variable sequences represent a pool of genes shared by some members of the population, the so called pan-genome [15] which, for this organism, was estimated to be at least another chromosome equivalent (3 Mb), and probably much more [14]. The world-wide diversity of this organism has also been examined, largely using 16S rRNA gene sequences, and these data also indicated that *Hqr. walsbyi* populations are highly coherent [16], with a level of variation ≤2%, a value very close to the 1.6% divergence observed within the Spanish saltern population. Not only was *Hqr. walsbyi* the dominant microbial group at these sites but it also appeared to be the *only* species of this genus. Further, within these datasets, near identical sequences could be found at distant sites. This picture of global conservation contrasts with other, environmentally common genera of haloarchaea, such as *Halorubrum* and *Haloarcula,* that show much higher and more usual levels of divergence in rRNA genes (∼7%). The latter genera have many recognised species, and environmental sequence studies indicate many additional species are yet to be isolated. Given this background, it was important to assess the true level of divergence of *Haloquadratum* by comparing the genome sequences of isolates recovered from geographically distant sites.

While hypersaline waters with over 30% w/v salt typically have high concentrations of prokaryotic cells (∼10^7^ ml^−1^), and even higher levels of virus particles (∼10^9^ particles ml^−1^), there are few if any grazing ciliates and flagellates [17]. Viruses are known to be a significant driving force in the evolution of prokaryotes, and in these environments they are the major predator of haloarchaea. Indeed, viral lysis of *Haloquadratum*-like cells in natural hypersaline water has been observed directly by electron-microscopy [18]. A recently recognised prokaryotic defence mechanism against foreign DNA, usually viruses and plasmids, is the CRISPR (clustered regularly interspersed palindromic repeat) system, which allows cells to specifically recognise and destroy target sequences, and in many respects parallels the function of the RNAi system of eukaryotes [19]. CRISPR systems are particularly common in Archaea. The dynamic nature of CRISPR arrays mean they show high sequence variability between members of the same species, and so can be used to type strains, or to assess the prevalent viruses at particular times, or between geographically distant sites [20].

About 14 genome sequences of haloarchaea are currently available (Mar, 2011), and this number should expand rapidly to over 100 (http://www.genomesonline.org/gold). However, the only comparison of closely related haloarchaeal species to date has been that of *Halobacterium salinarum* strains R1 and NRC-1, and these genomes are so closely related that they provided only very limited information on their mode of divergence [21]. In the current study, the genome sequence of the type strain of *Hqr. walsbyi*, isolate C23^T^, was determined and compared to a geographically distant isolate, HBSQ001. Overall, the two isolates are much less divergent than expected given the enormous distance between the sites of isolation and their very different modes of isolation. However, this high similarity enabled their differences to be mapped precisely. Genomic comparison, along with evidence from environmental DNA sequences, points to a rapid, global dispersal system for *Hqr. walsbyi*, acting to homogenize populations at distant locations. By inference, the predicted seeding by global dispersal must also lead to successful domination of the microbial populations in hypersaline waters around the world.

## Results and Discussion

### Sequence of the *Hqr. walsbyi* C23^T^ genome

The complete sequence was achieved using the previously determined sequence of HBSQ001 as a scaffold to arrange 220 separate contigs derived from 454 sequence reads. Contig gaps were then spanned by PCRs to close the genome. The general features of the circular chromosome, and three plasmids carried by this strain (EMBL accessions FR746099- FR746102), are presented in Table 1. For comparison, the corresponding data of the Spanish strain HBSQ001 are also included. The chromosomes of both are similar in size (∼3.1 Mb) and %G+C content (47.8%). Both have two rRNA operons and 45 tRNA genes, and carry a similar number of predicted ORFs (C23^T^: 2,894 and HBSQ001: 2,819). Curiously, the gene density is relatively low (79%) and there are over 300 pseudogenes (C23^T^, 337; HBSQ001, 314), comprised largely of degraded transposases and conserved hypothetical proteins. The strains differ in their plasmids, with C23^T^ carrying one large plasmid (PL100, 100 kb) and two small plasmids of around 6 kb (PL6A, PL6B) while HBSQ001 contains a single, unrelated 47 kb plasmid (PL47). Analyses of tetra-nucleotide frequencies show that the sequences CTAG, GGCC and AGCT are strongly avoided on the main chromosome. GGCC is also avoided on the large plasmid and is absent in the small plasmids (Table S1).

**Table 1:** General features of *Hqr. walsbyi* C23^T^ compared with those of strain HBSQ001.

	C23^T^				HBSQ001^a^		Totals	
Feature	Chromosome	PL100	PL6A	PL6B	Chromosome	PL47	C23^T^	HBSQ001
Length (bp)	3,148,033	100,258	6,129	6,056	3,132,494	46,867	3,260,446	3,179,361
G+C content (%)	47.8	43.9	51.1	52.0	47.8	47.7		
% coding (proteins/RNAs)	79.3	70.1	78.2	78.5	78.2	68.8		
Gene distance (Avge)	227	336	176	168	240	393		
Predicted ORFs	2,894	83	6	6	2,819	38	2,989	2,857
Pseudogenes	323	14	0	0	314	0	337	314
rRNA operons (16S, 23S, 5S)	2	0	0	0	2	0	2	2
tRNA genes	45	0	0	0	45	0	45	45
Other RNAs (7S, RNAseP)	2	0	0	0	2	0	2	2
Avge pI of proteins	5.1	5.0	4.6	4.6	5.1	5.2	5.1	5.1

a The data for HBSQ001 have been updated from the previous publication. The number of ORFs is reduced because pseudogenes are now counted as a single ORF even if they consist of several fragments.

### General organization of the chromosome of strain C23^T^

**Figure 1:**
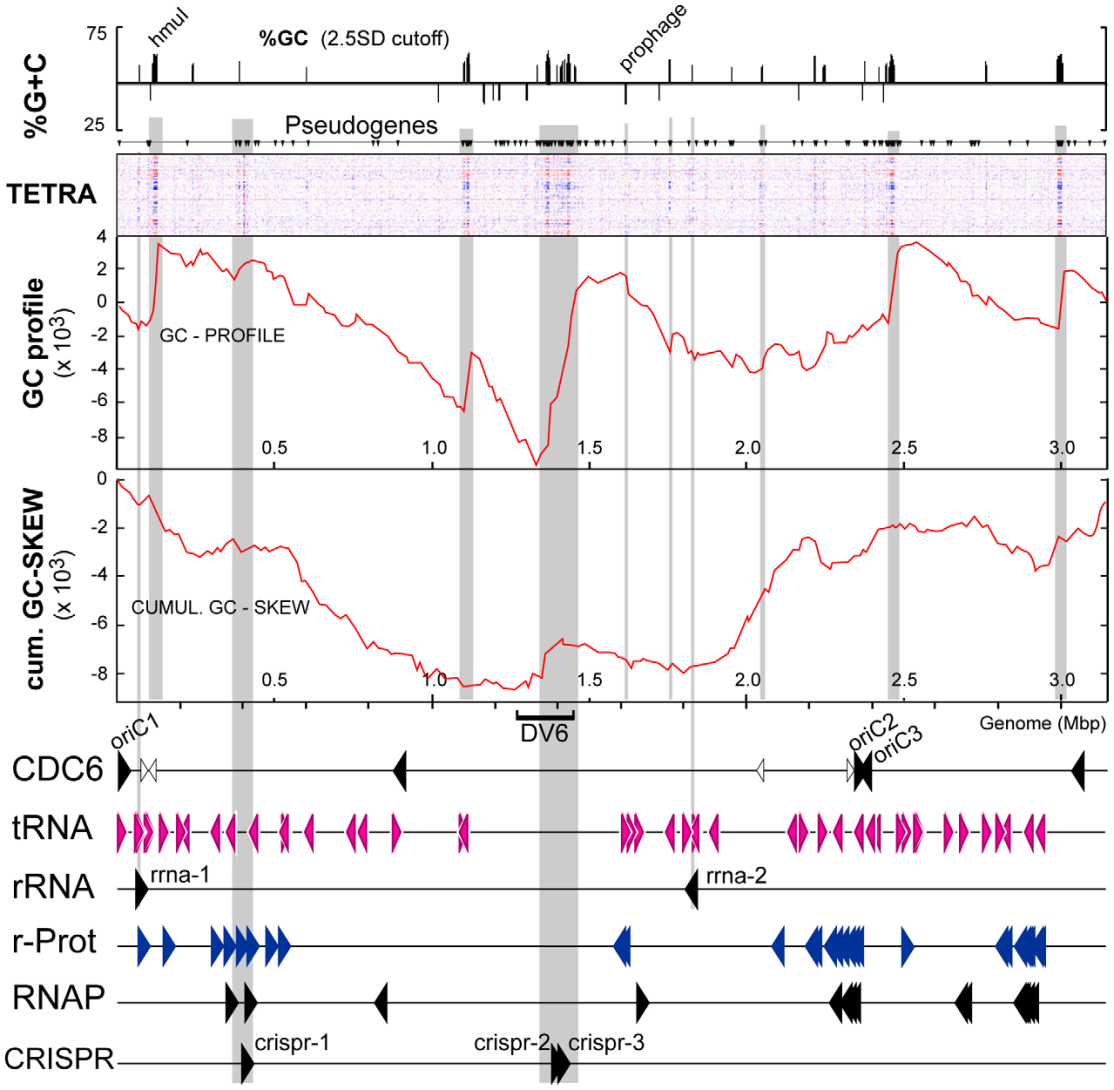
Features of the strain C23^T^ chromosome. The constant horizontal axis in all cases is the genome from left to right (first to last base of deposited sequence), with a scale given in Mbp. From top to bottom are plots of: (a) %G+C if the deviation for a 1 kb window is more than 2.5 SD from the average, (b) protein-coding pseudogenes (vertical triangles), excluding those of transposases, (c) variation in tetramer nucleotide composition (TETRA), where darker colors indicated more prominent deviation, (d) GC-profile, (e) cumulative GC-skew, (f) positions and orientations of the following gene categories: CDC6, *orc1*/*cdc6* homologues; tRNA, transfer RNA genes; rRNA, ribosomal RNA operons; r-Prot, ribosomal protein genes; RNAP, RNA polymerase subunit genes; CRISPR, loci of clustered regularly interspersed short palindromic repeats. Smaller, unfilled arrowheads in the CDC6 line represent the positions of *cdc6* pseudogenes. DV6 (divergent region 6, see Figure 5) is indicated below the cumulative GC-skew plot. Vertical grey-shaded stripes mark correlating features.

Figure 1 presents the results of several global analyses of the main chromosome of C23^T^. Major deviations from the average %G+C content (topmost plot) correlate closely with changes in tetramer frequency, as shown by the intense vertical bands in the TETRA plot below (third level). The second level graph shows the distribution of pseudogenes derived from non-transposase ORFs, and these are clearly associated with many of the variant regions identified in the adjacent TETRA and %G+C plots. Bacterial genomes often show large-scale organizational patterns, such as a systematic bias in the nucleotide composition of their leading and lagging strands, preferential placement of ORFs on the leading strand, and highly expressed genes close to the replication origin [22], [23]. In such cases, a plot of cumulative GC-skew versus genome position can show a simple, geometric pattern where the replication origin and the terminus occur near major inflections [24], [25]. In general, statistical deviations are much weaker in archaea so that cumulative GC-skew plots do not give a simple pattern (fifth level of Figure 1), nor does the GC-profile graph, a type of cumulative GC-skew that is more sensitive to local changes in %G+C content [26], shown just above it. However, in comparing the plots from the two strains (see later) one can distinguish between strong local deviations due to insertion of foreign DNA and weak positional deviations related to replication origins. Strong changes in the GC-profile correlate well with significant alterations in %G+C and tetramer composition. These atypical genome regions represent a mixture of unusual genomic features, described in detail below. Two prominent features are, (1) near the left end of the %G+C panel a distinct peak of higher %G+C, labeled *hmuI* (corresponding to a very long ORF encoding the halomucin gene with a highly biased codon usage) and, (2) a peak of lower %G+C at around 1.6 Mb that corresponds to a prophage, integrated into a tRNA gene.

Although the GC-skew plots were not able to directly identify likely replication origins, archaeal origin-of-replication sequences (*ori*) typically occur adjacent to Cdc6/Orc genes [27], [28], [29]. Haloarchaea can carry up to 17 copies of such genes [30] but the number of origins is usually much lower [29]. Strain C23^T^ possesses nine chromosomal Orc/Cdc6 genes, five of which appear to be complete (solid black arrowheads, CDC6 plot in Figure 1), and four are pseudogenes (smaller, unfilled arrowheads). In addition, two other Orc/Cdc6 genes are carried on the large plasmid, PL100 (see below).

The protein sequence of the first chromosomal Cdc6 ORF, Hqrw_1001, is most closely related to HVO_0001 of *Hfx. volcanii* and VNG2411G/OE4380F of *Hbt. salinarum*, both of which lie next to the main replication origin, *oriC1* [28]. The ORFs surrounding Hqrw_1001 are also very similar to those found near *oriC1* in other haloarchaea [21], [28], [29]. Upstream of Hqrw_1001 is the probable *oriC1* of *Hqr. walsbyi* C23^T^, characterized by sequence elements typical of replication origins of haloarchaea (Figure S1), including long inverted repeats surrounding an AT-rich sequence (potential DNA unwinding domain, DUE) [27], [28], [31]. Apart from Hqrw_1001, only two other complete homologues have sufficient, non-coding upstream sequences to contain potential further origins, Hqrw_3381 and Hqrw_3385. These lie very close to each other, are inward facing, and have *ori*-like sequence motifs nearby (Figure S1). These may represent one or two additional origins, and have been labeled *oriC2* and *oriC3* in Figure 1. As shown in the fifth and sixth panels of Figure 1, the predicted replication origins are near high points in the cumulative GC-skew graph, while the minimum (∼1.2 Mb) occurs almost mid-way between *oriC1* and *oriC2*/*oriC3*, and is likely to include a site or region where replication terminates. Multiple replication origins are common in Archaea [28], [32].

All 45 tRNA genes are located on the main chromosome. Rather than an even distribution, they show some tendency to cluster near the predicted replication origins, and to avoid the region furthest away from the origins, where replication is likely to terminate (1.1–1.6 Mb). In contrast to the relatively relaxed organization of tRNA genes in *Haloquadratum*, thermophilic Archaea such as *Sulfolobus* display a strong clustering of tRNA genes [33]. There are two rRNA operons in *Haloquadratum*, the first (at ∼0.07 Mb) faces away and is very close to *oriC1*. The second (at ∼1.8 Mb) is almost diametrically opposite the first on the circular chromosome but is some distance from the potential *oriC2/oriC3* at ∼2.4 Mb, again facing away. The two 16S rRNA genes differ by 3 bases while the corresponding 5S and 23S rRNA genes are identical. The next two lower panels in Figure 1 show the positions of protein-coding genes that are likely to be highly expressed; those for ribosomal proteins and RNA polymerase subunits. Their distribution is similar to the tRNA genes. The three CRISPR loci (crispr-1, -2 and -3) are indicated in the lowest panel of Figure 1. They occur within extended genomic regions of distinct tetra-nucleotide frequency, indicating foreign DNA. Overall, the genome appears to show some degree of global modeling, oriented around the predicted replication origins, but disturbances occur in regions likely to represent insertions of foreign DNA.

### tRNAs and codon usage

*Haloquadratum* is unusual in having a genome with a %G+C that is about 20 percentage points lower than all other genera within the family *Halobacteriaceae*. The most parsimonious explanation is that *Haloquadratum* has evolved from the higher level down to its current value. As expected from such a change, *Hqr. walsbyi* shows a strong bias towards codons with A or T in the 3^rd^ position, relative to the codon usage of other haloarchaea (ca. 60% A+T *vs* ca. 20%), while the bias is only slight for the 1^st^ and 2^nd^ position [7]. The strength of this bias indicates a rapid evolutionary drift towards a lower GC content. Due to this strong bias, ten different amino acids in this organism have NNT as the preferred codon in their synonymous codon sets (Table S2) [7]. However, as noted in the literature of codon-anticodon interactions, tRNA anticodons with A in the first position (which decode NNT codons), are generally uncommon, and in *Haloquadratum* there are no such tRNAs present in the genome. This means that despite a preference for NNT codons, all of these must be decoded by tRNA anticodons using G:U base-pairing, highlighting the view that “the anticodon-codon wobble base pair of a G_34_ with a U3 … is almost isomorphic/isosteric to a Watson-Crick base pair.” [34]. tRNA base modifications also determine specificity, and have been examined best in *Haloferax volcanii* [35], but not yet in *Haloquadratum*.

Two tRNAs contain introns (tRNA-Trp, tRNA-Met2), while tRNA-Ile1 has a CAT anticodon that is modified [36], enabling it to switch its specificity and act as a TAT anticodon, and decode Ile (ATA) codons. The genome also contains two partial tRNA genes that are associated with prophages (see below).

### Plasmids

**Figure 2:**
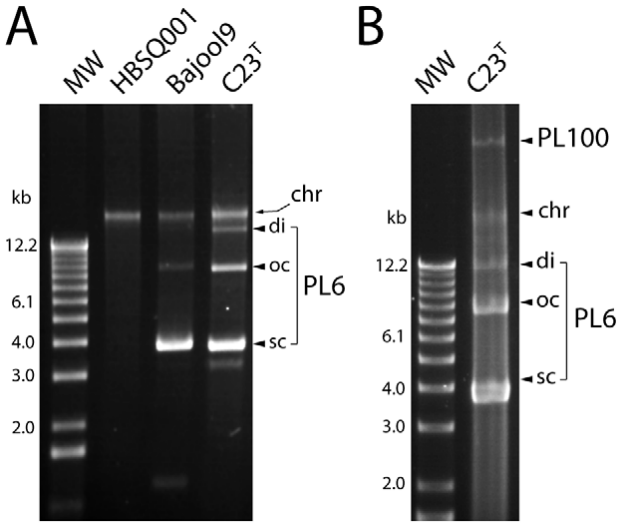
Plasmids of strains C23^T^, HBSQ001, and Bajool9. Plasmid preparations from the three strains of *Haloquadratum* were separated by electrophoresis on 1% agarose gels and stained with ethidium bromide. Panel A: the plasmid bands labeled PL6 are super-coiled (sc), open-circular (oc) and dimeric forms of two, closely related, ∼6 kb plasmids (see text for details). The Bajool9 isolate of *Haloquadratum* [16] is also seen to carry a similarly sized plasmid as well as a much smaller one, running near the bottom of the gel. The position of contaminating, sheared chromosomal DNA is indicated at the right of each panel (chr). Panel B: more of the plasmid preparation from isolate C23^T^ has been loaded, revealing the large plasmid, PL100. In both panels, MW indicates the 1 kb DNA size ladder (Invitrogen), with lengths indicated in kb at the left.

Plasmid extracts of *Hqr. walsbyi* C23^T^ cells revealed a small, 6 kb multi-copy plasmid (∼30 copies/genome equivalent) designated PL6, and a much larger, low copy-number plasmid, PL100 (Figure 2A and B). Panel A shows a typical gel profile of un-restricted plasmid DNA, with the smaller plasmid band displaying super-coiled, open-circular and dimeric forms. Loading more plasmid extract (panel B) revealed the large plasmid, PL100, running above the fragmented chromosomal DNA band. Plasmid preparations from the previously sequenced Spanish isolate, HBSQ001, and from of a novel, Australian isolate of *Haloquadratum*, strain Bajool9 [16], were also included in Panel A. The Spanish strain lacks small plasmids and its large 47 kb plasmid (PL47) is not visible in this gel. The Bajool9 isolate was recovered from a saltern crystallizer in Bajool, Queensland, Australia [16], some 1700 km north of the crystallizer from which C23^T^ was isolated. It also harbours a ∼6 kb plasmid, as well as another, much smaller plasmid (faintly visible at the bottom of the lane, running between the 1 and 2 kb size markers).

### Sequences of the small plasmids of strain C23^T^

**Figure 3:**
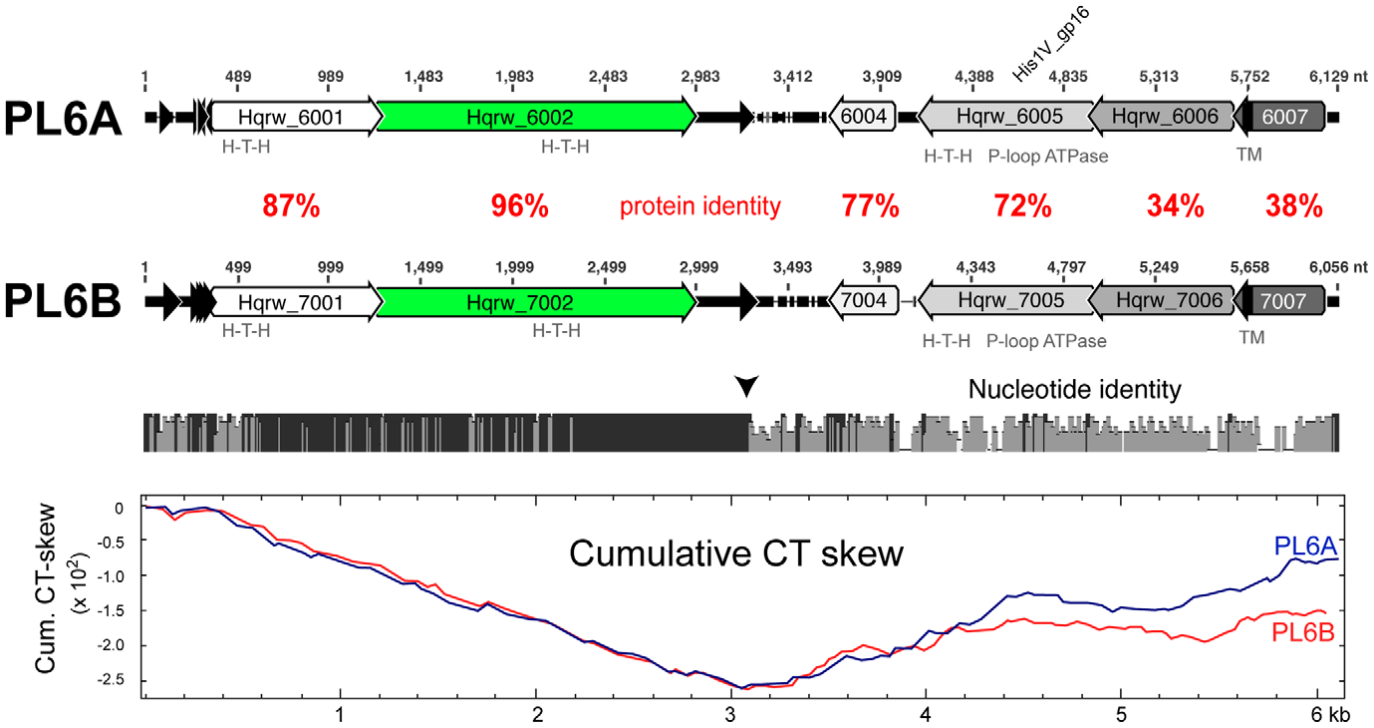
Comparison of circular plasmids PL6A (6,129 bp) and PL6B (6,056 bp). Predicted ORFs are labeled with their locus tags, and arrows represent their orientations and lengths. Nucleotide positions are given above each plasmid. Nucleotide similarity is indicated by the bar chart below the alignment (bar height and darkness of colour indicate the level of nucleotide identity, with solid black indicating 100% identity). The vertically oriented arrowhead at around 3.2 kb indicates a sharp break in homology. Percentage identity values of the corresponding protein sequences (in red) are given between ORFs in the alignment. H-T-H, helix-turn-helix protein domain. TM, transmembrane domain. Solid black arrowheads represent short repeat sequences (see text). The lowest panel shows the cumulative CT-skew plots of both sequences.

Genomic sequencing revealed that the PL6 plasmid band observed in C23^T^ consists of two closely related plasmids, designated PL6A and PL6B. These were in approximately equal proportions in the population, as judged by restriction digests and sequenceing reads (data not shown). Their %G+C is significantly higher than that of the chromosome (51–52% versus 47.8%). They show high sequence identity, particularly over the first two ORFs, but this drops sharply at around 3.2 kb, (arrowed in Figure 3), following immediately after a motif consisting of two direct repeats of the sequence ACAGATTA bordered by an inverted repeat. Both plasmids have six predicted ORFs that show a similar organizational pattern. The corresponding ORFs between plasmids are predicted to code for proteins of similar size and amino acid sequence (Figure 3).

The six annotated ORFs of PL6A or PL6B show few protein motifs that could give clues to function. None show significant homology to any of the known Rep, Cdc6, MCM helicases or DNA polymerases, nor are there any detectable *oriC1*-like origin-of-replication-box (ORB) motifs, so the actual mode of plasmid replication remains uncertain. However, archaeal plasmids are known to display considerable variation in their replication systems [37], [38]. Helix-turn-helix motifs, indicative of DNA binding, are predicted for Hqrw_6001, 6002 and 6005 (and the corresponding PL6B ORFs), and the latter protein also contains a P-loop ATPase-like motif (see also below). Intriguingly, Hqrw_6007 and Hqrw_7007 were detected by mass spectrometry in purified cell membrane preparations of C23^T^ (Table S3), a cellular location consistent with their predicted C-terminal membrane anchors. The N-termini of these proteins are similar to members of the CopG/MetJ family of repressors, indicating probable binding to target sites on PL6. If so, the bifunctional properties of these proteins suggest they could tether the PL6 plasmids to the cell membrane. Repeats and inverted repeat sequences occur within the two long intergenic regions found between Hqrw_6007 and Hqrw_6001, and between Hqrw_6002 and Hqrw_6004. A long, inverted repeat occurs in both plasmids at a similar position (nt 107–123, PL6A), and is flanked by ORFs with predicted DNA binding motifs. Cumulative nucleotide skew plots that include thymidine show a strong inflection in the intergenic sequence at about 3.1 kb (a highly conserved region), with a maximum in the intergenic sequence between the sixth and first ORFs (Figure 3). The arrangement of these conserved repeats at almost diametrically opposite sites (0.1 and ∼3.2 kb), and their correlation with inflections in nucleotide skew, suggest they may represent replication origins and termini (assuming a theta mode of replication).

The nucleotide sequences of the PL6 plasmids were not similar to the main chromosome, or to PL100 of C23^T^, or to the genome of HBSQ001, or other reported haloarchaeal plasmids. Partial sequencing of the 6 kb plasmid in the Bajool9 strain revealed it to be similar in sequence and gene organization to PL6A or PL6B (85% nt identity, data not shown). BLASTN searches of the CAMERA metagenomic database gave numerous matches to DNA sequences recovered from a saltern crystallizer pond in the USA (Table 2). Hundreds of matches were found in the larger dataset of environmental sequences recovered from Lake Tyrrell, a hypersaline lake in Australia (Table 2). Sequence reads from this dataset could be retrieved that showed high nucleotide identity to PL6, and these could be assembled into contigs of up to 6 kb that displayed co-linear sequence similarity to these plasmids (data not shown). While no matches to metagenomic data from the Santa Pola saltern in Spain were observed, recently available sequences from a viral metagenome of the same saltern showed several TBLASTX matches to PL6 ORFs Hqrw_6005 and Hqrw_7005 (see later). The presence of *Hqr. walsbyi* 16S rRNA gene sequences in the metagenomic data from the three sites confirmed the presence of this species in all three datasets. The data indicate that PL6-like plasmids are widely distributed.

**Table 2:** PL6 plasmid related sequences in metagenomic data.

Sample (site)	Accession/Project^a^	Reference	BLASTN hits^b^
Saltern metagenome (San Diego Bay, USA)	CAM_PROJ_SalternMetagenome^a^	[79]	11 (PL6A), 16 (PL6B)
Saltern metagenome (Santa Pola, Alicante, Spain)	Metagenome of Marine NaCl-Saturated Brine^a^	[14]	0 (PL6A, PL6B)
Metavirome (Santa Pola, Alicante, Spain)	GU735174, GU735304, GU735358, GU735225, GU735307, GU735310^c^	[51]	TBLASTX, E ≤10^−6^
Salt Lake metagenome (Lake Tyrrell, Victoria, Australia)	JCVI^d^ (GS–84, 2005)	JCVI, unpublished data	719 (PL6A), 644 (PL6B)

a CAMERA database, http://camera.calit2.net/about-camera/full-datasets.

b matches with expect values better than 10^−10^.

c All six matching sequences were to Hqrw_6005/Hqrw_7005.

d JCVI, J. Craig Venter Institute, USA. Data to be released in 2011 (personal communication, Matt Lewis, JCVI).

**Figure 4:**
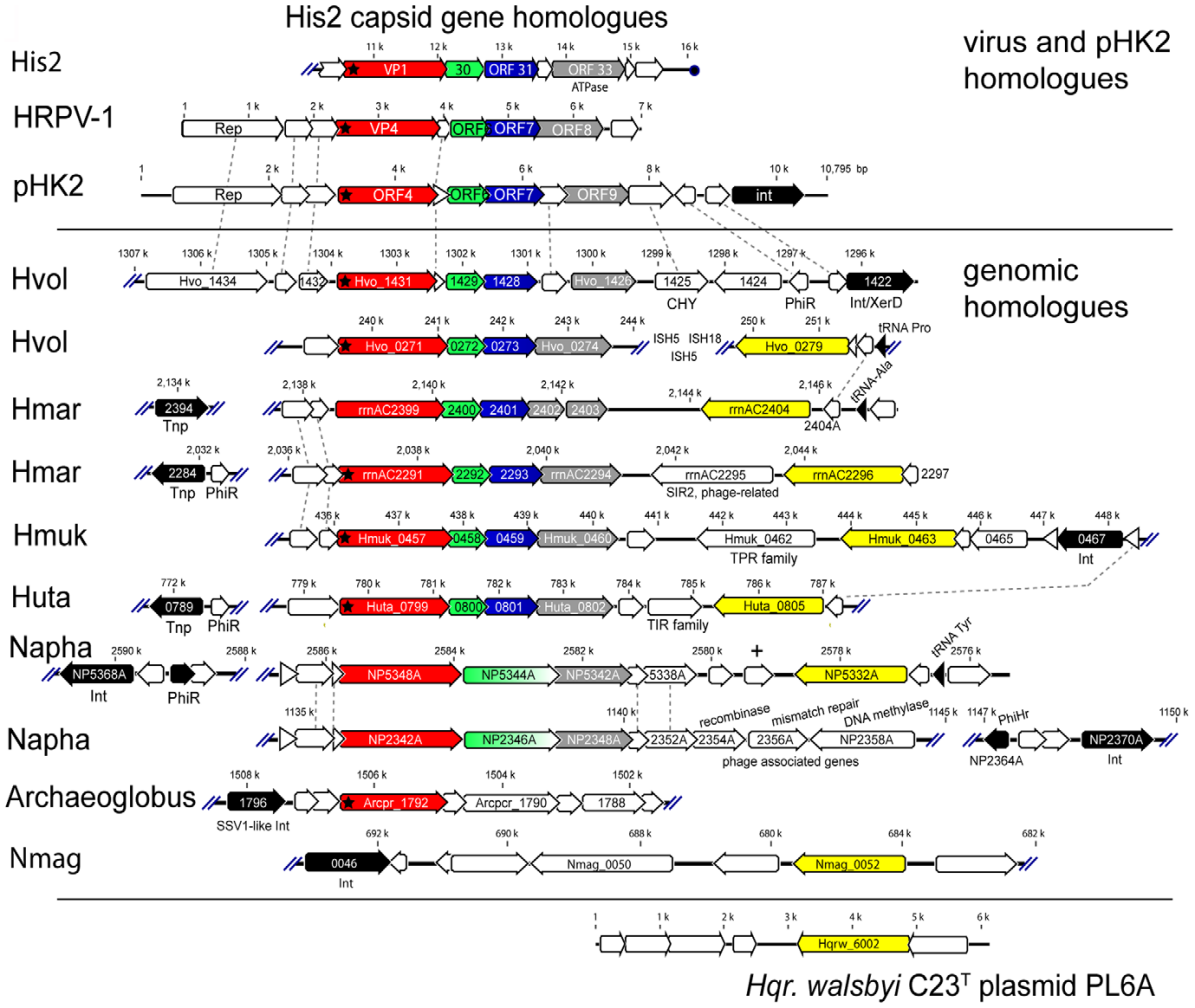
Plasmid PL6-associated virus genes in haloarchaea. Alignment of genomic regions of *Haloarcula marismortui* (Hmar), *Haloferax volcanii* (Hvol), *Halomicrobium mukohataei* (Hmuk), *Halorhabdus utahensis* (Huta), *Natronomonas pharaonis* (Napha), *Archaeoglobus fulgidus* (Archaeoglobus) and *Natrialba magadii* (Nmag) with the corresponding genes of haloviruses His2, HRPV-1 and plasmid pHK2 (top) and *Hqr. walsbyi* C23^T^ plasmid PL6A (bottom). Locus tags or ORF numbers are given for most ORFs. Homologues of the capsid protein genes of His2 are colour coded (red, green, blue and grey). Homologues of PL6 ORF2 genes are coloured yellow. Integrase (Int) and transposase (Tnp) genes are coloured black. Dashed lines indicate related genes (other than those colour coded) that occur between two or more genomes. Asterisks within the His2 VP1 ORF and its homologues indicate a predicted signal sequence. Genomic position scales are indicated for each genome in kilobase pairs.

Comparing PL6 to the genome sequences of other microorganisms, the second ORF (Hqrw_6002/7002) gave significant matches to numerous archaeal homologues. In their genomic contexts, these homologues are frequently adjacent to a previously described group of well-conserved halovirus genes, initially identified from the sequence of halovirus His2 [39]. A block of four genes including that for the major virus capsid protein of His2 is found in many haloarchaeal genomes as well as pHK2, a small haloarchaeal plasmid. Although these genomic homologues do not appear to be His2-like prophages [39] their distribution and conservation somehow relates haloviruses and haloarchaeal plasmid genes at specific sites of genomic integration [39], [40], [41]. Two recently described pleomorphic haloviruses, HRPV-1 and HHPV-1, also contain a similar block of homologues related to His2, plasmid pHK2 and the same genomic loci [42], [43], further consolidating this link. A summary of the relationships between PL6, the three haloviruses, and the related loci from six different genera of haloarchaea is presented in Figure 4. In most cases, homologues of PL6 ORF Hqrw_6002 are found either immediately adjacent to the His2-related gene cluster or separated by a small number of intervening genes. In the case of *Hfx. volcanii*, the intervening genes are three consecutive insertion sequences. *Archaeoglobus*, which is not a member of the family Halobacteriaceae but belongs to the same archaeal phylum (Euryarchaeota), also contains a His2 VP1 homologue, which is near a virus integrase. The proximity, consistent orientation, and wide phylogenetic distribution of this gene arrangement speaks of an important and complex network of DNA cross-talk; one that spans many of the known genera of haloarchaea and involves a diverse group of plasmids and haloviruses. In some respects, these appear similar to Mavericks, a class of mobile elements found in Eukaryotes [44].

The PL6 homologues Hqrw_6005 and Hqrw_7005 were specifically related to halovirus His1 protein His1V_gp16, and to an ORF of *Nmn. pharaonis* (NP3284A). The latter ORF occurs within a 13 kb region that also includes a CRISPR system, and which has been inserted into a chromosome-integrated copy of the *Nmn. pharaonis* plasmid PL23. All these homologues possess a conserved P-loop ATPase motif. Halovirus His1 is the type species of the Salterprovirus group, to which halovirus His2 also belongs [39], so the relationship observed in this study between PL6, His1, and genomic loci carrying His2-related genes provides further support for a virus link between these elements. Indeed, there is a strong possibility that PL6 is a provirus.

We propose that this family of archaeal genomic loci that include halovirus, and now PL6 homologues, be referred to as virus and plasmid related elements (ViPREs). Understanding the mechanisms, significance and impacts of ViPREs on the evolution of haloarchaea (and perhaps other *Euryarchaeota*) and their viruses is clearly a high priority.

### Plasmid PL100

The large plasmid of C23^T^ is 100,258 bp in length, 4 percentage points lower in G+C than the main chromosome (43.9%) and predicted to code for 83 ORFs. It is more than twice the size of the 47 kb plasmid in HBSQ001 (PL47), and although the two plasmids appear to be largely unrelated they both carry a preponderance of hypothetical or conserved hypothetical proteins without discernable function. ORFs with assigned function on PL100 include those involved in plasmid maintenance or replication, restriction endonucleases, methylases and helicases. Two *cdc6* gene homologues, Hqrw_5030 and Hqrw_5083, are situated approximately opposite each other on the plasmid, and their upstream regions possess ORB-like repeat motifs consistent with archaeal replication origins (Figure S1, OriP1 and OriP2). Nucleotide skew analyses indicated only small inflections near both, but as there are no RepH homologues, replication is most likely initiated near these *cdc6* genes.

Plasmid PL100 was unstable in laboratory cultures of C23^T^, and was lost from the culture used to prepare DNA for genome sequencing. Some sequences of PL100 had been recovered from an earlier, preliminary genome sequencing effort, and this allowed PCR primers to be designed to detect the presence of the plasmid. Screening cultures for successful PCR amplification identified one (out of 6) that had retained the plasmid, and DNA from this source was used to PCR amplify across contig gaps and complete the sequence (see Materials and Methods). From these observations, PL100 is readily dispensable, at least under laboratory conditions.

### Comparative overview of the chromosomes of C23^T^ and HBSQ001

**Figure 5:**
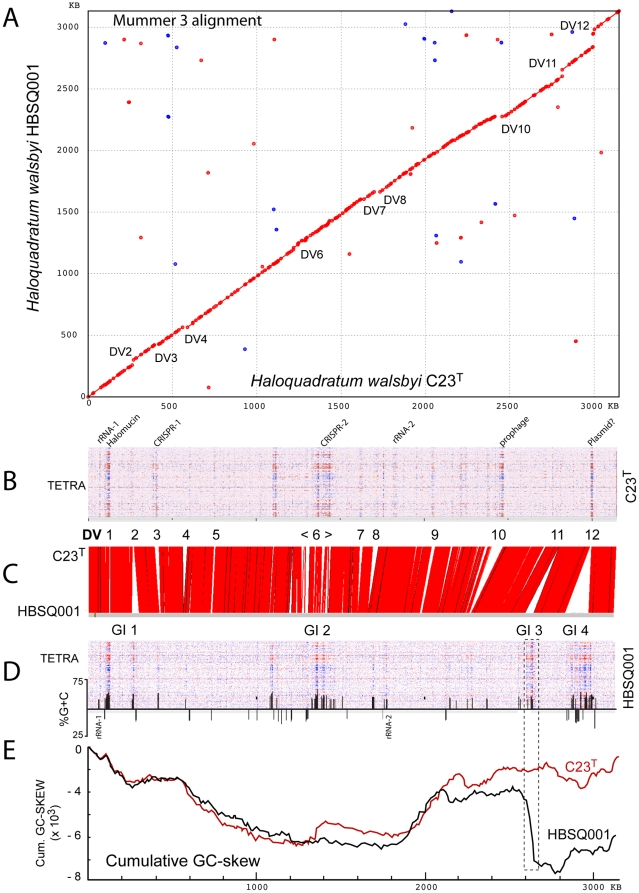
Comparison of the chromosomes of *Hqr. walsbyi* strains C23^T^ and HBSQ001. Panel A: Mummer nucleotide alignment, where dots indicate similar sequences in the same orientation (red), or reverse orientation (blue), shared by the two strains. Panels B and D: Tetra-nucleotide variation (TETRA) along the chromosomal sequences of each strain (labeled). Several divergent regions in each plot are labeled in both panels (see text). The %G+C deviation (if a 1 kb window is more than 2.5 SD from the average) is given for HBSQ001 immediately below the panel TETRA plot of panel D. Panel C: comparison of the chromosomal sequences using the Artemis comparison tool (ACT). Red lines indicate regions of high nucleotide similarity; white regions indicate lack of similarity. Above this plot regions of major divergence (DV 1-12) are indicated (and also on the Mummer plot in panel A), while below the previously described genomic islands (GI 1-4 [13]) are indicated. Panel E: Cumulative GC-skew plots for the chromosomes of C23^T^ (red) and HBSQ001 (black). The boxed region extending upwards to panel D outlines the major drop in the cumulative skew plot of HBSQ001.

The close relationship of C23^T^ and HBSQ001 is clearly seen in a nucleotide alignment of the two chromosomes (Figure 5), where only minor shifts from a straight line due to indels occur; up-shifts indicating insertions in HBSQ001 and downshifts for insertions in C23^T^. There are no large-scale rearrangements – the two nucleotide sequences are co-linear, sharing 98.6% nucleotide similarity. Panel C is a different representation of the aligned chromosomes using the Artemis Comparison Tool (ACT) that shows pattern of variation between the chromosomes. Together with panel A, the two graphs present a typical pattern of intra-species variation seen in many prokaryotes: a backbone core sequence with high similarity that is interspersed with strain-specific indels.

Twelve prominent regions of strain-specific variation (divergent regions DV1 - DV12) are indicated above the ACT alignment. Below the alignment, the positions of genomic islands (GI 1-4) of HBSQ001, as described previously by Cuadros-Orellana *et al.* [13], are shown. Genomic islands are regions of high variability that were identified when the HBSQ001 genome was compared to autochthonous, metagenomic DNA sequences. The DV and GI sequences are discussed in more detail below, but it is clear that the GIs also correspond to regions of major difference between HBSQ001 and C23^T^ seen in the current study, i.e. GI I  =  DV2, GI 2  =  DV6, GI 3  =  DV11, GI 4  =  DV12. Panels B and D plot the variation in tetramer frequency of the two strains, and show that some regions of divergent sequence composition are common to both (such as the halomucin genes at around 0.6 Mb, DV1) while others are strain-specific, often coinciding with large indels and indicative of foreign DNA insertions. Panel E compares the cumulative GC-skew plots of both chromosomes, which show major differences at positions corresponding to large indels. The dramatic drop in the HBSQ001 plot at around 2.6 Mb is due to a strain-specific integration of a prophage (see below). The prophage provides HBSQ001 with two additional *cdc6* genes compared to C23^T^, but these are closely spaced and outward facing, and are not associated with nearby *ori*-like sequence motifs. The replication origins used by HBSQ001 are probably the same as the corresponding sequences in C23^T^, as these are conserved, but the *cdc6* gene (HQ2952A) corresponding to that near *ori*C2 of C23^T^ has been inactivated by an insertion. The adjacent *ori* could still function, as replication origins in haloarchaea can use Cdc6 proteins synthesized from genes other than the adjacent copy [29].

### Systematic comparison of the chromosomes of C23^T^ and HBSQ001

The close similarity of the two strains enabled a systematic and precise analysis of all the differences between their chromosomes. A custom PERL-based script (as outlined in Materials and Methods) was applied that could perform a base-by-base comparison of very similar sequences while being tolerant to insertions and deletions of any size as well as large sequence duplications. Trivial differences between the strains were excluded (e.g. point mutations), based on criteria defined in Materials and Methods. Although the constraints were relatively relaxed, the resulting set contained only 512 non-trivial differences, emphasizing the very close relationship of the two strains. These differences were manually inspected to eliminate simple cases of regions with enhanced sequence divergence, and this left a total of 360 differences, representing the final set of strain-specific sequences (Tables S4 and S5).

The remainder of the two chromosomes defines the common shared sequences of the two strains, comprising about 84% of each genome. These sequences are 98.6% identical at the DNA level and are absolutely synteneic. They do not show even a single genome rearrangement (inversion or transposition). Sequence identity remains high even in intergenic regions (which represent a significant proportion of the genome in *Haloquadratum*) and in genes that are not well conserved among haloarchaea. Therefore, we prefer the term “shared sequence” instead of “core sequence” as the latter implies restriction of the analysis to only the well-conserved sequences of both genomes.

In other studies comparing strains of the same prokaryotic species, core sequence similarities of 98–99% are commonly observed but with intergenic regions excluded. Also, these genomes usually show inversions and rearrangements. For example, in *E.coli*, a recent study of 61 genomes found that they can vary in size by more than 1 Mb (4.6–5.7 Mb), that gene order is not strongly conserved (i.e. extensive inversions/rearrangements), and that any particular *E. coli* genome contains only about 1/5th of the core genes of the species, while the remaining 4/5ths sample a pan-genome estimated to contain about 15,000 gene families [45]. In the current study, the complete lack of genome rearrangements together with a 98.6% sequence identity, even when intergenic regions are included (which amount to more than 20% of the sequence), indicate a remarkable level of similarity between the two strains of *Haloquadratum walsbyi*, particularly given that they originate from samples taken at near-maximal distance on Earth.

Strain-specific sequences contribute 16% of the genetic material in each strain, and include numerous integration and excision events. It is an apparent paradox that so many of these events have occurred while the shared sequences do not even show a single large-scale genome rearrangement. The availability of such highly conserved genome sequences, with synteneic shared sequences, provided an outstanding opportunity to examine, in fine detail, the differences between these strains. This revealed two unsuspected biological processes that have moulded these genomes: (a) repeat-mediated deletions and (b) deletion-coupled insertions. These processes, which are described in detail below, were able to be discerned with such clarity because (i), the positions of insertions and deletions could be determined unambiguously to single-base resolution, and (ii), the number of individual cases is large enough to exclude the possibility that they were random events.

### Classifying strain-specific sequences

**Figure 6:**
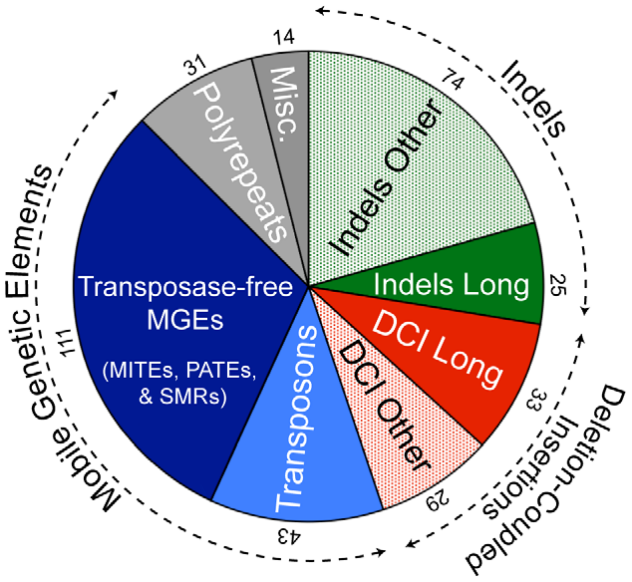
Categories of strain-specific regions, plotted according to the relative numbers in each type. Pie chart showing the types of strain specific regions in both strains. Blue: mobile genetic elements, which includes transposons and transposase-free mobile elements (MITEs, PATEs, and SMRs). Green: insertions and deletions (indels). Red: Deletion-Coupled Insertions (DCI). Long indels and DCIs (> 1.5 kb) are filled colour sectors while medium (0.15–1.5 kb) or short (20–150 bp) sequences in these categories are dotted. Numbers at the outer edge of each sector are the numbers of cases in each category. Data from Table S4.

Each of the 360 strain-specific sequences represents some type of insertion or deletion (indel), which could be classified into one of several categories (Figure 6, Table S4). About half of the indels represent mobile genetic elements (see below) while the other half are unrelated to known mobile genetic elements. The smallest indels are just longer than 20 bp (the lower cut-off size for manual inspection) while the largest is more than 100 kb (in DV12). Some of the short indels were sub-classified as “polyrepeats”, and consist of a short (e.g. 6-mer) repeated sequence (allowing for some variability), where the number of copies differs between the two strains. For polyrepeats, an exact point of insertion/deletion cannot be defined. Several polyrepeat indels are located within coding regions, where the proteins from the two strains have e.g. dipeptide repeats of different length but with the reading frame conserved.

### Deletion-coupled insertions occur at identical positions in the shared sequence

An unexpected observation was that the majority of the insertions are coupled to deletions such that the two strains possess unrelated DNA sequences integrated at exactly the same position within the chromosome. Deletion-coupled insertions differ from “hot-spots of integration” where insertions and deletions usually occur within a short distance but commonly at distinct positions. Of several possible explanations for this phenomenon, we favour a process where insertion and deletion are mechanistically coupled, so resulting in unrelated sequences at exactly the same position.

A random process can be excluded for statistical reasons, as 64 independent deletion-coupled insertions were identified (Figure 6, Table S4), and these showed no evidence of sequence conservation that could be interpreted as a targeting. The deletion-coupled insertions are also unlike mobile, site-specific pathogenicity islands (PI) found in bacteria [46], as those occurring at the same site are usually closely related and characteristically produce direct repeats upon integration [46], [47]. The results of the current study indicate that deletion-coupled insertions originate by an as yet undescribed mechanism that couples the insertion of foreign DNA to the simultaneous loss of chromosomal genes, an apparently risky strategy for individual cells as it removes part of their own genome. Our data imply that the majority of the insertions of foreign genetic material are coupled to a deletion.

Insertions and associated deletions varied widely in size, with the smallest described being just over 20 bp, the applied minimal cut-off (although even shorter replacements were observed). Both, the deleted sequence and the replacing inserted sequence may be long (16–43 kb), while the longest example was 104 kb (in HBSQ001), corresponding to a 429 bp unrelated sequence in C23^T^. Some are likely to have a major biological impact, for example, the exchange of the S-layer glycoprotein in HBSQ001 occurs on a 43.7 kb *vs* 8.2 kb deletion-coupled insertion region (DV2) that has been reported as genetic island GI1 [14]. Ín this case, the deleted/inserted genes are homologous and thus deletion-coupled insertion results in what has been described as divergent genes in a conserved synteneic context [13]. The exchange of the cell surface glycoprotein may be responsible for the observed morphological difference between the cell walls of the two strains [8]. The Cas/CRISPR systems for virus/plasmid defence, which are exclusively found in C23^T^, are also located on such deletion-coupled insertions (DV3 and DV6).

### A novel, repeat-mediated deletion mechanism

Another unexpected finding are differences that we interpret as resulting from repeat-mediated deletion events, where a sequence of usually <20 bp in one strain is found directly repeated at both ends of a strain-specific sequence while only a single copy of the repeat exists in the corresponding position in the other strain (Table S6). In the analyzed set, there are 16 independent cases of such indels, with direct repeats ranging from 10–19 bp, and an additional 11 cases involving repeats ranging from 7–9 bp. There are also examples where the repeats are 6-mers (3), 5-mers (5) and 4-mers (6). Although short direct repeats such as 4-mers may occur by chance, less than one case (0.4) would be expected in a set of 99 indels.

A sequence that occurs in only one strain represents an “indel” as there is no *a priori* information to indicate whether it is due to an insertion in one strain or a deletion in the other unless a distinction can be made based on additional information, such as gene truncation, preservation of conserved gene clusters, or tetra-nucleotide analysis. In nearly all repeat-associated indels (10 out of 11) where a distinction between deletion and insertion was possible, a deletion was identified as the cause.

Repeat-mediated deletion events appear to be common among copies of the small mobile element HqIRS46, which contains a direct repeat of 22 bp length (Table S6). Repeat-mediated deletion shortens the element of 390 bp to concatenated terminal sequences of 52 bp.

In a comparison of the genomes of *Hbt. salinarum* strains NRC-1 and R1, three indels were noted [21] that probably also represent repeat-mediated deletions. One was a precise deletion of a copper-binding domain of the HcpB protein in NRC-1 (via a 32 bp direct repeat). The second was a 133 bp deletion in the promoter of the rRNA operon in R1 (via a 27 bp direct repeat). The third case, a 10007 bp strain-specific sequence (flanked by an 8 bp repeat) present only in NRC-1, was originally believed to be an insertion in the latter strain. Subsequent low pass sequencing of other *Halobacterium* strains has shown that this is a deletion in R1 (F. Pfeiffer, D. Oesterhelt, unpublished results). These examples of repeat-mediated deletions extend this biological principle to a different genus within the *Halobacteriaceae*, suggesting that they are widespread and possibly much more common than previously realised.

These types of events are reminiscent of the repair of double-strand breaks (DSB) by a pathway described as micro-homology-mediated end-joining (MMEJ), a process that has been well studied in eukaryotes, and more recently in *Archaea* [48], [49]. However, we think that the observed cases of repeat-mediated deletion in the present study (and in *Halobacterium*) have occurred independently of double-strand breaks, as these would be expected to occur at random positions within a genome if generated by radiation or chemical radicals. It is extremely unlikely that the 4 observed core deletions in the small mobile element HqIRS46 resulted from 4 independent double-strand breaks that, by chance, have all occurred within this repeat. Also, it is quite unlikely that double-strand breaks in *Halobacterium* have – by chance – occurred between the prominent, closely-spaced repeats which occur in the copper-binding domain of halocyanin and in the rRNA promoter region.

The core sequences removed after repeat-mediated deletion events show major size variations, with documented examples ranging from 10 bp to 34 kb (Table S6). Even shorter core deletions have been observed but the resulting events are below our length cut-off of 20 bp (for the sum of core and repeat length). In several cases, the strain not affected by the deletion shows slight sequence variation in the “direct repeat”. It can be assumed that the two copies have been mutated to identity in the other strain prior to the deletion event. Besides the deletion in HqIRS46, there are only four repeat-mediated deletions where the direct repeats are longer than 20 bp (22, 43, 139, 734 bp, respectively). In two cases (43 bp and 22 bp), this is a partial tRNA duplication related to prophage integration (see below).

We propose that repeat-mediated deletions represent an, as yet, undescribed process that contributes to genetic variability. The significance of the present study is that these events have been so clearly documented in natural isolates rather than in experimentally mutagenised systems. The large number of events must reflect a process that is more common than anticipated. The observed similarity between repeat-mediated deletions described here and the MMEJ mechanism of double-strand break repair may indicate that MMEJ may make use of components that are involved in the repeat-mediated deletion process.

It should be emphasized that one prominent source for direct repeats are target duplications associated with transposon insertion. The described repeat-mediated deletion would be an efficient mechanism for trace-free removal of integrated transposons and thus may represent a defence system against such selfish DNA elements. In this context, the high reversion rate of transposon-triggered mutations in *Halobacterium* may be significant [50]. Upon exit from a donor site, a transposon may leave a double-strand break (which unless repaired would lead to loss of the chromosome) or leave the target duplication as a footprint. In the latter case, most *Halobacterium* transposons would introduce a frame-shift, as target duplications of 5, 8, and 10 bp are most common. Thus, regain of function would be unlikely. The high reversion rate of transposon-triggered mutations can be nicely explained by the proposed mechanism of repeat-mediated deletions.

### Cas/CRISPR systems

**Figure 7:**
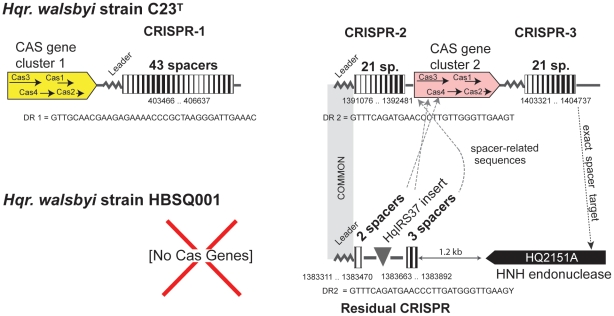
CRISPR systems of *Hqr. walsbyi* strains C23^T^ and HBSQ001. Three CRISPR loci are present in C23^T^, associated with two separate groups of Cas genes (coloured yellow and pink). No Cas genes, and only one, residual CRISPR with 5 spacers, are found in HBSQ001. The DR (direct repeat) sequences are shown beneath the CRISPRs. Shading between the leader sequence of CRISPR-2 of C23^T^ and CRISPR-1 of HBSQ001 indicate that they are nearly identical in sequence. The relative position and orientation of ORF HB2151A is shown along with an arrow indicating where it matches (exactly) a spacer sequence in CRISPR-3 of C23^T^. Similarly, the spacer sequences in HBSQ001 that are closely similar to sequences in C23^T^ are indicated by arrows, labeled spacer-related sequences, which point to their matching locations in the C23^T^ genome. Nucleotide positions are given beneath the CRISPRs and details of the matching spacer sequences are given in Table S7.

In prokaryotes, Cas/CRISPR systems provide a sequence-specific defense barrier against incoming foreign DNA such as viruses or plasmids, and the genes involved often display a high rate of change [19]. The two *Haloquadratum* strains differ radically in their Cas/CRISPR systems, as summarized in Figure 7. Strain C23^T^ carries two complete sets of Cas genes, one preceding the crispr-1 locus, and a second, distinct set located between the flanking crispr-2 and crispr-3 loci. The DR sequence found in crispr-1 differs from that present in the other two CRISPR loci, consistent with their distinct Cas genes. There are 85 spacers spread across the three loci but crispr-1 has duplicates of two spacers, and crisprs-2 and -3 share an identical spacer, making a total of 82 unique spacers. Strain HBSQ001 has no Cas genes and only 5 complete spacers. A MITE (HqIRS37) has inserted within the spacer between the third and fourth copy of the DR (counting from the leader sequence end). The residual HBSQ001 crispr locus is closely related to crispr-2 of C23^T^ as they share the same leader and DR sequences (labeled DR2 in Figure 7), but the lack of Cas genes in HBSQ001 means that the CRISPR system in that strain is non-functional.

The first CRISPR locus in C23^T^ has no homologue in HBSQ001, as it is located on a long deletion-coupled insertion between the two strains (25.4 kb *vs* 6.6 kb), representing one of the variable regions (DV3, Table 3; #88 of Table S5). The segment carrying crispr-2 and -3 is also part of a long deletion-coupled insertion between the strains (15.5 kb *vs*. 5.2 kb, #294), one of 21 strain-specific sequences that constitute hypervariable region DV6 (genomic island GI 2).

**Table 3:** Prominent strain-specific chromosomal regions of strains C23^T^ and HBSQ001.

DV^a^	GI^b^	Region^c^ #	Pos. C23^T^ (nt)	Pos. HBSQ001 (nt)	%G+C (C23^T^/HBSQ001)	Description
DV1		#24	108448 – 129921	104716 – 130155	57.7/57.9	the *hmuI* gene excluding the N-terminal and C-terminal regions which are common to both strains.
DV2	GI 1	#50	260966 – 269209	257293 – 301070 (257397–302834)	46.4/48.6	a 43.7 kb *vs* 8.2 kb deletion-coupled insertion (DCI); codes for S-layer glycoprotein and other cell-surface glycoproteins.
DV3		#88	392540 – 417971	421964 – 428610	48.5/47.8	a 25.4 kb *vs* 6.6 kb DCI^d^; contains a CRISPR in C23^T^ and a transcription unit coding for a probable cell surface structure in HBSQ001.
DV4		#120	560071 – 588686	-	47.3/-	28.6 kb deletion in HBSQ001.
DV5		#162	746237 – 757917	727486 – 738373	49.8/46.5	11.6 kb *vs* 10.8 kb DCI
DV6	GI 2	#264–#314	1268551 –1472326	1269154 – 1454284 (1272280–1457646)	50.0/50.0	Hypervariable region of ∼180 kb in which a total of 21 independent insertion/deletion events were detected. Includes DCI #292 which covers the crispr-2 and crispr-3 loci of C23^T^.
DV7		#370	1615793–1635538	-	44.5/-	prophage integration in C23^T^
DV8		#388	1695969–1730200	-	45.0/-	34 kb repeat-triggered deletion of 34 kb in HBSQ001
DV9		#480	2046794–2069450	1976050–1989452	50.0/47.6	22.6 kb vs 13.4 kb DCI
DV10		#578	2411939–2454921	2275947–2275978	48.6/-	42.9 kb insertion associated with loss of 32 bp. The C23^T^ sequence contains many transposons and a phage integrase, and is possibly an integrative plasmid or a defective prophage. Four of the transposons from DV10 appear to have spread to other genome regions (i.e. ‘outbreaks’).
DV11	GI 3	#658	-	2602626–2655217 (2602766–2661746)	-/51.2	a 52 kb strain-specific insertion in HBSQ001; targets a tRNA and contains an adjacent phage integrase; it is uncertain whether this is an integrative plasmid or a prophage
DV12	GI 4	#682–#704	2952488–3024116	2798883–3008134 (2799525–3012525)	50.0/49.4	two major strain specific insertions over this region, one of which is over 100 kb in length

a **DV**, major divergent regions between the strains are numbered 1–12 and the corresponding positions in both strains given.

b **GI**, genomic islands, as assigned by [13]. Positions are according to the strain comparison analysis. In the cases of assigned genomic islands, the positions for HBSQ001 from [13] are also included in parentheses.

c Region # refers to the numbered common and strain-strain specific sequences listed in Table S5.

d **DCI**, deletion-coupled insertion.

A summary of the significant sequence matches between CRISPR spacer sequences and haloarchaeal or halovirus sequences is given in Table S7. None of the CRISPR spacers exactly matched known haloviruses or haloarchaeal plasmids, but a less stringent search against the PL6 plasmids revealed one C23^T^ spacer that gave an 80% match (29/36 nt) to a sequence within ORF Hqrw_6004 of plasmid PL6A (crispr-2, number 17, Table S7), and the predicted amino acid sequence of this spacer and the corresponding section of the PL6A ORF are nearly identical (11/12 aa). This is not surprising given the widespread carriage of PL6-like plasmids in *Haloquadratum*. Twelve C23^T^ spacers were found to closely match metavirome (virus metagenome) sequences from the Santa Pola saltern, Spain [51]. Four of these spacers perfectly matched metavirome target sequences. On the other hand, 4 of the metavirome contigs were targeted by more than one spacer, suggesting they belong to viruses that frequently interact with *Haloquadratum*. The close sequence relationships found between C23^T^ CRISPR spacer sequences and the Spanish saltern metavirome provide persuasive evidence that the dominant virus types detected in the saltern from which HBSQ001 was isolated are also present in Australia.

Although no spacer sequences were shared by the two strains, four spacers matched sequences adjacent to the CRISPRs of the other strain (allowing for 1–2 mutations; Figure 7, Table S7). These spacer sequences target two Cas genes and a predicted endonuclease. Three spacers of HBSQ001 closely match sites within the block of Cas genes found between the crispr-2 and crispr-3 loci of C23^T^. One of these matching sequences was found after reconstructing the ancestral HBSQ001 spacer by removing the inserted HqIRS37 MITE (Table S7). On the other hand, one spacer of C23^T^ (crispr3/spacer20) exactly matches a 35 nt sequence in HBSQ001 that is found within an ORF (HQ2151) located just 1.2 kb away from the CRISPR remnant of that strain. The HQ2151 protein is predicted to be a HNH restriction endonuclease (COG3440), genes for which are often carried by bacteriophages, haloviruses [52] and plasmids. Spacer sequences matching integrated viruses or plasmids in other strains of the same species have been previously reported, eg. in *Sulfolobus islandicus* [20], consistent with the immunity function of CRISPRs.

It can be assumed that the crispr3/spacer20 sequence in C23^T^ was collected precisely to defend against incoming mobile elements carrying the HQ2151A endonuclease, so the presence of this gene in the Spanish isolate probably represents a successful invasion by such an element despite the CRISPR protection. If the ancestor of the Spanish strain also carried a spacer against this endonuclease, then its integration into the chromosome would have triggered a deletion of at least the cognate spacer, and possibly more, as has been observed in other *Archaea* [53], [54] It seems likely that nearly the complete Cas/CRISPR locus has been deleted in HBSQ001 by such an event, leaving only the observed remnant. Deletions within CRISPRs [55] are well documented, as is the loss of entire sets of Cas genes [56]. It is not clear why the inter-species spacer targets are close to CRISPRs but it may relate to the common occurrence of Cas/CRISPR genes on plasmids, which would be sensed as foreign DNA when transferred to new strains. This is consistent with phylogenetic studies in Bacteria showing that Cas/CRISPR systems appear to spread by lateral gene transfer [57], and indeed the evidence from tetra-nucleotide composition suggests that both CRISPR loci are contained on regions of foreign DNA. It is likely then that plasmid transfers between *Hqr. walsbyi* strains occur at an appreciable rate, although the mechanism(s) involved have yet to be experimentally determined. Overall, the spacer/target sequence relationships observed in the CRISPRs of the two strains, and between the Spanish saltern virome and spacers of C23^T^, show that the two strains are exposed to incursions by closely related organisms, including viruses and plasmids. This evidence points to a rapid, global dispersal system that links the salterns in Australia and Spain, as if they were a global pond (discussed later).

### Halomucins, cell surface proteins, and bacteriorhodopsins

Strain C23^T^ carries an orthologue of halomucin, HmuI, an extremely long protein first described for HBSQ001 [7]. The HBSQ001 protein is predicted to be 9,159 aa (27,477 bp) while that of C23^T^ is 7,837 aa. HmuI is secreted, and surrounds the cells as a cloud of protein as shown by a specific antibody stain (results not shown). It is a highly glycosylated (sialylated) protein, and could play a role in protecting the cell against conditions of desiccation or extremely low water activity [7]. Although the HmuI of C23^T^ is considerably shorter, the two halomucins retain blocks of high sequence conservation with more than 90% sequence identity, especially in the N-terminal and C-terminal regions. They possess the same major features, an N-terminal Sec-pathway signal sequence, stretches of multiple direct repeats that span much of the protein, and a serine/glycine rich region near the C-terminus. However, the C-type lectin-like (CTLD) domains carried by the HBSQ001 protein are absent in the C23^T^ HmuI. Comparison at the DNA sequence level indicates that the *hmuI* genes must have diverged *in situ* from a common ancestral gene rather than by lateral gene transfer events as both are embedded within a common genomic region that extends into each gene for 119 codons at the N-terminus, and for 559 codons at the C-terminus. Between the flanking core sequences the genes vary in sequence, and this region is designated as divergent region (DV1, #24). The repetitive domain structure of halomucin is likely to have promoted length changes by standard homologous recombination or repeat-mediated deletion, and two independent deletions can be discerned; one of 2.4 kb (HBSQ001 codons 283-1075) which removed the pair of CTLD domains, and one of 1.3 kb (HBSQ001 codons 5078-5538). There are also several other, smaller insertions and deletions. When the HmuI of HBSQ001 was first described in 2006 there were no prokaryotic homologues in the sequence databases but current BLASTP searches of the Genbank database show matches to large, secreted proteins from a number of recently sequenced *Archaea* (including haloarchaea) and *Bacteria.* These proteins, often annotated as large exoproteins, GLUG-domain proteins, or filamentous haemagglutinins, are considerably shorter in length than even the C23^T^ HmuI, with most being between 1,000–2000 aa. The closest sequence was that of Ig-like domain-containing protein Swol_1078 of *Syntrophomonas wolfei* (E = 10^−44^). Two groups of matching sequences were observed; those similar to the HBSQ001-specific CTLD domain containing region (aa 500 – 1,060), and the great majority that matched to the repeats (approx. aa 1,500–5,500 of C23^T^ HmuI). The similar repeat domains of the latter group of proteins probably reflect a common structural characteristic, such a filamentous shape, and it is possible that HmuI provides an attachment function.

Two additional proteins with characteristics similar to halomucin (very large and probably secreted glycoproteins) have been reported for HBSQ001: Hmu2 (2885 residues) and Hmu3 (2079 residues). As these are encoded on strain-specific sequences (*hmu2* on #50, *hmu3* on #692), similar proteins are not present in C23^T^.

The cell walls of most haloarchaea, excluding halococci, typically comprise a single protein layer, the surface or S-layer that lies directly outside the cell membrane. Experimental evidence from a few haloarchaeal species indicates that the S-layer is composed of only one protein that is usually heavily glycosylated, and forms an interlocking two-dimensional protein sheet attached to the cell membrane [58], [59]. Haloarchaeal genome sequences often have multiple genes annotated as cell surface (glyco)proteins, based largely on the presence of secretion and glycosylation signals. Strain C23^T^ has twelve such ORFs but only one, Hqrw_1237 (822 aa), is closely related to known S-layer glycoproteins. The highest similarity is to the S-layer protein of *Har. japonica* (Q9C4B4, 64% aa identity). A second, much smaller ORF, Hqrw_1408 (222 aa), present in both strains, shows about 40% aa identity to the C-terminal 140 residues of known S-layer proteins. The remaining annotated surface proteins share no significant similarity to S-layer proteins. Mass spectrometry analysis of membrane preparations of C23^T^ revealed the presence of Hqrw_1237, as expected, while Hqrw_1408 was not detected. In addition, three of the other predicted surface proteins were unambiguously identified; Hqrw_1240, Hqrw_1641 and Hqrw_2184. The functions of the latter proteins are unknown.

The probable S-layer glycoprotein genes (*csg*) of C23^T^ (Hqrw_1237) and of HBSQ001 (HQ1207A) are located on a long deletion-coupled insertion (43.7 kb *vs* 8.2 kb, #50). In the latter strain, this had previously been reported to be a hypervariable region, and denoted as genomic island GI 1 [13]. The S-layer protein of C23^T^ aligns to homologues from other halophiles beginning from its N-terminus, while the orthologue of HBSQ001 appears to have an extended N-terminal region. Only from aa 193 onwards does it show significant sequence identity (55%) to the C23^T^ homologue Hqrw_1237 (starting at pos. 55), and to the S-layer glycoproteins from other halophiles. However, the N-terminal 192 aa show only very distant homology (if any) to proteins from C23^T^ or other halophiles. Nevertheless, the protein has a signal sequence and is one of the major membrane proteins in HBSQ001 (Fusetti & Pohlmann, unpublished results). Thus, it is likely to be the S-layer glycoprotein of the Spanish isolate.

The similarities in the C-terminal region between the probable S-layer glycoproteins from the two strains could have been caused by a genome rearrangement within the coding region, but this is not the case, as the level of nucleotide sequence conservation (55%) is drastically lower than the average similarity of other orthologue pairs (97%). The genomic breakpoint has been unambiguously positioned and is located outside of the *csg* gene. Cuadros-Orellana *et. al* [13] suggested that some of the variability of this region (GI1) could be due to recombination with related sequences elsewhere on the chromosome. However, this seems less plausible in C23^T^ as there is little intragenomic similarity. Thus, in contrast to the situation described for the halomucin gene, the *csg* gene has most likely been acquired by lateral gene transfer in the Spanish isolate. A third form of the cell surface glycoprotein was found on fosmid eHwalsbyi 559 also within the same synteneic gene context [13].

The HBSQ001 specific 43.7 kb DV2 sequence codes not only for a distinct *csg* and the additional *hmu2* gene, but also for 5 additional proteins that are probably secreted and glycosylated. These differences are likely to be responsible for the three-layered cell wall structure observed in HBSQ001 compared to the two-layered surface of C23^T^ [8]. The combination of a long *ca*. 150 aa N-terminal extension on the S-layer protein of HBSQ001, and the additional cell surface glycoproteins in this strain, are probably important in altering the outer cell surface to evade viral infection, and may have helped HBSQ001 survive such attacks despite the absence of a functional CRISPR system. There is also a potentially significant difference in the glycosylation pattern of surface and secreted proteins between the two isolates. Sialic acid synthesis genes are present in HBSQ001 but absent in C23^T^, as they occur on a deletion-coupled insertion (#690, within DV12).

Both strains carry two different bacterio-opsin-like genes, annotated as bop1 and bop2, along with a single halo-opsin gene (hop). To check for the expression of these and other membrane proteins, cell membranes of the C23^T^ strain were purified using the protocol for isolating purple-membranes of *Halobacterium salinarum* [60], and a red-membrane band was observed at the same position on sucrose gradients as purple membranes from *Halobacterium salinarum*. SDS-PAGE revealed a single, prominent Coomassie stained band of identical apparent MW to Bop of *Hbt. salinarum* (data not shown), and MS analysis of the excised band confirmed it to be BopI (Table S3). In the previous study of HBSQ001, BopI was also stated to be present in the cell membrane [7]. Minor protein components of the purified membranes of the C23^T^ strain were also detected, among which were BopII, Hop and Brp-like protein (carotenase) (Table S3). Recently, BopII of *Hqr. walsbyi* has been found not to pump protons, and its actual function in the cell is yet to be determined [61].

### Prophages

The genomes of both strains were examined for prophages using a number of methods including searches for insertions into tRNA genes, similarity to known halovirus (or prophage) genomes, and to highly conserved virus genes (e.g. terminase).

As mentioned above, genomic island 3 (GI 3, DV11, #658) may represent a strain specific prophage in HBSQ001. Another likely prophage of C23^T^ occurs at nt 1615793–1635538, where a 19.7 kb virus-like region has integrated into the tRNA-Ala gene. This is a strain specific insertion (#370, DV7) with an average %G+C content of 44.5, about 3 percentage points lower than that of the entire chromosome, and an altered tetra-nucleotide composition. A phage integrase is adjacent to the right end, which is marked by a 22 nt duplication of the tRNA. The ORFs between are typical of bacteriophages, including DNA polymerase, restriction/modification enzymes and a cluster of several ORFs that overlap at start/stop codons and are likely to be transcribed as a single unit. The DNA polymerase (Hqrw_2609), which is adjacent to the left end of the tRNA gene, is most similar to that of halovirus HF1, but is interrupted by a frame-shift, so this prophage is either defective or relies on the host enzyme for replication.

Common to both strains is a partial duplication of the tRNA-Thr1 gene that occurs 6.7 kb further downstream (nt 103,049–103,105 in C23^T^). The region between contains genes for hypothetical and conserved hypothetical proteins, along with many pseudogenes, some being *cdc6* homologues. The partial tRNA-Thr1 copy is imperfect, with 5 mismatches over 57 nt, and is missing 15 nt at the 5′ end. There are no integrase genes nearby. A similarly truncated tRNA-Thr gene is also carried by haloviruses HF1 and HF2, but these occur nearby a predicted integrase gene, presumably to allow host integration [52]. It is likely that this 6.7 kb region of *Haloquadratum* is a remnant of either a prophage or an integrative plasmid.

As discussed above, plasmid PL6 may represent a prophage in C23^T^. Whether the larger plasmid, PL100, is a prophage or a plasmid is difficult to predict as it shares no significant similarity to known haloviruses and has a high proportion of hypothetical or conserved hypothetical ORFs. While ORF Hqrw_5003 shows almost perfect similarity to a metavirome sequence derived from the Santa Pola saltern in Spain (ADE29299), and several other ORFs show significant similarity to halovirus or bacteriophage ORFs, many of these are similar in function to genes also typically carried by plasmids, such as endonuclease, helicase, integrase, and Cdc6 homologues. The presence of a Cdc6 homologue cannot be taken as evidence that PL100 is simply a plasmid, as halovirus BJ-1 carries such a gene [62].

### Divergent regions, including genomic islands (GI) of strain HBSQ001

Four hypervariable regions (genomic islands, GI 1–4) of HBSQ001 were described by Cuadros-Orellana *et al.* [13] (Fig 5). These show high variability between the HBSQ001 genome and environmental sequences recovered from the saltern from which this strain was isolated (i.e. autochthonous DNA). Since GIs 1–4 largely correspond to divergent regions (DV) observed in the current study (described above), only relevant information that has not already described will be given here. Genomic Island 1 corresponds to DV2 (see above). Genomic Island 2 is 185 kbp in length (nt 1269154 – 1454284, #264 – #314 of Table S5) and corresponds to a similarly sized region, DV6, in C23^T^ (203 kb; nt 1268551 – 1472326). The latter region harbors many IS elements, phage-associated, and metabolic genes. Unlike most other GIs and DVs, which display a single large indel between the two strains, GI 2 shows 21 independent indels or deletion-coupled insertions, several of which exceed 10 kb. These strain-specific sequences are interspersed among core sequences ranging from as little as 43 bp to 12 kb. Commonly, the indels are separated by not more than 5 kb (Table S5). The whole region has an increased G+C content (50.0%), an altered tetra-nucleotide frequency, is devoid of tRNAs and ribosomal protein genes, and is located mid-way between *ori1* and *ori2*/*ori3.* GI 2 (DV6) is particularly abundant in non-transposase related pseudogenes (Figure 1). The clustering of numerous distinct indels/replacements in a probable replication termination region is curious, but appears similar to observations in strains of *Sulfolobus islandicus* recovered from widely spaced sites (see Figure 3 of [33]).

GI 2 (DV6) begins abruptly in HBSQ001 just after nt 1269163 (strain-specific region #264) where a large deletion has removed most of ORF HQ2048A. Comparison with C23^T^ shows this is a deletion because the corresponding C23^T^ ORF, Hqrw_2212, remains intact. The ends of the undeleted region in C23^T^ have a direct repeat, ACATCATTCT, while in HBSQ001 there is only one copy directly at the deletion border. This is one of the cases of a repeat-mediated deletion, which has eliminated just over 16 kb from the HBSQ001 genome. Fortuitously, among the autochthonous DNA sequences recovered from the Santa Pola saltern in Spain by [13], was a 15 kb fosmid clone sequence (EF583981) that shared a similar %G+C as *Hqr. walsbyi* but did not match any sequence in HBSQ001. In the current study this sequence was found to be 99% identical to C23^T^ across its entire length, and mapped within this 16 kb deletion in GI 2 (nt 1268551–1284680). This example further supports the tight relationship between *Hqr. walsbyi* populations present at the two distinct global sites.

A significant strain-specific difference found within GI 2 (DV6) is the CRISPR array and associated Cas genes of C23^T^ (see above). In this case C23^T^ has 15.5 kb relative to 5.2 kb in HBSQ001 (#294). The comparative sequence data is consistent with HBSQ001 suffering a deletion relative to C23^T^, as the leader and first DR are contiguous with the shared sequence between the two strains (Figure 7), but after this HBSQ001 has only a few remnant spacers left while C23^T^ retains a full set of spacers as well as Cas genes.

#### Divergent region DV3

The genes carried by the two strains in this deletion-coupled insertion (25.4 kb *vs* 6.6 kb, #88) are quite different, and the ancestral strain can be identified by ORF HQ1332A, which spans the replacement border in HBSQ001 and appears to be complete, while the corresponding ORF in C23^T^ is truncated, indicating that the former strain has the original sequence. DV3 in C23^T^ carries not only the Cas/CRISPR system of the crispr-1 locus, but also genes typically found in mobile DNA elements, such as phages or plasmids (integrase, restriction and modification enzymes, transposons). In HBSQ001, DV3 has genes that are probably all involved in the synthesis of a cell surface structure. Type IV pilus biogenesis proteins (*pilB*/*pilC* homologues) are followed by 5 genes, of which two (HQ1332A, HQ1334A) have a predicted class III signal peptidase (preflagellin peptidase) cleavage site according to PilFind [63]. Class III signal peptides are believed to be particularly important for the biogenesis of archaeal cell surface appendages, and proteins containing class III signal peptides frequently occur in operons together with pilus/flagella assembly systems (*pilBC* or *flaIJ* homologues) [64]. Genes highly homologous to the probable cell surface components from DV3 of HBSQ001 are found in other halophilic archaea, and it is likely that this structure is used by many other halophiles.

#### Genomic Island 3: a tRNA insertion (DV11)

This represents a 52 kb long (nt 2602626 – 2655217) strain-specific insertion in HBSQ001 (#658). Cuadros-Orellana *et al*. [13] suggested that GI 3 may represent ‘the remnant of a lysogenic phage’. By comparison with C23^T^, this insertion begins at a tRNA-His gene (HQt41, nt 2602504) and ends with a 43 nt repeat of the 3′ end of the same tRNA (at nt 2655218–2655260). A phage integrase gene immediately downstream of the left-end tRNA is closely related to the integrase gene of halovirus HF2 (NP_542565; 35% aa identity), which is itself adjacent to a truncated tRNA gene (HF2t004) in the HF2 genome [65]. More closely related homologues of this integrase occur in the genomes of *Hfx. volcanii* (HVO_2815A), *Hgm. borinquense* (ZP_03999761) and *Nab. magadii* (Nmag_0465). In all cases these are located next to a complete tRNA gene (all are tRNA-Glu), while a truncated sequence of the same tRNA is found a further 5–48 kb downstream. This arrangement is typical of integrative prophages [66] and the insertion carries several possible phage-associated genes (an additional integrase, helicases and restriction-systems). It also includes many hypothetical ORFs, Cdc6 and Cdc48 homologues (HQ3268A, HQ3269A, HQ3297A), as well as type IV secretory pathway related genes (HQ3289A, HQ3291A). The comparative data indicate it is able to target the tRNA-His gene via a partial copy of the same sequence, and probably has the capacity to excise (via the adjacent integrase), recircularize, and replicate via the Cdc6 genes. There is no recognisable phage terminase gene [66] and without more information it is uncertain whether it is an integrative plasmid or a prophage (defective or functional).

#### Genomic Island 4 (DV12)

At 209 kb (nt 2798883-3008134, #682-#704) this was the largest GI detected by Cuadros-Orellana *et al*. [13]. HBSQ001 shows two major strain-specific insertions over this region, one of which is over 100 kb in length. Looking more closely, these were found to be coupled to deletions, the first (#692) being 0.4 kb (C23^T^) *vs* 105 kb (HBSQ001; nt 2841233–2945868), and the second (#698) being 5.4 kb (C23^T^) *vs* 33.6 kb (HBSQ001; 2949791–2983415) (Figure 1, Figure 5, and Table S5). The region of C23^T^ from nt 2993444 to 3008735 (ORFs Hqrw_3995 to Hqrw_4025), which spans both of the latter deletion-coupled insertions, shows a prominent deviation in tetramer frequency and higher than average %G+C, indicative of foreign DNA. The origin of the latter element is unclear as most ORFs are either hypothetical or conserved hypothetical, and many have degenerated to pseudogenes, including a transposase at the left end (Hqrw_3995) that may have originally facilitated its integration. The general characteristics of this element are similar to another region of C23^T^, Hqrw_2042 to Hqrw_2066, which also shows an increased %G+C, divergent tetramer composition, begins with a phage integrase pseudogene, and contains largely hypothetical/conserved hypothetical ORFs, many of which have degenerated to pseudogenes. However, this region is conserved between the two strains.

### Mobile genetic elements (MGEs)

Mobile, integrative genetic elements, because of their repetitive and propagative nature, are significant drivers of cellular evolution, inactivating genes and generating multiple copies that can lead to deletions and other genomic rearrangements [67]. Knowledge of these elements in a genome can help explain its structure, evolution and propensity for change, but identifying them can be difficult, particularly if they are novel, short, or show some variability in sequence. The unexpectedly close similarity of the two *Haloquadratum* strains provided an extraordinary advantage in detecting such elements. A surprising finding from these comprehensive searches is that despite both strains carrying numerous mobile genetic elements, their shared sequences have remained synteneic. Rather, there is a strong tendency for MGEs to target other MGEs.

Both strains carry large numbers of MGEs: a total of 528 for C23^T^ and 536 for HBSQ001. These are of various types, including canonical transposons (13 families), IS605-type transposons (29 families) and three categories of transposase-free mobile repeats: MITEs (miniature inverted-repeat transposable elements, 5 families), PATEs (palindrome-associated transposable elements, 6 families), and SMRs (small mobile repeats, 5 families). Since MITEs lack a transposase, their mobilisation relies on an appropriate transposase provided *in trans*, which also determines the type of target duplication (if any). A MITE cannot be considered a degenerate version of the transposon supplying the transposase, as their sequences are unrelated except for the few terminal bases of the inverted terminal repeat. SMRs also contain ITRs but do not cause target duplications and, unlike MITEs, their ITRs show no sequence similarity to any of the transposons. Currently, their mode of mobilization is enigmatic. We observed a strong targetting preference for several of the SMR families (see below). PATEs differ from MITEs and SMRs in being devoid of ITRs, but they contain near-terminal palindromic sequences. During the analyses described below, these were found to be related to those of IS605-type transposons. Indeed, some PATEs appear to have originated from IS605-type transposon by repeat-mediated deletion of the core sequence, and so represent degenerate versions with only the fused terminal sequences.

Transposase-free MGEs (MITEs, PATEs, and SMRs) are far more numerous than transposons (Table 4) and are responsible for the majority of the strain differences caused by MGEs (Figure 6). The distribution of these elements between the two strains of *Haloquadratum* is similar except for two families of SMRs that are only present in C23^T^ (see below). MGEs appear to be relatively evenly distributed around the C23^T^ chromosome (data not shown) but the large plasmid, PL100, is completely free of SMRs, possibly reflecting a recent acquisition.

**Table 4:** General properties of the six categories of *Haloquadratum* MGEs.

Category^a^	Families	Types	Total/SSEI (C23^T^)	Total/SSEI (HBSQ001)	Characteristics
IS605-type Transposons (TP-A)	29	44	99/4(+3)	118/10(+7)	Do not have terminal inverted repeats. Have palindromes close to their termini. Do not cause TDs. Contain an entire transposase gene.
Canonical transposons (TP-B)	13	47	73/28	41/1	Have terminal inverted repeats. Often cause target duplications. Contain an entire transposase gene.
Miniature inverted-repeat transposable elements (MITEs)	5	8	55/14	55/12	Have terminal inverted repeats that are related to known transposons. Often cause target duplications. Too short to code for a transposase
Palindrome-associated transposable elements (PATEs)	6	39	177/10(+4)	194/22(+4)	Do not have terminal inverted terminal repeats. Have palindromes close to their termini. Do not cause TDs. Too short to code for a transposase.
A-type Small Mobile Repeats (SMR-A)	3	3	109/14	128/16	Have terminal inverted repeats but these are not related to known transposons. Do not cause TDs. Too short to code for a transposase. Frequently integrated into a TP-A or PATE.
B-type Small Mobile Repeats (SMR-B)	2	2	15/5	0	Have terminal inverted repeats but these are not related to known transposons. Do not cause TDs. Too short to code for a transposase.
Total	58	143	528	536	

a Canonical transposons (TP-B), and A-/B-type Small Mobile Repeats (SMR) show a high sequence conservation between elements classified into the same type (commonly >95% sequence identity). PATEs and IS605-type transposons are much more divergent, except for a few types of homogeneous PATEs. MGE types classified into the same family show sequence homology of the element itself or of the encoded transposase. The total number of elements and the number of SSEIs (total/SSEI) is given for strains C23^T^ and HBSQ001. For TP-A and PATE, a distinction is made between SSEIs of complete elements (first value) and SSEIs of terminal-only elements that have suffered core deletions (second value in parenthesis, prefixed with a plus sign). Terminal-only TP-As have lost their transposase genes and so have degenerated to PATEs.

In comparing MGEs between the two strains, the following categories were recognized: (a) common elements, which occur at corresponding genome positions, (b) strain-specific element insertions (SSEIs), which represent one type of strain-specific elements, and where the adjacent sequence is contiguous in one strain and interrupted by the mobile element in the other, (c) elements which occur within larger, strain-specific sequences (indels), and so are likely to have either been carried into the cell as part of a segment of foreign DNA, or have inserted into such a segment after its integration into the chromosome. In several cases, indels start and/or terminate with an MGE. This may imply that MGEs can trigger deletion events that may also remove neighbouring sequences.

The mobility of MGEs was inferred from the presence of strain-specific insertions (SSEIs, case b), and although these could possibly also result from the removal of a hypothetical corresponding element in the other strain, e.g. by repeat-mediated deletions via the flanking target site duplications, we believe most SSEIs represent insertions. On this basis, the most active MGEs in C23^T^ are the SMR HqIRS55 (10 SSEIs), the C23^T^-specific transposase ISHwa4 (8 SSEIs), and the PATE HqIRS56 (7 SSEIs).

The key features of all *Haloquadratum* MGEs are summarized in Table S8, in which complete transposons are referred to by their ISFinder database numbers [68], prefixed with ISHwa, while the other elements are assigned numbers prefixed with HqIRS (for ***H*** *alo* ***q*** *uadratum* **I**S-**R**elated **S**equences).

### Transposons and MITEs

Twelve of the 13 distinct transposon families detected in the two strains have been previously described in *Halobacterium* [21] or other haloarchaea, but the remaining one, ISHwa2, is related to Tn5, a transposon widely distributed in Bacteria. Although ISHwa2 occurs multiple times in both strains it is not found in any of the other 78 completely sequenced Archaea, except for a small remnant in *Natronomonas pharaonis*. This distribution is indicative of a lateral gene transfer event from a bacterial representative, and the transposase of ISHwa2 most closely matches homologues in *Microcoleus chthonoplastes*, a cyanobacterial species known to inhabit hypersaline environments.

Transposon mobility is likely in at least 10 of the families as examples of SSEIs were observed but they were not equally active: seven of the 10 transposon families show SSEIs exclusively in strain C23^T^, while the remaining 3 transposon families show SSEIs in both strains.

In some transposon families, the pattern of SSEIs and their distribution within and between strains indicates that the transposon was carried into the cell via an integration event of foreign DNA providing a source of infection for the rest of the genome. As detailed in Text S1, this is exemplified by a 43 kb C23^T^-specific sequence (DV10, #578), which carries a total of 10 transposons (or fragments thereof), several glycosyltransferase genes and one phage integrase homolog. It is likely that DV10 is responsible for the ‘outbreak’ of four transposons (ISHwa4, ISHwa8, ISHwa12, and ISHwa13). Although the DV10-specific copies of ISHwa8 and ISHwa12 are broken, this could have occurred after their spread to other genome locations (see Text S1).

MITEs depend on a suitable transposase supplied *in trans* by a regular transposon, with which they share a similar inverted terminal repeat sequence as well as the length of an eventual target duplication [69]. Four of the five distinct families of MITEs detected in *Hqr. walsbyi* are non-coding and shorter than 500 bp, and the most frequent of these have been previously described [69]. The ISH2-type element HqIRS32 is the longest MITE (ca. 520 bp) and is the only example containing an ORF likely to code for a protein (but not a transposase). There is evidence from *Halobacterium* that this ORF in ISH2 is expressed [70]. Homologous short ORFs were detected in (HqIRS33 on DV10, HqIRS34).

The mobility of some MITEs, as reflected by SSEIs, was curious, as no functional copy of the appropriate transposase was found elsewhere in the genome. For example, MITE HqIRS39 has an ITR similar to that of transposon HqIRS12 but all copies of the HqIRS12 transposase are disrupted by frame-shifts or in-frame stop codons (see also Text S1).

### Short mobile repeats (SMRs)

Three families of SMRs (HqIRS42, HqIRS43, and HqIRS55, grouped as SMR-A) share a number of common features despite a lack of sequence similarity: they are about 110 bp long, have prominent inverted terminal repeats, do not generate target duplications, are relatively frequent (up to 54 copies in one genome), and seem to have a strong and identical target site preference, namely to target PATEs and IS605-type transposons, inserting within their near-terminal palindromes (see also below). As an example, the relatively frequent PATE HqIRS56 is regularly targeted by SMR-A elements (9 of 19 in C23^T^ and 12 of 23 in HBSQ001).

The other two families of SMRs (HqIRS40 and HqIRS41, grouped as SMR-B) occur only in C23^T^. SMR-Bs are similar to SMR-As in that they target other MGEs but show little targeting preference. HqIRS41 is exceedingly short, being only 80 bp long.

### IS605-type transposons and PATEs

#### IS1341-type transposases are highly expanded and diverse in halophilic archaea

The transposition mechanism of IS605-type transposons has recently been elucidated, and is unusual in using a ssDNA intermediate [71]. IS605-type transposons are characterized by palindromic sequences near both termini, which in some cases have an identical sequence. IS605-type transposon integration depends on a short oligonucleotide (4–6 bp) that functions as a targeting sequence [72] as already shown for ISHp608 from *Helicobacter pylori*. The terminal sequence prior to the near-terminal palindrome is highly variable. In IS*Hp608*, it has been shown that the IS200-type transposase is responsible for mobilization while the IS1341-type “transposase” is an accessory protein that does not function as transposase [72].

ISH1-8 was first detected on halophage phiH1 and corresponds to ISH12 from *Halobacterium*. It is a member of the IS605-type transposons, and codes for two proteins, TnpA (annotated as IS200-type transposase) and TnpB (annotated as IS1341-type transposase). The genes are serially encoded and much closer than initially annotated. Besides ISH12, *Halobacterium* contains a moderate number of homologs of the TnpA (6) and TnpB (16) transposases. All copies of *tnpA* are adjacent to *tnpB* (4 serial configurations, 2 divergent configurations). In the comparison of *Halobacterium salinarum* strains R1 and NRC-1, the mobility of this element was not obvious due to the lack of SSEIs [21].

The family of IS605-type transposons is highly expanded in *Natronomonas*, which carries 38 copies of IS1341-type tranposase (*tnpB*) and 3 copies of IS200-type transposase (*tnpA*), each of the latter being encoded directly adjacent of a *tnpB* gene [73]. However, the other 35 *tnpB* are not linked to a *tnpA,* which seems unusual if they were only accessory proteins. Among the peculiar features of IS605-related transposons are – besides the lack of ITRs – the lack of target duplications and the lack of close sequence similarity between different copies of the transposon. Commonly, transposases from mobilized transposons form large families, members of which share more than 90% sequence identity. However, if the 38 IS1341-type transposases of *Natronomonas* are clustered at the 90% sequence identity level, the largest cluster has only 3 members. There are only 5 additional clusters with two members; all the other IS1341-type transposases remain singlets at a 90% sequence identity level. This may indicate that a large number of distinct transposons have invaded the genome, but each of these is under a stringent copy number control. As IS605-type transposons lack all the characteristic features that facilitate identification of the element boundaries, annotation of the complete transposons is severely hampered.

The family of IS605-type transposons is even further expanded in *Haloquadratum,* which codes for ca. 90 IS1341-type transposons (*tnpB*: C23^T^: 82; HBSQ001: 100) and ca. 16 IS200-type transposons (*tnpA*: C23^T^: 15; HBSQ001: 17). As in *Natronomonas*, IS1341-type transposons are extremely diverse with only very small clusters being formed at a 90% sequence identity level. The largest cluster of HBSQ001 has 3 members and each of these represents an SSEI. However, while the transposons in *Natronomonas* are commonly functional, in *Haloquadratum* they are mostly disrupted by deletions, frame-shifts, or in-frame stop codons (disrupted *tnpA*: *Nmn*., 0 of 3; C23^T^, 15 of 15; HBSQ001, 16 of 17; disrupted *tnpB*: *Nmn*., 6 of 38; C23^T^, 83 of 100; HBSQ001, 77 of 82). The finding that mobilization of IS605-type transposons depends exclusively on *tnpA* [72] is difficult to reconcile with our data on *Haloquadratum,* where SSEIs are observed although all of the IS200-type transposons are pseudogenes in C23^T^. Only a single IS200-type transposase is not disrupted in HBSQ001 and this is encoded adjacent to a disrupted IS1341-type transposase.

#### Homogeneous and heterogeneous PATEs

In the course of the analysis it became evident that the near-terminal palindromic sequences found on PATEs are similar to those of IS605-type transposons. In several cases, a single PATE has expanded in a way that is typical for other transposons, showing full-length homology with more than 90% sequence identity. These were designated homogeneous PATEs, and five types were detected (Table S8). Each of these shows SSEIs that, in combination with the high sequence similarity, facilitated definition of the element boundaries. Homogeneous PATEs show prominent near-terminal palindromes (Table S9) that, for each PATE, are similar in sequence, especially for HqIRS46, which has an exact duplication of 22 bp.

BLASTN analysis of homogeneous PATE sequences resulted in a number of partial hits with either reduced sequence similarity or with hits that were short but highly conserved. These proved to belong to additional but heterogeneous PATEs. Even more hits were obtained upon blast analysis with the newly identified elements. Finally, this iterative approach resulted in the collection of PATE elements to saturation.

Heterogeneity of PATE sequences is especially prominent in the terminal region beyond the near-terminal palindrome (20–50 bp). However, most PATE subclasses are linked to a well-conserved short “terminal oligonucleotide”, and when this could be linked to a near-terminal palindrome in a manual alignment, it was considered to represent the element boundary.

Several individual copies of PATEs seemed to have incomplete near-palindrome sequences and to lack the terminal sequence, but upon further inspection, unrelated repeats (mainly SMR-A) were found at the ends of such incomplete PATEs. Due to the high sequence conservation of homogeneous PATEs, it was possible to detect that the missing terminal sequence occurs on the other side of the SMR-A repeat, consistent with these PATEs being targeted by SMR-A elements.

#### PATEs and IS605-type transposons have similar boundaries

Most of the haloarchaeal IS605-type transposons had been incompletely characterized, with annotations being restricted to the transposase-coding region. Complete element annotations including boundaries were available for only a few examples, mainly based on SSEIs. However, once it became evident that some PATEs have near-terminal palindromes that are similar to those of completely annotated IS605-type transposons, a systematic comparison was performed. Frequently, a segment of 200 bp upstream or downstream of the coding region matched near-terminal palindromes from PATEs. For this analysis, it was advantageous that heterogeneous PATEs had been collected to saturation, which increased the occupancy of the sequence space and allowed the detection of a sufficiently close homolog using BLASTN analysis. In many cases, the well-conserved ”terminal oligonucleotide” was also encountered (directly by blast or upon a manual alignment) and facilitated boundary detection. For the IS605-type transposon ISHp608, it was reported that the highly conserved “terminal oligonucleotide” functions as targeting sequence which is not part of the transposon [71], [72]. For practical reasons, we used the convention of annotating the targeting sequence as part of the element as this greatly facilitated homology-based boundary setting. However, data from PATE SSEIs support the assignment of the well-conserved “terminal oligonucleotide” as a targeting sequence rather than an integral part of the element. Most of the SSEIs are associated with a few “extra bases” in the element-free strain when SSEI boundaries are annotated according to the extended element including the targeting sequence (Table S8).

As a result of the effort to define transposon boundaries, the number of completely annotated IS605-type transposons could be largely expanded. As the majority of IS605-type transposons in *Haloquadratum* code only for pseudogenes, the analysis was first performed for *Natronomonas pharaonis*. For this haloalkaliphilic archaeon, transposon boundaries could be defined for 22 of the 38 IS605-related transposons (submitted to the ISFinder database as ISNph5-ISNph22). The high sequence conservation between IS605-related transposons from *Natronomonas* and of homogeneous PATEs from *Haloquadratum* is remarkable (Table S9, section B). *Haloquadratum* IS605-type transposons with non-disrupted transposases, and for which complete boundaries could be assigned, were also submitted to ISFinder (ISHwa17-ISHwa19, ISHwa21-ISHwa29).

### Core deletions in PATEs and IS605-type transposons occur by repeat-mediated deletion

HqIRS46 is a PATE with a direct repeat of 22 bp, representing the two near-terminal palindromes (Table S9). In four cases, the element is complete in one strain while the core and one copy of the 22-mer have been deleted in the other strain. Core deletions, which lead to a terminal-only version of the element with only 52 bp, have all the characteristics of repeat-mediated deletions (Table S6). However, there are not only examples where the core has been deleted in one of the strains, there are also cases where terminal-only copies of HqIRS46 occur at the same genome position in both strains. In these cases it cannot be decided if the core has been deleted independently in the two strains or if the terminal-only version of the element has been mobilized.

An equivalent core deletion occurs for IS605-type transposon ISHwa23, which codes for an IS1341-type transposase (TnpB). The deleted region is flanked by a 19 bp direct repeat which, again, resembles the near-terminal palindrome (Table S6). In this case, the element is shortened from a 1500 bp transposon to a 52 bp PATE. As described in the Text S1, this core deletion removes a transposon that must have been mobilized before. There are 6 SSEIs of ISHwa23 in HBSQ001, but in all six cases the terminal-only 52 bp version is found. This may indicate that this extremely short transposon-derived PATE can be mobilized.

Several other of the IS605-type elements were found to exist as terminal-only PATEs. In addition, several elements contain other types of internal deletions that remove most of the transposase genes. In several transposon remnants that carry IS200-type and IS1341-type transposase genes in divergent orientation, large internal regions are deleted so that only short C-terminal remnants of the two transposons persist. One PATE, HqIRS71, shows high sequence similarity to an extended terminal segment of certain IS605-type transposons. This includes part of the C-terminal coding region, but all ORFs on HqIRS71 are disrupted and so HqIRS71 is considered to be non-coding.

In summary, IS605-type transposons frequently suffer deletions in *Haloquadratum*. This is one of the reasons why most of the IS1341-type transposons are pseudogenes in this organism. On several occasions, direct repeats are found adjacent to the deleted region and thus we propose that the mechanism of repeat-mediated deletion provides a defense mechanism against IS605-type transposons and related PATEs in *Haloquadratum*.

#### PATEs and IS605-type transposons are attacked by SMR-A type elements

It has been mentioned that PATEs are targeted by SMR-A elements (HqIRS42, HqIRS43, and HqIRS55). MGE conglomerates, where one MGE is targeted by another MGE, have been frequently described for halophilic archaea, and we were initially not surprised to find SMR-A elements within PATEs. However, it became evident that being targeted by SMR-A elements is another characteristic that is common between PATEs and IS605-type transposons. Most of these targeting events occur outside the transposase ORF, in the 5′ or 3′ untranslated regions, and this only became evident upon precise annotation of complete transposons up to their boundaries.

We systematically counted all cases where an MGE occurs within another MGE and found a total of 192 such targeting events (Table 5). The majority of these (59%, 114 events) are caused by SMR-A elements and more than four-fifths (89%, 170 events) have been suffered by PATEs and IS605-type transposases. Together, more than half (54%, 104 events) of these events represent targeted insertions of a SMR-A element into a PATE or an IS605-type transposon. We consider it likely that this represents another cellular defence mechanism against PATEs and IS605-type transposons. It seems that the biological function of SMR-A elements is to inactivate IS605-type transposons and related PATEs, but their mechanism of mobilization remains enigmatic. While nearly all MITEs (30 of 31) are integrated into PATEs or IS605-type transposons they differ from A-type SMRs in not showing a strong preference for PATEs.

**Table 5:** Targeting of *Haloquadratum* MGEs. This lists the 192 targeting events where one MGE was found inserted into another MGE. Rows indicate how frequently elements of the given category have targeted another element, while columns indicate how frequently an element of the given category has been affected by insertion of another element. More than half of the targeting events are caused by A-type SMRs (114, 59%), while the majority of the affected elements are from PATEs (106, 55%) or from IS605-type transposons (TP-A, 64, 33%). Thus, these related categories of elements suffered 88% of the foreign element insertions.

	PATE	TP-A	TP-B	MITE	SMR-A	SMR-B	Total
**PATE**	6	12	-	-	-	-	18
**TP-A**	-	5	-	-	-	-	5
**TP-B**	7	3	5	-	-	-	15
**MITE**	13	17	-	-	1	-	31
**SMR-A**	79	25	9	-	1	-	114
**SMR-B**	1	2	5	-	1	-	9
Total	106	64	19	-	3	-	192

### Conclusions

The extraordinary genome conservation and shared CRISPR spacer relationships observed between geographically distant isolates of *Hqr. walsbyi* speak of a rapid global dispersal, possibly by airborne salt particles or migratory birds. The strong, global coherence of this species also argues for a strong selection against change and/or a high competitive fitness in hypersaline waters, perhaps related to protection from desiccation by halomucin. It seems that hypersaline waters all over the world represent a type of global pond for this organism. The genomes differ mainly by indels, deletion-coupled insertions, and movements of mobile genetic elements, with deletions occurring frequently via a potentially novel mechanism involving small direct repeats. The high number of pseudogenes, particularly of transposases, in both strains probably results from this, and may reflect a host protective mechanism against mobile genetic elements. Deletion-coupled insertions indicate the uptake and integration of foreign DNA but the mechanisms underlying the generation of these identically placed but unrelated sequences remain to be understood. Strain C23^T^ was probably infected by four distinct transposons upon integration of a foreign sequence of 42.9 kb. At least two distinct mechanisms are used to attack transposons. Repeat-mediated deletions permit trace-free removal of canonical transposons, taking advantage of the associated target duplications. The same mechanism can also inactivate IS605-type transposons (and associated PATEs) by causing element core deletions. In addition, the SMR-A category of small mobile repeats preferentially targets IS605-type transposons and PATEs. The link between PL6-like plasmids and conserved genomic loci related to haloviruses (ViPREs), points to a close relationship between plasmids and viruses in haloarchaea, the details of which remain to be understood.

## Materials and Methods

### Sequence determination and assembly

*Haloquadratum walsbyi* C23^T^ ( = JCM 12705^T^ = DSM 16854^T^) was cultivated in DBCM2 medium as described previously [8]. Total DNA was extracted from cell pellets using the Qiagen Genomic-tip DNA extraction kit (Qiagen, GmbH). Initial sequencing was performed using the Roche GS FLX sequencing platform with LR70 reagents. A total of 69.6 Mb data was collected and assembled using the Newbler software (provided with the sequencing instrument). A combined total of 3.07 Mb resulted from the initial round of assembly with 99.78% of the bases being Q40 or higher, at an average coverage of 22.5 fold. The resulting contigs were ordered according to the genome of HBS001, which gave an early indication of the complete synteny between these two strains. PCR on genomic DNA provided templates to primer walk across the contig gaps. Assembly of the 454 contigs and PCR sequences utilized the Phred–Phrap–Consed package [74].

The reliability of the 454 sequencing results was analyzed. Bases with a low quality values (< 25 from the Newbler software) were checked. Of these 186 could be compared to data from a preliminary sequencing effort. Only 2 differences were observed. Low quality bases within polynucleotide runs, which potentially cause frame-shifts were compared by BLASTX to proteins from haloarchaea with or without the low quality base. This also indicated that most of the low quality bases were correct. Additional potential errors in the 454 sequenced contigs were identified by comparison with the sequence of the closely related HBSQ001 strain, particularly at positions where the C23^T^ base was of low quality and differed from the corresponding base in the other strain. PCR amplification and re-sequencing of these regions in the C23^T^ genome detected a total of 42 errors. Seven additional errors were detected when proteomic data resulted in the identification of pseudogenes. The total number of errors thus computes to 15/Mb. The sequences of primers used for PCR and primer walking are available on request from the corresponding author. The sequences of the main chromosome, PL100, PL6A and PL6B have the following EMBL accession numbers: FR746099, FR746100, FR746101 and FR746102.

### PCR for gap closure and error correction

The TaKaRa LA Taq kit (Takara Bio Inc., Japan) was used for all PCRs. Template was genomic DNA from *Haloquadratum walsbyi* C23^T^ (described above). Reaction cycles and conditions were varied depending upon the expected product length and template difficulty (ISH elements), but a typical scheme was as follows: 50 µl reaction, ∼100 ng DNA: initial denaturation at 94°C, 1 min; 25 cycles of 94°C/30 sec, 58°C/30 sec, 72°C/4 min; final extension 72°C/4 min. Product size and quality was assessed by agarose gel electrophoresis of a 2 µl sample. Where reactions contained subsidiary, unspecific products, the major band was purified by preparative agarose gel electrophoresis. All products were purified to remove primers and salts using the Wizard SV gel and PCR cleanup kit (Promega, GmbH). Sequencing of PCR products was performed using the ABI PRISM BigDye terminator method at the MPI of Biochemistry core sequencing facility.

### Sequencing of large plasmid PL100

Several sequencing reads from an earlier, preliminary sequencing effort could not be matched to the genome sequence based on 454 sequencing. Some of these could be assembled into contigs, indicating that they were not simple contaminants. PCR primers designed from these contigs were then used to detect the presence of a plasmid in some of the subcultures of C23^T^. As the original sequencing reads were mate pairs, this enabled contig adjacencies to be determined, which could be used to close most gaps by a PCR approach using (as a template) plasmid DNA purified from a positive subculture detected in the previous PCR. Due to the low overall sequence coverage, non-assembled singletons in the original dataset were also utilized in phred-phrap assembly, and these enabled further extension of remaining contigs, which could be incorporated by further PCR-based gap closure. Final closure of PL100 was achieved by an all-against-all PCR approach on the few remaining contigs.

### Gene prediction and annotation

The genome annotation and core analyses were performed using the HaloLex system (http://www.halolex.mpg.de, [75]). Further analyses were done using the MIGenAS server (http://www.migenas.org, [76]). The common core of the two strains shows 98.6% sequence identity. Accordingly, there must be a complete consistency with respect to ORF annotations in this shared sequence, including annotation of pseudogenes. An attempt has been made to keep the ORF annotations in the two strains as consistent as possible.

### Bioinformatics tools

A number of specific bioinformatics tools were used, including the following for: CRISPRs (http://crispr.u-psud.fr/), genome alignment using MUMmer (http://mummer.sourceforge.net/), 7S RNA detection (http://bio.lundberg.gu.se/srpscan/), rRNA detection (http://www.cbs.dtu.dk/services/RNAmmer/), tRNA detection with tRNA-scan (http://lowelab.ucsc.edu/tRNAscan-SE/) and Aragorn (http://130.235.46.10/ARAGORN/), tetramer analysis (http://www.megx.net/tetra) [77], genome comparison/alignment (http://asap.ahabs.wisc.edu/mauve/), cumulative skew plots with GenSkewApp (http://www.helmholtz-muenchen.de/en/mips/services/analysis-tools/genskew/index.html), GC-Profile (http://tubic.tju.edu.cn/GC-Profile/), Z-curve plots (http://tubic.tju.edu.cn/zcurve/), GC% with a 2.5 SD cutoff [78], and elements of the EMBOSS suite of bioinformatics programs found within the eBioX program (http://www.ebioinformatics.org). Pilfind was computed at http://signalfind.org/.

### Comparision of the chromosome sequences

A PERL-based script was applied to compare the two chromosome sequences. This script allows a base-by-base comparison but is robust against sequence duplications and indels of any size. The script interprets the pair of sequences as an alternate set of “runs”, defined as subsequences that are completely identical, and “connectors”, which are the divergent sequences that occur between runs. Runs are identified by “word matches”, using exclusively words that are unique in each of the sequences. These words are expanded on both sides as long as the sequences remain identical, which results in a run of a given length. In an initial scaffolding phase, long words (15-mers) are used and only the longest runs (summing up to 30% of the total chromosomal sequences) are considered. The serial order of the runs is determined, which is preserved within synteneic regions of the chromosomes. The result of this phase is an ordered set of runs that build a scaffold of the chromosome sequences. In a second phase, each of the intermediate sequences between runs is subjected to the same procedure except that a short word length (3 bases) is applied and the longest identified run is immediately intercalated into the scaffold. This intercalation creates up to two new but shorter intermediate sequences, one on each side of the newly intercalated run. Intermediate sequences for which no additional run can be identified are “connectors”. Because the algorithm uses trinucleotides as the shortest word size, and thus the minimal length of a run, it performs a base-by-base comparison of the chromosomes. All sequence differences are then represented in the connectors. The procedure allows any length of the connector, from zero (in case of an indel) to more than 100 kb (a huge strain-specific region). As an example, point mutations are connectors of length one in both sequences.

The algorithm can handle sequences that are full of duplications, a common problem in the genomes of halophilic archaea. Only words that are unique in each of the sequences are applied for matching, which excludes words originating from duplications. After this initial matching, sequences are extended on both sides as long as they remain identical. No restrictions apply in this step, so that co-localized copies of duplicated sequences will become part of the corresponding runs. Sequence deviations between the co-localized copies do not cause any problems as they are handled in the second phase, being intermediate sequences between runs. At this stage, additional copies located elsewhere in the genome are no longer relevant.

Most connectors represent trivial sequence differences (e.g. point mutations). For the current project, connectors were selected as non-trivial if they fulfilled one of the following criteria: (a) a length difference of at least 20 bp, (b) the number of single-base differences exceeds 10 in a single connector (which is defined not to have even a single unique trinucleotide match), or (c) the sequence identity is below 50% (which is used only for connectors of at least 7 bp length). These selection criteria are highly relaxed, allowing a detailed comparison of the two chromosomes to be performed. The selected set of 512 non-trivial connectors was inspected manually. The region of difference, and 100–200 bases on each side, were compared to the genome sequences using BLASTN. Cases representing just an enhanced sequence variability (as obvious from a contiguous blast alignment which is free of long gaps) were skipped. The remaining set contained 360 strain-specific sequences. The remainder of the chromosomes, excluding the strain-specific sequences, represents the shared sequence.

## Supporting Information

Figure S1 **Sequence motifs upstream of cdc6 genes in** ***Hqr. walsbyi*** **C23^T^.** At the left of each group of sequences is the Ori name and the locus tag of the nearby *cdc6* gene, containing C for chromosomal or P for plasmid PL100. The direction of the inverted repeats are indicated by > or < at the right of each sequence. Identical bases in the repeats are indicated by green boxes. For comparison, the *Haloferax volcanii ori*C1 ORB sequence is shown above the predicted *ori*C1 of *Hqr. walsbyi.* For details of the predicted DUE, Mini-ORB, G-string and Halo-G-string motifs, see [29].
(TIF)

Table S1 **Strongly under-represented tetramer sequences in** ***Hqr. walsbyi*** **C23^T^ replicons.**
(DOC)

Table S2 **Codon frequencies in** ***Haloquadratum walsbyi.*** The table provides codon frequencies for the 20 amino acids, based on 850405 codons. Start and stop codons were excluded from the computation. The relative amino acid frequencies are given in the “%(total)” column. Frequencies are attributed to their corresponding codons. The first two codon bases are in the column ‘1^st^/2^nd^ BASES’, and the third base is in the column ‘3^rd^ BASE’. The most frequent codon is specified only if the codonset includes an NNT codon. An NNT codon is most frequent for 10 amino acids while an nnA codon is most frequent for 5 amino acids that also use NNT codons.
(DOC)

Table S3 **Mass spectrometry of proteins in a cell membrane preparation of strain C23^T^.**
(DOC)

Table S4 **Categories of strain-specific regions.** Strain-specific sequences were classified into various categories. For mobile genetic elements (MGEs), a distinction is made between transposons and transposase-free MGEs (MITEs, PATEs and other short mobile repeats; Insert_MITE_PATE_SMR). Indels and deletion-coupled insertions (DCI) are categorized as long (>1.5 kb), short (<150 bp) and medium (150 bp–1.5 kb). Indel_PolyRepeat indicates that the copy number for short tandem repeats differs between strains. SwitchRepeats are cases where, at an identical position, are either two distinct repeats or two copies of the same repeat in opposite orientation. Delete_Repeatcore are cases where a transposon or repeat is complete in one strain while the central part has been deleted in the other strain, leaving only the fused terminal sequences. Divergent_Gene refers to the in-situ indels that occur within the repeat regions of the halomucin gene. Finally, there are a few miscellaneous strain-specific regions (Indel_Misc). Some of the categories have been combined in the pie chart of Figure 6.
(DOC)

Table S5 **Listing of all common and strain-specific regions.** As not even a single genome rearrangement has occurred, the alignment of the two chromosomes can be represented by a series of alternating common and strain-specific regions. Common regions, which collectively define the shared sequence, are labeled by a dash in the category column. Categories of strain-specific sequences are those described in Table S4. Position and length are provided for each strain as applicable. For indels that are bounded by direct repeats (of length specified in the overlap column), the core (length) and one copy of the repeat (overlap) are deleted. The total number of deleted bases (sum) is given in the corresponding column. Strain-specific regions can be located in intergenic regions, within genes, or in transposons and repeats (location). The description column provides additional data: (a) Relevant strain-specific regions (DV, GI as given in Table 3). (b) Names of transposons, MITEs, and other short mobile repeats; Extensions in parentheses indicate that the repeat has been targeted by another repeat or has suffered a core-deletion. In the category SwitchRepeats, two elements are specified if they are different or the element is marked (fwd/rev) in cases where the same element occurs in opposite orientation. (c) In several cases, it was possible to determine which of the strains contains the ancestral sequence, e.g. by analysis of gene truncations across indel boundaries. (d) Indels within genes may disrupt the coding region. In cases of “sequence not affected”, the insertion has occurred such that at maximum a few C-terminal residues are affected. In cases of “reading frame conserved”, the indel does not disrupt the reading frame.
(DOC)

Table S6 **Listing of indels which are bounded by direct repeats.** This table is a subset of Table S4, and contains only those indels bounded by direct repeats. The same region numbers are used. All other columns are as described for Table S4. The indels are sorted according to the length in the direct repeat (overlap column). Transposons and MITEs, which cause target duplications, have been excluded from this table.
(DOC)

Table S7 **Sequence matches to CRISPR spacers in strains HBSQ001 and C23^T^.**
(DOC)

Table S8 **Listing of all** ***Haloquadratum*** **MGEs.** MGE codes start either with ISHwa or with HqIRS. MGEs with ISHwa codes are present in (or submitted to) ISFinder. A prerequisite for an ISFinder submission is that the MGE is complete, its boundaries are defined, and the MGE codes for a complete transposase. Element groups are a clustering of MGEs based on sequence similarity and common characteristics. The categories are: TP-A (IS605-type transposon), TP-B (common transposon), MITE (miniature inverted-repeat transposable elements), PATE (palindrome-associated transposable element), or SMR-A, SMR-B (short mobile repeat of types A, B). Length, indicates the length range of complete copies of the element. A hyphen indicates that none of the copies is complete. The ITR and TD fields contain data only if at least one of the elements has complete boundaries on both sides. ITR provides the inverted terminal repeat of a typical member in ISFinder style where the second number corresponds to the ITR length and the first number to the number of matching bases within the ITR. The term “none” indicates that the MGE does not cause target duplications. TD provides the length range of target duplications. The term “none” indicates that the MGE does not cause target duplications. The #(C23^T^) and #(HBSQ001) columns provides the total number of MGEs, followed in parenthesis by the number of elements with complete sequences and the number of elements with complete transposases (devoid of deletions, frame-shifts or in-frame stop codons). The SSEI column provides the number of element copies that occur as SSEIs (strain-specific element insertions). Some of these data are replaced by hyphens if the element is not present in the strain (total number is zero. The remarks column specifies the relationship between MITEs and their parent transposons.
(DOC)

Table S9 **Near terminal palindromes in homogeneous PATEs and relation to IS605 type transposons.** The terminal sequences of homogenous PATEs are compared in several ways. In section A, terminal sequences are compared within each element. The 5′ and 3′ “palindromic end of element” highlights palindromes by comparison of the terminal sequence to its reverse-complement. “Comparison of 5′ and 3′ palindromic ends” highlights similarities between the terminal sequences. For HqIRS46, the 52 bp terminal_only version of the element, generated by repeat-mediated deletion within a perfect 22 bp repeat, is shown (core del). In all cases, internal sequence ends are indicated by three dots. For the 3′ end, both directions show the terminal sequence fragment. It should be noted that the terminal sequences of distinct PATEs show similarities at the very 3′ end. For the 5′ end, the targeting sequence of 4–5 bp is included. The distance between the element start and the near-terminal palindrome is longer at the 5′ end compared to the 3′ end and this extended sequence is not displayed for the reverse strand. In section B, the terminal sequence of the PATE is compared to that of other elements. There is a remarkably high level of sequence similarity between homogenous PATEs from *Haloquadratum* and IS605-type transposases from *Natronomonas pharaonis*.
(DOC)

Text S1 **Descriptions of genomic changes related to MGEs.**
(DOC)
