# Supplementary figures and images for: Haloquadratum walsbyi : Limited Diversity in a Global Pond
Source: PLoS One. 2011 Jun 20;6(6):e20968. doi: 10.1371/journal.pone.0020968 (PMC3119063; doi:10.1371/journal.pone.0020968)

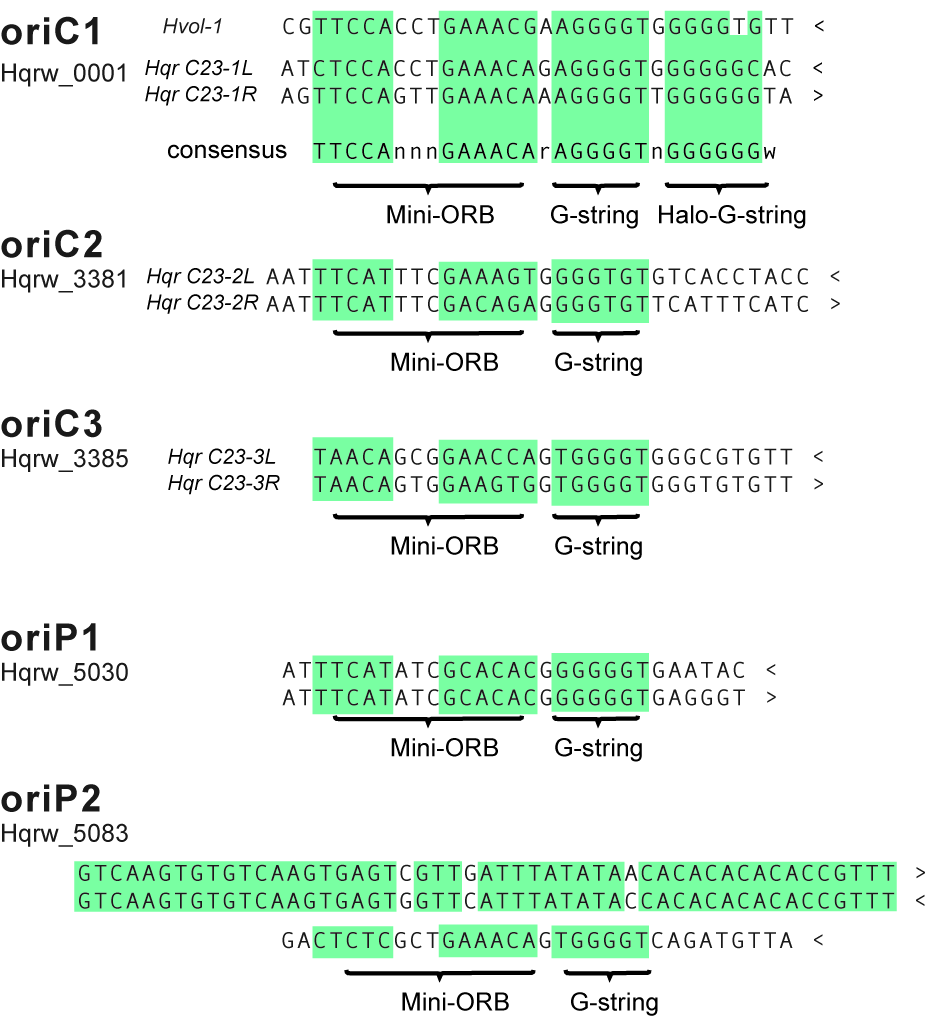

Supplement: Figure S1 — Sequence motifs upstream of cdc6 genes in Hqr. walsbyi C23T. At the left of each group of sequences is the Ori name and the locus tag of the nearby cdc6 gene, containing C for chromosomal or P for plasmid PL100. The direction of the inverted repeats are indicated by > or < at the right of each sequence. Identical bases in the repeats are indicated by green boxes. For comparison, the Haloferax volcanii oriC1 ORB sequence is shown above the predicted oriC1 of Hqr. walsbyi. For details of the predicted DUE, Mini-ORB, G-string and Halo-G-string motifs, see [29]. (TIF) [file pone.0020968.s001.tif]
