# Supplementary material for: Haloquadratum walsbyi : Limited Diversity in a Global Pond
Source: PLoS One. 2011 Jun 20;6(6):e20968. doi: 10.1371/journal.pone.0020968 (PMC3119063; doi:10.1371/journal.pone.0020968)
Supplement: Table S1 — Strongly under-represented tetramer sequences in Hqr. walsbyi C23T replicons. (DOC) [file pone.0020968.s002.doc]

### Table S1. Strongly under-represented tetramer sequences in *Hqr. walsbyi* C23^T^ replicons

| **Replicon** | **Most under-represented Tetramers** | **Comment** |
| --- | --- | --- |
| Chromosome | CTAG, GGCC, AGCT | Z-scores better than 10^-80^, as calculated by the TETRA program of |
| PL100 | GGCC, CGCG | Z-scores better than 10^-17^ |
| PL6A | GGCC (0 sites), CTAG (3 sites) |  |
| PL6B | GGCC (0 sites), CTAG (0 sites) |  |
