# Supplementary material for: Haloquadratum walsbyi : Limited Diversity in a Global Pond
Source: PLoS One. 2011 Jun 20;6(6):e20968. doi: 10.1371/journal.pone.0020968 (PMC3119063; doi:10.1371/journal.pone.0020968)
Supplement: Table S2 — Codon frequencies in Haloquadratum walsbyi. The table provides codon frequencies for the 20 amino acids, based on 850405 codons. Start and stop codons were excluded from the computation. The relative amino acid frequencies are given in the “%(total)” column. Frequencies are attributed to their corresponding codons. The first two codon bases are in the column ‘1st/2nd BASES’, and the third base is in the column ‘3rd BASE’. The most frequent codon is specified only if the codonset includes an NNT codon. An NNT codon is most frequent for 10 amino acids while an nnA codon is most frequent for 5 amino acids that also use NNT codons. (DOC) [file pone.0020968.s003.doc]

**Table S2.** Codon frequencies in *Haloquadratum walsbyi*

|  |  |  | 3^rd^ BASE | | | |  |
| --- | --- | --- | --- | --- | --- | --- | --- |
| AA | %(total) | 1^st^/2^nd^ BASES | A | G | C | T | most frequent codon |
| T | 7.70 | **AC** | 2.96 | 1.67 | 1.42 | 1.66 | ACA |
| A | 9.18 | **GC** | 3.56 | 1.90 | 1.30 | 2.41 | GCA |
| G | 7.31 | **GG** | 2.26 | 1.43 | 1.24 | 2.38 | GGT |
| V | 7.74 | **GT** | 0.96 | 1.41 | 2.45 | 2.92 | GTT |
| P | 4.29 | **CC** | 1.95 | 1.09 | 0.47 | 0.79 | CCA |
| I | 6.13 | **AT** | 0.79 |  | 2.20 | 3.14 | ATT |
| M | 1.47 | **AT** |  | 1.47 |  |  | - |
| N | 3.43 | **AA** |  |  | 1.21 | 2.22 | AAT |
| K | 2.49 | **AA** | 1.44 | 1.05 |  |  | - |
| D | 7.85 | **GA** |  |  | 2.70 | 5.14 | GAT |
| E | 7.65 | **GA** | 3.74 | 3.91 |  |  | - |
| H | 2.16 | **CA** |  |  | 0.86 | 1.30 | CAT |
| Q | 3.47 | **CA** | 1.69 | 1.78 |  |  | - |
| Y | 2.81 | **TA** |  |  | 0.97 | 1.84 | TAT |
| C | 0.81 | **TG** |  |  | 0.25 | 0.56 | TGT |
| W | 1.03 | **TG** |  | 1.03 |  |  | - |
| F | 3.20 | **TT** |  |  | 1.54 | 1.67 | TTT |
| R | 5.86 | **CG** | 1.79 | 1.07 | 1.00 | 1.45 | CGA |
|  |  | **AG** | 0.39 | 0.16 |  |  |  |
| L | 8.25 | **CT** | 0.56 | 1.00 | 2.22 | 2.78 | CTT |
|  |  | **TT** | 0.92 | 0.79 |  |  |  |
| S | 6.82 | **TC** | 2.16 | 1.05 | 0.57 | 0.97 | TCA |
|  |  | **AG** |  |  | 0.84 | 1.23 |  |
