# Supplementary material for: Haloquadratum walsbyi : Limited Diversity in a Global Pond
Source: PLoS One. 2011 Jun 20;6(6):e20968. doi: 10.1371/journal.pone.0020968 (PMC3119063; doi:10.1371/journal.pone.0020968)
Supplement: Table S3 — Mass spectrometry of proteins in a cell membrane preparation of strain C23T. (DOC) [file pone.0020968.s004.doc]

### Table S3. Mass spectrometry of proteins in a cell membrane preparation of strain C23^T^

| Protein Locus Tag | Description | Peptides | Tryptic peptide masses (sequence) |
| --- | --- | --- | --- |
| Hqrw_1016 | bacteriorhodopsin I | 4 | 807.4239 (STFNALR)  1006.5236 (VVDVYWAR)  1020.612 (VGFGFILLR)  1788.7836 (AIMGGGSEPTPSAQETAAD) |
| Hqrw_1019 | bacteriorhodopsin II | 1 | 1106.572 (SAETGIIFNR)* |
| Hqrw_1021 | carotenase (Brp-like protein) | 4 | 758.46504 (ALSIVTR)  1164.5622 (AVSESLSDTTR) |
| Hqrw_3587 | halorhodopsin | 1 | 1128.6332 (YAFSFLLLR)* |
| Hqrw_1237 | cell surface glycoprotein | 11 | 893.4243 (IDQSEFR)  1031.5764 (LLSLPGNYR)  1216.6452 (DEVSVIFVGPR)  1506.6951 (DNIDVSDAENIFR)  1637.7169 (DQGDYELVDIDNSR)  1769.8908 (LGVIDAQDAQNDSGIVR)  2382.1088 (IDQSEFRDNIDVSDAENIFR)  2495.2027 (SQIVANSVDDTASDDLIVTETFR)  2623.2977 (SQIVANSVDDTASDDLIVTETFRK)  2837.2588 (SIAVEGDNQFDEEDVQLSSSGGDLGNR)  3339.4188 (DNEFGDDFATSVSGFNSQVDNNFSGTADQVR) |
| Hqrw_1240 | probable surface protein | 2 | 1396.6623 (VIAFGQFEDTDR)  1532.7947 (IGFNLQNIGTETAR) |
| Hqrw_1641 | probable surface protein | 15 | 940.44028 (NDYFINR)  980.48148 (DVTGADLYK)  992.5291 (FKLEVSDR)  1026.5233 (TYSLDTSIK)  1285.703 (AIPQEYPLTVR)  1306.6517 (TGSVAVSNGPQYK)  1458.7427 (TAQQSLGTIAAGDAR)  1463.7845 (QIGDIAQVVQTHR)  1936.9854 (SFAVTNTAAVTAGQTETLR)  2069.0099 (AMYVDSIGSVAVTVTNTGER)  2322.2042 (MGLTIEVDQHAQPGVYQLPVK)  2421.1965 (ITYGYINSINVDKNDYFINR)  2765.3508 (VQNTGSEIAQNAELSIVQSEYFNPK)  2781.3345 (IIHDDTYGNDVTSDPLSVDVPVGPEK)  2785.4498 (LSPIEPFALVSTSASLGTLNPGESATAR) |
| Hqrw_2184 | probable surface protein | 3 | 1262.6466 (LSGGSTSAVTLDR)  1598.8516 (QTPGAVDLTGVDLSVK)  2454.2966 (VAVSDATVTPATPTAGAPTTVTATVR |
| Hqrw_6007 | PL6A hypothetical protein | 3 | 907.43995 (DVDAYVAR)  1135.4928 (NDISQSEGMR)  1349.6212 (EQFEDVNSINR) |
| Hqrw_7007 | PL6B hypothetical protein | 4 | 2204.9757 (RVEEYQDEHNISEAEAMR)  2361.0768 (RVEEYQDEHNISEAEAMRR)  2513.2133 (LDNDASEETDTKPDILKDEPLR)  2813.2475 (GLDHTDDDEASVENIQDDLDEILSR) |

*Although only single peptides were identified for these ORFs, they were detected on repeated analyses, and had high probability scores
 (PEP < 10^-9^), Mascot scores (> 48) and intensities (>170000)
