# Supplementary material for: Haloquadratum walsbyi : Limited Diversity in a Global Pond
Source: PLoS One. 2011 Jun 20;6(6):e20968. doi: 10.1371/journal.pone.0020968 (PMC3119063; doi:10.1371/journal.pone.0020968)
Supplement: Table S4 — Categories of strain-specific regions. Strain-specific sequences were classified into various categories. For mobile genetic elements (MGEs), a distinction is made between transposons and transposase-free MGEs (MITEs, PATEs and other short mobile repeats; Insert_MITE_PATE_SMR). Indels and deletion-coupled insertions (DCI) are categorized as long (>1.5 kb), short (<150 bp) and medium (150 bp–1.5 kb). Indel_PolyRepeat indicates that the copy number for short tandem repeats differs between strains. SwitchRepeats are cases where, at an identical position, are either two distinct repeats or two copies of the same repeat in opposite orientation. Delete_Repeatcore are cases where a transposon or repeat is complete in one strain while the central part has been deleted in the other strain, leaving only the fused terminal sequences. Divergent_Gene refers to the in-situ indels that occur within the repeat regions of the halomucin gene. Finally, there are a few miscellaneous strain-specific regions (Indel_Misc). Some of the categories have been combined in the pie chart of Figure 6. (DOC) [file pone.0020968.s005.doc]

### Table S4. Categories of strain-specific regions

| **Category** | **Total** | **C23^T^** | **HBSQ001** | **Label in Fig. 6** |
| --- | --- | --- | --- | --- |
| Indel_Medium | 33 | 22 | 12 | Indels Other |
| Indel_Short | 41 | 22 | 23 | Indels Other |
| Indel_Long | 25 | 10 | 17 | Indels Long |
| Replacement_Long_Long | 16 | 16 | 16 | DCI Long |
| Replacement_Long_Medium | 15 | 15 | 15 | DCI Long |
| Replacement_Long_Short | 2 | 2 | 2 | DCI Long |
| Replacement_Medium_Medium | 4 | 4 | 4 | DCI Other |
| Replacement_Medium_Short | 5 | 5 | 5 | DCI Other |
| Replacement_Short_Short | 20 | 20 | 20 | DCI Other |
| Insert_Transposon | 43 | 14 | 42 | Transposons |
| Insert_MITE_PATE_SMR | 111 | 80 | 85 | Transposase-free MGEs (MITEs, PATEs & SMRs) |
| Indel_PolyRepeat | 31 | 31 | 31 | Polyrepeats |
| Replacement_SwitchRepeats | 5 | 5 | 5 | Miscellaneous |
| Delete_Repeatcore | 5 | 4 | 1 | Miscellaneous |
| Divergent_Gene | 1 | 1 | 1 | Miscellaneous |
| Indel_Misc | 3 | 3 | 3 | Miscellaneous |
| (Sum) | 360 | 254 | 282 |  |
