# Supplementary material for: Haloquadratum walsbyi : Limited Diversity in a Global Pond
Source: PLoS One. 2011 Jun 20;6(6):e20968. doi: 10.1371/journal.pone.0020968 (PMC3119063; doi:10.1371/journal.pone.0020968)
Supplement: Table S5 — Listing of all common and strain-specific regions. As not even a single genome rearrangement has occurred, the alignment of the two chromosomes can be represented by a series of alternating common and strain-specific regions. Common regions, which collectively define the shared sequence, are labeled by a dash in the category column. Categories of strain-specific sequences are those described in Table S4. Position and length are provided for each strain as applicable. For indels that are bounded by direct repeats (of length specified in the overlap column), the core (length) and one copy of the repeat (overlap) are deleted. The total number of deleted bases (sum) is given in the corresponding column. Strain-specific regions can be located in intergenic regions, within genes, or in transposons and repeats (location). The description column provides additional data: (a) Relevant strain-specific regions (DV, GI as given in Table 3). (b) Names of transposons, MITEs, and other short mobile repeats; Extensions in parentheses indicate that the repeat has been targeted by another repeat or has suffered a core-deletion. In the category SwitchRepeats, two elements are specified if they are different or the element is marked (fwd/rev) in cases where the same element occurs in opposite orientation. (c) In several cases, it was possible to determine which of the strains contains the ancestral sequence, e.g. by analysis of gene truncations across indel boundaries. (d) Indels within genes may disrupt the coding region. In cases of “sequence not affected”, the insertion has occurred such that at maximum a few C-terminal residues are affected. In cases of “reading frame conserved”, the indel does not disrupt the reading frame. (DOC) [file pone.0020968.s006.doc]

### Table S5. Listing of all common and strain-specific regions

| **Region Number** | **Region Category** | **Strain C23^T^** | | **Strain HBSQ001** | | **Overlap** | **SUM (len.+overl.)** | **Location** | **Description** |
| --- | --- | --- | --- | --- | --- | --- | --- | --- | --- |
|  |  | **position** | **length** | **position** | **length** |  |  |  |  |
| 1 | - | 1-28061 | 28061 | 1-28012 | 28012 | - | - | - | - |
| 2 | DELETE_REPEATCORE | - | - | 28013-28332 | 320 | 19 | 339 | in_repeat | HqIRS46(core_deletion) |
| 3 | - | 28043-32370 | 4328 | 28333-32661 | 4329 | - | - | - | - |
| 4 | INSERT_MITE | - | - | 32662-32921 | 260 | 10 | 270 | intergenic | HqIRS35 |
| 5 | - | 32361-37014 | 4654 | 32922-37576 | 4655 | - | - | - | - |
| 6 | INDEL_SHORT | - | - | 37577-37637 | 61 | 3 | 64 | intergenic | - |
| 7 | - | 37012-72246 | 35235 | 37638-72889 | 35252 | - | - | - | - |
| 8 | INDEL_POLYREPEAT | 72247-72258 | 12 | 72890-72931 | 42 | - | - | intergenic | - |
| 9 | - | 72259-73397 | 1139 | 72932-74054 | 1123 | - | - | - | - |
| 10 | INDEL_SHORT | 73398-73413 | 16 | - | - | 8 | 24 | intergenic | - |
| 11 | - | 73414-75791 | 2378 | 74047-76423 | 2377 | - | - | - | - |
| 12 | INDEL_MEDIUM | - | - | 76424-77200 | 777 | 1 | 778 | intergenic | - |
| 13 | - | 75791-86133 | 10343 | 77201-87528 | 10328 | - | - | - | - |
| 14 | INDEL_POLYREPEAT | 86134-86171 | 38 | 87529-87586 | 58 | - | - | intergenic | - |
| 15 | - | 86172-87527 | 1356 | 87587-88942 | 1356 | - | - | - | - |
| 16 | REPLACEMENT_MEDIUM_SHORT | 87528-87684 | 157 | 88943-88952 | 10 | - | - | intergenic | - |
| 17 | - | 87685-88268 | 584 | 88953-89536 | 584 | - | - | - | - |
| 18 | INDEL_POLYREPEAT | 88269-88340 | 72 | 89537-89578 | 42 | - | - | in_gene | reading frame conserved |
| 19 | - | 88341-96359 | 8019 | 89579-97598 | 8020 | - | - | - | - |
| 20 | REPLACEMENT_SHORT_SHORT | 96360-96434 | 75 | 97599-97670 | 72 | - | - | intergenic | - |
| 21 | - | 96435-96480 | 46 | 97671-97716 | 46 | - | - | - | - |
| 22 | INDEL_LONG | 96481-101440 | 4960 | - | - | - | - | intergenic | - |
| 23 | - | 101441-108447 | 7007 | 97717-104715 | 6999 | - | - | - | - |
| 24 | DIVERGENT_GENE | 108448-129921 | 21474 | 104716-130155 | 25440 | - | - | N/A | DV1 |
| 25 | - | 129922-149824 | 19903 | 130156-150024 | 19869 | - | - | - | - |
| 26 | INDEL_MEDIUM | - | - | 150025-150342 | 318 | - | - | intergenic | - |
| 27 | - | 149825-155531 | 5707 | 150343-156042 | 5700 | - | - | - | - |
| 28 | INDEL_SHORT | 155532-155553 | 22 | 156043-156043 | 1 | - | - | intergenic | - |
| 29 | - | 155554-175647 | 20094 | 156044-176146 | 20103 | - | - | - | - |
| 30 | INSERT_REPEAT | - | - | 176147-176255 | 109 | - | - | in_repeat | HqIRS42 |
| 31 | - | 175648-178256 | 2609 | 176256-178870 | 2615 | - | - | - | - |
| 32 | DELETE_REPEATCORE | - | - | 178871-179186 | 316 | 22 | 338 | in_repeat | HqIRS46(core_deletion) |
| 33 | - | 178235-183417 | 5183 | 179187-184369 | 5183 | - | - | - | - |
| 34 | INDEL_MISC | 183418-183713 | 296 | 184370-184708 | 339 | - | - | in_repeat | - |
| 35 | - | 183714-193385 | 9672 | 184709-194368 | 9660 | - | - | - | - |
| 36 | INSERT_REPEAT | 193386-193390 | 5 | 194369-194479 | 111 | - | - | intergenic | HqIRS42 |
| 37 | - | 193391-201883 | 8493 | 194480-202980 | 8501 | - | - | - | - |
| 38 | INSERT_REPEAT | 201884-201888 | 5 | 202981-203088 | 108 | - | - | in_repeat | HqIRS43 |
| 39 | - | 201889-212552 | 10664 | 203089-213753 | 10665 | - | - | - | - |
| 40 | INSERT_TRANSPOSON | 212553-214011 | 1459 | - | - | 5 | 1464 | intergenic | ISHwa13 |
| 41 | - | 214012-237960 | 23949 | 213749-237727 | 23979 | - | - | - | - |
| 42 | INDEL_SHORT | 237961-238096 | 136 | - | - | 1 | 137 | intergenic | - |
| 43 | - | 238097-239384 | 1288 | 237727-239014 | 1288 | - | - | - | - |
| 44 | INDEL_POLYREPEAT | 239385-239462 | 78 | 239015-239065 | 51 | - | - | intergenic | - |
| 45 | - | 239463-240263 | 801 | 239066-239866 | 801 | - | - | - | - |
| 46 | INSERT_TRANSPOSON | 240264-243454 | 3191 | 239867-239870 | 4 | - | - | intergenic | ISHwa26(targeted) |
| 47 | - | 243455-248608 | 5154 | 239871-245028 | 5158 | - | - | - | - |
| 48 | INSERT_REPEAT | 248609-248718 | 110 | - | - | 1 | 111 | in_gene | sequence not affected; HqIRS55 |
| 49 | - | 248719-260965 | 12247 | 245028-257292 | 12265 | - | - | - | - |
| 50 | REPLACEMENT_LONG_LONG | 260966-269209 | 8244 | 257293-301070 | 43778 | - | - | N/A | DV2,GI1; ancestral: C23 |
| 51 | - | 269210-278596 | 9387 | 301071-310452 | 9382 | - | - | - | - |
| 52 | INSERT_REPEAT | 278597-278600 | 4 | 310453-310504 | 52 | - | - | intergenic | ISHwa23(core_deletion) |
| 53 | - | 278601-283437 | 4837 | 310505-315345 | 4841 | - | - | - | - |
| 54 | INSERT_TRANSPOSON | 283438-284948 | 1511 | - | - | 8 | 1519 | intergenic | ISHwa9 |
| 55 | - | 284949-301552 | 16604 | 315338-331956 | 16619 | - | - | - | - |
| 56 | INDEL_POLYREPEAT | 301553-301588 | 36 | 331957-332016 | 60 | - | - | in_gene | reading frame conserved |
| 57 | - | 301589-312381 | 10793 | 332017-342805 | 10789 | - | - | - | - |
| 58 | INDEL_MEDIUM | 312382-312700 | 319 | - | - | - | - | intergenic | - |
| 59 | - | 312701-314811 | 2111 | 342806-344923 | 2118 | - | - | - | - |
| 60 | INSERT_REPEAT | 314812-314916 | 105 | - | - | 6 | 111 | in_repeat | HqIRS55 |
| 61 | - | 314917-337258 | 22342 | 344918-367266 | 22349 | - | - | - | - |
| 62 | INSERT_TRANSPOSON | 337259-338767 | 1509 | - | - | 5 | 1514 | in_transposon | ISHwa12 |
| 63 | - | 338768-343182 | 4415 | 367262-371675 | 4414 | - | - | - | - |
| 64 | INSERT_REPEAT | 343183-343479 | 297 | 371676-371676 | 1 | - | - | in_transposon | HqIRS40 |
| 65 | - | 343480-344385 | 906 | 371677-372583 | 907 | - | - | - | - |
| 66 | INDEL_MEDIUM | - | - | 372584-372902 | 319 | 1 | 320 | intergenic | - |
| 67 | - | 344385-345711 | 1327 | 372903-374241 | 1339 | - | - | - | - |
| 68 | REPLACEMENT_MEDIUM_SHORT | 345712-346128 | 417 | 374242-374255 | 14 | - | - | intergenic | - |
| 69 | - | 346129-358217 | 12089 | 374256-386344 | 12089 | - | - | - | - |
| 70 | INDEL_SHORT | 358218-358230 | 13 | - | - | 7 | 20 | intergenic | - |
| 71 | - | 358231-358372 | 142 | 386338-386479 | 142 | - | - | - | - |
| 72 | INDEL_SHORT | 358373-358417 | 45 | - | - | - | - | intergenic | - |
| 73 | - | 358418-358518 | 101 | 386480-386579 | 100 | - | - | - | - |
| 74 | INSERT_MITE | - | - | 386580-386699 | 120 | 8 | 128 | intergenic | HqIRS39 |
| 75 | - | 358511-381540 | 23030 | 386700-409758 | 23059 | - | - | - | - |
| 76 | INDEL_MISC | 381541-381768 | 228 | 409759-409810 | 52 | - | - | in_transposon | - |
| 77 | - | 381769-383111 | 1343 | 409811-411153 | 1343 | - | - | - | - |
| 78 | INSERT_REPEAT | 383112-383115 | 4 | 411154-411707 | 554 | - | - | intergenic | HqIRS57 |
| 79 | - | 383116-384347 | 1232 | 411708-412946 | 1239 | - | - | - | - |
| 80 | INSERT_REPEAT | 384348-384351 | 4 | 412947-413511 | 565 | - | - | intergenic | HqIRS71 |
| 81 | - | 384352-388188 | 3837 | 413512-417346 | 3835 | - | - | - | - |
| 82 | INSERT_REPEAT | 388189-388298 | 110 | - | - | - | - | intergenic | HqIRS55 |
| 83 | - | 388299-391681 | 3383 | 417347-420733 | 3387 | - | - | - | - |
| 84 | INSERT_MITE | - | - | 420734-420987 | 254 | 10 | 264 | intergenic | HqIRS35 |
| 85 | - | 391672-392507 | 836 | 420988-421822 | 835 | - | - | - | - |
| 86 | INSERT_REPEAT | - | - | 421823-421931 | 109 | - | - | in_transposon | HqIRS42 |
| 87 | - | 392508-392539 | 32 | 421932-421963 | 32 | - | - | - | - |
| 88 | REPLACEMENT_LONG_LONG | 392540-417971 | 25432 | 421964-428610 | 6647 | - | - | N/A | DV3; ancestral: HBSQ001 |
| 89 | - | 417972-419652 | 1681 | 428611-430291 | 1681 | - | - | - | - |
| 90 | INDEL_LONG | 419653-422375 | 2723 | - | - | - | - | intergenic | - |
| 91 | - | 422376-426514 | 4139 | 430292-434431 | 4140 | - | - | - | - |
| 92 | INSERT_MITE | 426515-426930 | 416 | - | - | 8 | 424 | intergenic | HqIRS32 |
| 93 | - | 426931-429677 | 2747 | 434424-437152 | 2729 | - | - | - | - |
| 94 | INSERT_REPEAT | 429678-429785 | 108 | - | - | - | - | in_repeat | HqIRS43 |
| 95 | - | 429786-441788 | 12003 | 437153-449155 | 12003 | - | - | - | - |
| 96 | INDEL_MEDIUM | - | - | 449156-449855 | 700 | - | - | in_gene | - |
| 97 | - | 441789-442595 | 807 | 449856-450665 | 810 | - | - | - | - |
| 98 | INSERT_TRANSPOSON | 442596-442599 | 4 | 450666-452523 | 1858 | - | - | intergenic | ISHwa21(targeted) |
| 99 | - | 442600-446867 | 4268 | 452524-456792 | 4269 | - | - | - | - |
| 100 | INDEL_SHORT | - | - | 456793-456804 | 12 | 12 | 24 | intergenic | - |
| 101 | - | 446856-463247 | 16392 | 456805-473201 | 16397 | - | - | - | - |
| 102 | INSERT_REPEAT | - | - | 473202-473309 | 108 | - | - | in_transposon | HqIRS43 |
| 103 | - | 463248-469333 | 6086 | 473310-479395 | 6086 | - | - | - | - |
| 104 | INDEL_POLYREPEAT | 469334-469374 | 41 | 479396-479460 | 65 | - | - | in_gene | reading frame conserved |
| 105 | - | 469375-472968 | 3594 | 479461-483054 | 3594 | - | - | - | - |
| 106 | INDEL_LONG | 472969-477786 | 4818 | - | - | 3 | 4821 | intergenic | - |
| 107 | - | 477787-484231 | 6445 | 483052-489473 | 6422 | - | - | - | - |
| 108 | INDEL_SHORT | 484232-484247 | 16 | - | - | 10 | 26 | intergenic | - |
| 109 | - | 484248-493403 | 9156 | 489464-498627 | 9164 | - | - | - | - |
| 110 | INSERT_REPEAT | 493404-493407 | 4 | 498628-499206 | 579 | - | - | intergenic | HqIRS71 |
| 111 | - | 493408-516425 | 23018 | 499207-522207 | 23001 | - | - | - | - |
| 112 | INSERT_MITE | 516426-516590 | 165 | - | - | 8 | 173 | intergenic | HqIRS36 |
| 113 | - | 516591-517065 | 475 | 522200-522674 | 475 | - | - | - | - |
| 114 | INSERT_REPEAT | 517066-517070 | 5 | 522675-522726 | 52 | - | - | intergenic | HqIRS46(core_deletion) |
| 115 | - | 517071-524851 | 7781 | 522727-530496 | 7770 | - | - | - | - |
| 116 | INSERT_REPEAT | 524852-525237 | 386 | - | - | - | - | intergenic | HqIRS46 |
| 117 | - | 525238-537690 | 12453 | 530497-542965 | 12469 | - | - | - | - |
| 118 | INSERT_MITE | - | - | 542966-543130 | 165 | 8 | 173 | intergenic | HqIRS36 |
| 119 | - | 537683-560070 | 22388 | 543131-565544 | 22414 | - | - | - | - |
| 120 | INDEL_LONG | 560071-588686 | 28616 | - | - | - | - | in_gene | DV4; ancestral: C23 |
| 121 | - | 588687-600334 | 11648 | 565545-577189 | 11645 | - | - | - | - |
| 122 | INDEL_SHORT | 600335-600335 | 1 | 577190-577236 | 47 | - | - | in_repeat | - |
| 123 | - | 600336-620628 | 20293 | 577237-597521 | 20285 | - | - | - | - |
| 124 | REPLACEMENT_LONG_MEDIUM | 620629-621046 | 418 | 597522-604178 | 6657 | - | - | N/A | ancestral: C23 |
| 125 | - | 621047-626668 | 5622 | 604179-609800 | 5622 | - | - | - | - |
| 126 | INSERT_REPEAT | 626669-626673 | 5 | 609801-609852 | 52 | - | - | intergenic | HqIRS46(core_deletion) |
| 127 | - | 626674-639178 | 12505 | 609853-622344 | 12492 | - | - | - | - |
| 128 | INSERT_REPEAT | 639179-639289 | 111 | 622345-622345 | 1 | - | - | intergenic | HqIRS55 |
| 129 | - | 639290-657190 | 17901 | 622346-640230 | 17885 | - | - | - | - |
| 130 | INDEL_MEDIUM | 657191-657382 | 192 | - | - | - | - | intergenic | - |
| 131 | - | 657383-659550 | 2168 | 640231-642397 | 2167 | - | - | - | - |
| 132 | REPLACEMENT_SWITCHREPEATS | 659551-659658 | 108 | 642398-642505 | 108 | - | - | in_repeat | HqIRS43(fwd/rev) |
| 133 | - | 659659-665005 | 5347 | 642506-647854 | 5349 | - | - | - | - |
| 134 | DELETE_REPEATCORE | - | - | 647855-648174 | 320 | 19 | 339 | in_repeat | HqIRS46(core_deletion) |
| 135 | - | 664987-669803 | 4817 | 648175-653001 | 4827 | - | - | - | - |
| 136 | INSERT_REPEAT | 669804-670359 | 556 | 653002-653005 | 4 | - | - | intergenic | HqIRS56 |
| 137 | - | 670360-670571 | 212 | 653006-653217 | 212 | - | - | - | - |
| 138 | INDEL_SHORT | - | - | 653218-653238 | 21 | - | - | intergenic | - |
| 139 | - | 670572-695550 | 24979 | 653239-678220 | 24982 | - | - | - | - |
| 140 | INSERT_TRANSPOSON | 695551-697218 | 1668 | - | - | 11 | 1679 | intergenic | ISHwa2 |
| 141 | - | 697219-711080 | 13862 | 678210-692078 | 13869 | - | - | - | - |
| 142 | INSERT_REPEAT | 711081-711624 | 544 | 692079-692082 | 4 | - | - | intergenic | HqIRS56 |
| 143 | - | 711625-713606 | 1982 | 692083-694065 | 1983 | - | - | - | - |
| 144 | INSERT_REPEAT | 713607-713610 | 4 | 694066-694628 | 563 | - | - | intergenic | HqIRS71 |
| 145 | - | 713611-714490 | 880 | 694629-695510 | 882 | - | - | - | - |
| 146 | INSERT_REPEAT | 714491-714494 | 4 | 695511-696032 | 522 | - | - | intergenic | HqIRS71 |
| 147 | - | 714495-714549 | 55 | 696033-696087 | 55 | - | - | - | - |
| 148 | INDEL_MEDIUM | 714550-714986 | 437 | - | - | 7 | 444 | intergenic | - |
| 149 | - | 714987-716377 | 1391 | 696081-697482 | 1402 | - | - | - | - |
| 150 | INDEL_SHORT | - | - | 697483-697575 | 93 | 13 | 106 | intergenic | - |
| 151 | - | 716365-722321 | 5957 | 697576-703532 | 5957 | - | - | - | - |
| 152 | INDEL_POLYREPEAT | 722322-722365 | 44 | 703533-703546 | 14 | - | - | in_gene | reading frame conserved |
| 153 | - | 722366-724686 | 2321 | 703547-705866 | 2320 | - | - | - | - |
| 154 | INSERT_REPEAT | - | - | 705867-705976 | 110 | - | - | in_repeat | HqIRS43 |
| 155 | - | 724687-734032 | 9346 | 705977-715331 | 9355 | - | - | - | - |
| 156 | INDEL_POLYREPEAT | 734033-734046 | 14 | 715332-715361 | 30 | - | - | intergenic | - |
| 157 | - | 734047-736577 | 2531 | 715362-717888 | 2527 | - | - | - | - |
| 158 | INDEL_SHORT | 736578-736598 | 21 | - | - | 3 | 24 | intergenic | - |
| 159 | - | 736599-745875 | 9277 | 717886-727158 | 9273 | - | - | - | - |
| 160 | INDEL_SHORT | 745876-745904 | 29 | - | - | 1 | 30 | in_gene | - |
| 161 | - | 745905-746236 | 332 | 727158-727485 | 328 | - | - | - | - |
| 162 | REPLACEMENT_LONG_LONG | 746237-757917 | 11681 | 727486-738373 | 10888 | - | - | N/A | DV5; ancestral: C23 |
| 163 | - | 757918-780033 | 22116 | 738374-760501 | 22128 | - | - | - | - |
| 164 | INSERT_REPEAT | 780034-780143 | 110 | - | - | 1 | 111 | intergenic | HqIRS55 |
| 165 | - | 780144-787105 | 6962 | 760501-767453 | 6953 | - | - | - | - |
| 166 | INSERT_REPEAT | 787106-787110 | 5 | 767454-767843 | 390 | - | - | intergenic | HqIRS46 |
| 167 | - | 787111-826294 | 39184 | 767844-807045 | 39202 | - | - | - | - |
| 168 | INSERT_TRANSPOSON | 826295-827239 | 945 | - | - | 8 | 953 | intergenic | HqIRS11 |
| 169 | - | 827240-851716 | 24477 | 807038-831513 | 24476 | - | - | - | - |
| 170 | INSERT_REPEAT | 851717-851824 | 108 | - | - | 3 | 111 | intergenic | HqIRS55 |
| 171 | - | 851825-851911 | 87 | 831511-831597 | 87 | - | - | - | - |
| 172 | INDEL_MEDIUM | - | - | 831598-831987 | 390 | - | - | intergenic | - |
| 173 | - | 851912-862458 | 10547 | 831988-842534 | 10547 | - | - | - | - |
| 174 | INSERT_REPEAT | 862459-862463 | 5 | 842535-842587 | 53 | - | - | intergenic | ISHwa17(core_deletion) |
| 175 | - | 862464-870166 | 7703 | 842588-850292 | 7705 | - | - | - | - |
| 176 | INSERT_MITE | 870167-870420 | 254 | - | - | 10 | 264 | in_repeat | HqIRS35 |
| 177 | - | 870421-886968 | 16548 | 850283-866826 | 16544 | - | - | - | - |
| 178 | REPLACEMENT_MEDIUM_SHORT | 886969-886993 | 25 | 866827-868305 | 1479 | - | - | intergenic | - |
| 179 | - | 886994-929578 | 42585 | 868306-910862 | 42557 | - | - | - | - |
| 180 | INSERT_MITE | 929579-929697 | 119 | 910863-910871 | 9 | - | - | intergenic | HqIRS39 |
| 181 | - | 929698-933026 | 3329 | 910872-914201 | 3330 | - | - | - | - |
| 182 | INDEL_MEDIUM | 933027-933029 | 3 | 914202-914770 | 569 | - | - | in_transposon | - |
| 183 | - | 933030-933373 | 344 | 914771-915116 | 346 | - | - | - | - |
| 184 | INSERT_MITE | 933374-933633 | 260 | - | - | 10 | 270 | in_transposon | HqIRS35 |
| 185 | - | 933634-934612 | 979 | 915107-916097 | 991 | - | - | - | - |
| 186 | REPLACEMENT_SHORT_SHORT | 934613-934657 | 45 | 916098-916141 | 44 | - | - | in_transposon | - |
| 187 | - | 934658-959417 | 24760 | 916142-940901 | 24760 | - | - | - | - |
| 188 | INSERT_REPEAT | 959418-959424 | 7 | 940902-941309 | 408 | - | - | intergenic | HqIRS44 |
| 189 | - | 959425-963299 | 3875 | 941310-945184 | 3875 | - | - | - | - |
| 190 | INSERT_TRANSPOSON | 963300-964961 | 1662 | - | - | 10 | 1672 | in_repeat | ISHwa2 |
| 191 | - | 964962-978529 | 13568 | 945175-958729 | 13555 | - | - | - | - |
| 192 | REPLACEMENT_SWITCHREPEATS | 978530-978637 | 108 | 958730-958837 | 108 | - | - | intergenic | HqIRS43/HqIRS42 |
| 193 | - | 978638-982423 | 3786 | 958838-962623 | 3786 | - | - | - | - |
| 194 | INSERT_REPEAT | 982424-982531 | 108 | - | - | - | - | in_repeat | HqIRS42 |
| 195 | - | 982532-1006463 | 23932 | 962624-986535 | 23912 | - | - | - | - |
| 196 | INSERT_TRANSPOSON | 1006464-1007748 | 1285 | - | - | 7 | 1292 | intergenic | ISHwa4 |
| 197 | - | 1007749-1008268 | 520 | 986529-987048 | 520 | - | - | - | - |
| 198 | INDEL_SHORT | 1008269-1008290 | 22 | - | - | 4 | 26 | intergenic | - |
| 199 | - | 1008291-1008340 | 50 | 987045-987094 | 50 | - | - | - | - |
| 200 | INSERT_REPEAT | - | - | 987095-987202 | 108 | - | - | in_repeat | HqIRS43 |
| 201 | - | 1008341-1009237 | 897 | 987203-988099 | 897 | - | - | - | - |
| 202 | INSERT_REPEAT | - | - | 988100-988209 | 110 | 1 | 111 | intergenic | HqIRS55 |
| 203 | - | 1009237-1010362 | 1126 | 988210-989333 | 1124 | - | - | - | - |
| 204 | INSERT_REPEAT | 1010363-1010368 | 6 | 989334-990005 | 672 | - | - | intergenic | HqIRS56(targeted) |
| 205 | - | 1010369-1032637 | 22269 | 990006-1012260 | 22255 | - | - | - | - |
| 206 | INSERT_REPEAT | 1032638-1032745 | 108 | - | - | - | - | in_repeat | HqIRS43 |
| 207 | - | 1032746-1045383 | 12638 | 1012261-1024896 | 12636 | - | - | - | - |
| 208 | INDEL_POLYREPEAT | 1045384-1045419 | 36 | 1024897-1024911 | 15 | - | - | intergenic | - |
| 209 | - | 1045420-1047899 | 2480 | 1024912-1027390 | 2479 | - | - | - | - |
| 210 | INSERT_TRANSPOSON | 1047900-1049409 | 1510 | - | - | 9 | 1519 | in_repeat | ISHwa8 |
| 211 | - | 1049410-1069358 | 19949 | 1027382-1047329 | 19948 | - | - | - | - |
| 212 | INSERT_TRANSPOSON | 1069359-1069365 | 7 | 1047330-1049368 | 2039 | - | - | in_gene | ISHwa17 |
| 213 | - | 1069366-1077976 | 8611 | 1049369-1057974 | 8606 | - | - | - | - |
| 214 | INSERT_REPEAT | - | - | 1057975-1058082 | 108 | - | - | in_repeat | HqIRS43 |
| 215 | - | 1077977-1097186 | 19210 | 1058083-1077343 | 19261 | - | - | - | - |
| 216 | INSERT_MITE | - | - | 1077344-1077508 | 165 | 9 | 174 | intergenic | HqIRS36 |
| 217 | - | 1097178-1098102 | 925 | 1077509-1078433 | 925 | - | - | - | - |
| 218 | INSERT_MITE | - | - | 1078434-1078598 | 165 | 8 | 173 | intergenic | HqIRS36 |
| 219 | - | 1098095-1098763 | 669 | 1078599-1079272 | 674 | - | - | - | - |
| 220 | INDEL_LONG | 1098764-1101694 | 2931 | - | - | 1 | 2932 | intergenic | - |
| 221 | - | 1101695-1102479 | 785 | 1079272-1080056 | 785 | - | - | - | - |
| 222 | INSERT_TRANSPOSON | 1102480-1104241 | 1762 | - | - | 5 | 1767 | in_gene | ISHwa13 |
| 223 | - | 1104242-1108746 | 4505 | 1080052-1084549 | 4498 | - | - | - | - |
| 224 | INSERT_REPEAT | 1108747-1108751 | 5 | 1084550-1085196 | 647 | - | - | in_gene | HqIRS56(targeted) |
| 225 | - | 1108752-1111704 | 2953 | 1085197-1088149 | 2953 | - | - | - | - |
| 226 | INDEL_MEDIUM | - | - | 1088150-1088462 | 313 | 13 | 326 | intergenic | - |
| 227 | - | 1111692-1116766 | 5075 | 1088463-1093524 | 5062 | - | - | - | - |
| 228 | INDEL_LONG | 1116767-1119250 | 2484 | - | - | 8 | 2492 | in_gene | ancestral: C23 |
| 229 | - | 1119251-1120291 | 1041 | 1093517-1094557 | 1041 | - | - | - | - |
| 230 | REPLACEMENT_LONG_MEDIUM | 1120292-1121687 | 1396 | 1094558-1096913 | 2356 | - | - | N/A | - |
| 231 | - | 1121688-1126188 | 4501 | 1096914-1101406 | 4493 | - | - | - | - |
| 232 | INDEL_LONG | - | - | 1101407-1105429 | 4023 | 6 | 4029 | intergenic | - |
| 233 | - | 1126183-1143147 | 16965 | 1105430-1122406 | 16977 | - | - | - | - |
| 234 | INDEL_MISC | 1143148-1143222 | 75 | 1122407-1123177 | 771 | - | - | intergenic | - |
| 235 | - | 1143223-1151044 | 7822 | 1123178-1131016 | 7839 | - | - | - | - |
| 236 | INDEL_SHORT | - | - | 1131017-1131052 | 36 | 1 | 37 | intergenic | - |
| 237 | - | 1151044-1158374 | 7331 | 1131053-1138384 | 7332 | - | - | - | - |
| 238 | INSERT_REPEAT | 1158375-1158427 | 53 | 1138385-1138389 | 5 | - | - | intergenic | ISHwa17(core_deletion) |
| 239 | - | 1158428-1179370 | 20943 | 1138390-1159337 | 20948 | - | - | - | - |
| 240 | INSERT_REPEAT | 1179371-1179375 | 5 | 1159338-1159888 | 551 | - | - | intergenic | HqIRS56 |
| 241 | - | 1179376-1189255 | 9880 | 1159889-1169757 | 9869 | - | - | - | - |
| 242 | REPLACEMENT_MEDIUM_MEDIUM | 1189256-1189688 | 433 | 1169758-1170029 | 272 | - | - | N/A | - |
| 243 | - | 1189689-1190161 | 473 | 1170030-1170502 | 473 | - | - | - | - |
| 244 | REPLACEMENT_LONG_MEDIUM | 1190162-1191671 | 1510 | 1170503-1170736 | 234 | - | - | N/A | - |
| 245 | - | 1191672-1202748 | 11077 | 1170737-1181811 | 11075 | - | - | - | - |
| 246 | INSERT_TRANSPOSON | 1202749-1204033 | 1285 | - | - | 8 | 1293 | intergenic | ISHwa4 |
| 247 | - | 1204034-1216810 | 12777 | 1181804-1194580 | 12777 | - | - | - | - |
| 248 | REPLACEMENT_LONG_MEDIUM | 1216811-1218042 | 1232 | 1194581-1210472 | 15892 | - | - | N/A | - |
| 249 | - | 1218043-1233992 | 15950 | 1210473-1226424 | 15952 | - | - | - | - |
| 250 | INDEL_POLYREPEAT | 1233993-1234062 | 70 | 1226425-1226469 | 45 | - | - | intergenic | - |
| 251 | - | 1234063-1235104 | 1042 | 1226470-1227510 | 1041 | - | - | - | - |
| 252 | INSERT_REPEAT | 1235105-1235108 | 4 | 1227511-1227562 | 52 | - | - | intergenic | ISHwa23(core_deletion) |
| 253 | - | 1235109-1243352 | 8244 | 1227563-1235806 | 8244 | - | - | - | - |
| 254 | INDEL_LONG | 1243353-1243355 | 3 | 1235807-1244291 | 8485 | - | - | in_gene | ancestral: C23 |
| 255 | - | 1243356-1247749 | 4394 | 1244292-1248686 | 4395 | - | - | - | - |
| 256 | INSERT_TRANSPOSON | - | - | 1248687-1249623 | 937 | 8 | 945 | intergenic | ISHwa1 |
| 257 | - | 1247742-1258351 | 10610 | 1249624-1260233 | 10610 | - | - | - | - |
| 258 | INDEL_POLYREPEAT | 1258352-1258397 | 46 | 1260234-1260319 | 86 | - | - | intergenic | - |
| 259 | - | 1258398-1259612 | 1215 | 1260320-1261534 | 1215 | - | - | - | - |
| 260 | INDEL_POLYREPEAT | 1259613-1259705 | 93 | 1261535-1261603 | 69 | - | - | in_gene | - |
| 261 | - | 1259706-1265494 | 5789 | 1261604-1267399 | 5796 | - | - | - | - |
| 262 | INSERT_TRANSPOSON | 1265495-1266779 | 1285 | - | - | 7 | 1292 | in_gene | ISHwa4 |
| 263 | - | 1266780-1268550 | 1771 | 1267393-1269163 | 1771 | - | - | - | - |
| 264 | INDEL_LONG | 1268551-1284680 | 16130 | - | - | 10 | 16141 | in_gene | DV6,GI2; ancestral: C23 |
| 265 | - | 1284681-1286414 | 1734 | 1269154-1270887 | 1734 | - | - | - | - |
| 266 | INDEL_LONG | 1286415-1288891 | 2477 | - | - | 4 | 2481 | in_gene | DV6,GI2; ancestral: C23 |
| 267 | - | 1288892-1290119 | 1228 | 1270884-1272135 | 1252 | - | - | - | - |
| 268 | INDEL_MEDIUM | - | - | 1272136-1273387 | 1252 | - | - | in_transposon | DV6,GI2; ancestral: HBSQ001 |
| 269 | - | 1290120-1290400 | 281 | 1273388-1273668 | 281 | - | - | - | - |
| 270 | INDEL_LONG | - | - | 1273669-1286183 | 12515 | - | - | intergenic | DV6,GI2 |
| 271 | - | 1290401-1295922 | 5522 | 1286184-1291705 | 5522 | - | - | - | - |
| 272 | REPLACEMENT_LONG_LONG | 1295923-1297449 | 1527 | 1291706-1293551 | 1846 | - | - | N/A | DV6,GI2 |
| 273 | - | 1297450-1298382 | 933 | 1293552-1294487 | 936 | - | - | - | - |
| 274 | REPLACEMENT_LONG_LONG | 1298383-1318245 | 19863 | 1294488-1310935 | 16448 | - | - | N/A | DV6,GI2 |
| 275 | - | 1318246-1330475 | 12230 | 1310936-1323165 | 12230 | - | - | - | - |
| 276 | REPLACEMENT_SHORT_SHORT | 1330476-1330505 | 30 | 1323166-1323207 | 42 | - | - | in_gene | DV6,GI2; reading frame conserved |
| 277 | - | 1330506-1331763 | 1258 | 1323208-1324457 | 1250 | - | - | - | - |
| 278 | INDEL_MEDIUM | 1331764-1332370 | 607 | - | - | 734 | 1341 | intergenic | DV6,GI2 |
| 279 | - | 1332371-1339166 | 6796 | 1323724-1330512 | 6789 | - | - | - | - |
| 280 | INDEL_SHORT | 1339167-1339186 | 20 | - | - | 6 | 26 | in_gene | DV6,GI2 |
| 281 | - | 1339187-1347394 | 8208 | 1330507-1338717 | 8211 | - | - | - | - |
| 282 | INDEL_SHORT | - | - | 1338718-1338760 | 43 | 13 | 56 | in_gene | DV6,GI2 |
| 283 | - | 1347382-1347429 | 48 | 1338761-1338806 | 46 | - | - | - | - |
| 284 | REPLACEMENT_LONG_LONG | 1347430-1356005 | 8576 | 1338807-1342481 | 3675 | - | - | N/A | DV6,GI2; ancestral: C23 |
| 285 | - | 1356006-1359860 | 3855 | 1342482-1346354 | 3873 | - | - | - | - |
| 286 | INSERT_REPEAT | - | - | 1346355-1346464 | 110 | 1 | 111 | in_gene | DV6,GI2; HqIRS55 |
| 287 | - | 1359860-1367405 | 7546 | 1346465-1354019 | 7555 | - | - | - | - |
| 288 | REPLACEMENT_LONG_MEDIUM | 1367406-1368737 | 1332 | 1354020-1358333 | 4314 | - | - | N/A | DV6,GI2 |
| 289 | - | 1368738-1381167 | 12430 | 1358334-1370761 | 12428 | - | - | - | - |
| 290 | INDEL_LONG | - | - | 1370762-1373427 | 2666 | 139 | 2805 | intergenic | DV6,GI2 |
| 291 | - | 1381029-1383732 | 2704 | 1373428-1376126 | 2699 | - | - | - | - |
| 292 | INDEL_MEDIUM | 1383733-1383892 | 160 | - | - | 5 | 165 | intergenic | DV6,GI2; ancestral: HBSQ001 |
| 293 | - | 1383893-1391075 | 7183 | 1376122-1383310 | 7189 | - | - | - | - |
| 294 | REPLACEMENT_LONG_LONG | 1391076-1406673 | 15598 | 1383311-1388591 | 5281 | - | - | N/A | DV6,GI2 |
| 295 | - | 1406674-1407184 | 511 | 1388592-1389102 | 511 | - | - | - | - |
| 296 | REPLACEMENT_LONG_MEDIUM | 1407185-1410242 | 3058 | 1389103-1390042 | 940 | - | - | N/A | DV6,GI2; ancestral: HBSQ001 |
| 297 | - | 1410243-1418838 | 8596 | 1390043-1398631 | 8589 | - | - | - | - |
| 298 | INDEL_LONG | - | - | 1398632-1403402 | 4771 | 10 | 4781 | in_gene | DV6,GI2; ancestral: HBSQ001 |
| 299 | - | 1418829-1422579 | 3751 | 1403403-1407153 | 3751 | - | - | - | - |
| 300 | INDEL_POLYREPEAT | 1422580-1422621 | 42 | 1407154-1407171 | 18 | - | - | intergenic | DV6,GI2 |
| 301 | - | 1422622-1423700 | 1079 | 1407172-1408250 | 1079 | - | - | - | - |
| 302 | REPLACEMENT_LONG_LONG | 1423701-1428489 | 4789 | 1408251-1425425 | 17175 | - | - | N/A | DV6,GI2; ancestral: C23 |
| 303 | - | 1428490-1432543 | 4054 | 1425426-1429466 | 4041 | - | - | - | - |
| 304 | REPLACEMENT_LONG_MEDIUM | 1432544-1446571 | 14028 | 1429467-1430078 | 612 | - | - | N/A | DV6,GI2; ancestral: HBSQ001 |
| 305 | - | 1446572-1460849 | 14278 | 1430079-1444354 | 14276 | - | - | - | - |
| 306 | INDEL_POLYREPEAT | 1460850-1460897 | 48 | 1444355-1444366 | 12 | - | - | intergenic | DV6,GI2 |
| 307 | - | 1460898-1464909 | 4012 | 1444367-1448383 | 4017 | - | - | - | - |
| 308 | INSERT_REPEAT | 1464910-1464913 | 4 | 1448384-1448945 | 562 | - | - | in_gene | DV6,GI2; sequence not affected; HqIRS57 |
| 309 | - | 1464914-1467774 | 2861 | 1448946-1451804 | 2859 | - | - | - | - |
| 310 | INSERT_TRANSPOSON | 1467775-1469283 | 1509 | - | - | 5 | 1514 | intergenic | DV6,GI2; ISHwa12 |
| 311 | - | 1469284-1469923 | 640 | 1451800-1452439 | 640 | - | - | - | - |
| 312 | INSERT_MITE | 1469924-1470072 | 149 | - | - | 6 | 155 | intergenic | DV6,GI2; HqIRS37 |
| 313 | - | 1470073-1471915 | 1843 | 1452434-1454275 | 1842 | - | - | - | - |
| 314 | INSERT_REPEAT | 1471916-1472326 | 411 | 1454276-1454284 | 9 | - | - | intergenic | DV6,GI2; HqIRS44 |
| 315 | - | 1472327-1478269 | 5943 | 1454285-1460233 | 5949 | - | - | - | - |
| 316 | INSERT_REPEAT | 1478270-1478378 | 109 | - | - | 1 | 110 | intergenic | HqIRS55 |
| 317 | - | 1478379-1490889 | 12511 | 1460233-1472737 | 12505 | - | - | - | - |
| 318 | INSERT_MITE | - | - | 1472738-1472901 | 164 | 8 | 172 | intergenic | HqIRS36 |
| 319 | - | 1490882-1493952 | 3071 | 1472902-1475972 | 3071 | - | - | - | - |
| 320 | REPLACEMENT_SHORT_SHORT | 1493953-1493976 | 24 | 1475973-1475985 | 13 | - | - | in_gene | - |
| 321 | - | 1493977-1494105 | 129 | 1475986-1476114 | 129 | - | - | - | - |
| 322 | REPLACEMENT_SHORT_SHORT | 1494106-1494120 | 15 | 1476115-1476142 | 28 | - | - | in_gene | - |
| 323 | - | 1494121-1509430 | 15310 | 1476143-1491464 | 15322 | - | - | - | - |
| 324 | REPLACEMENT_SHORT_SHORT | 1509431-1509550 | 120 | 1491465-1491539 | 75 | - | - | in_gene | reading frame conserved |
| 325 | - | 1509551-1509634 | 84 | 1491540-1491623 | 84 | - | - | - | - |
| 326 | REPLACEMENT_MEDIUM_MEDIUM | 1509635-1509909 | 275 | 1491624-1492101 | 478 | - | - | in_gene | sequence not affected |
| 327 | - | 1509910-1517964 | 8055 | 1492102-1500174 | 8073 | - | - | - | - |
| 328 | INSERT_REPEAT | 1517965-1518044 | 80 | - | - | 1 | 81 | intergenic | HqIRS41 |
| 329 | - | 1518045-1528300 | 10256 | 1500174-1510439 | 10266 | - | - | - | - |
| 330 | REPLACEMENT_LONG_SHORT | 1528301-1528347 | 47 | 1510440-1516181 | 5742 | - | - | N/A | - |
| 331 | - | 1528348-1531839 | 3492 | 1516182-1519670 | 3489 | - | - | - | - |
| 332 | INDEL_SHORT | 1531840-1531907 | 68 | - | - | - | - | in_gene | ancestral: HBSQ001 |
| 333 | - | 1531908-1532321 | 414 | 1519671-1520084 | 414 | - | - | - | - |
| 334 | INDEL_MEDIUM | - | - | 1520085-1520325 | 241 | - | - | intergenic | - |
| 335 | - | 1532322-1534018 | 1697 | 1520326-1522021 | 1696 | - | - | - | - |
| 336 | INDEL_SHORT | - | - | 1522022-1522137 | 116 | 13 | 129 | intergenic | ancestral: HBSQ001 |
| 337 | - | 1534006-1537080 | 3075 | 1522138-1525211 | 3074 | - | - | - | - |
| 338 | INSERT_TRANSPOSON | 1537081-1538974 | 1894 | - | - | 8 | 1902 | intergenic | ISHwa10 |
| 339 | - | 1538975-1542071 | 3097 | 1525204-1528307 | 3104 | - | - | - | - |
| 340 | INDEL_SHORT | - | - | 1528308-1528348 | 41 | - | - | in_transposon | - |
| 341 | - | 1542072-1546231 | 4160 | 1528349-1532495 | 4147 | - | - | - | - |
| 342 | INDEL_MEDIUM | - | - | 1532496-1533471 | 976 | 9 | 985 | in_transposon | ancestral: HBSQ001 |
| 343 | - | 1546223-1547129 | 907 | 1533472-1534379 | 908 | - | - | - | - |
| 344 | INSERT_REPEAT | 1547130-1547133 | 4 | 1534380-1534431 | 52 | - | - | in_gene | ISHwa23(core_deletion) |
| 345 | - | 1547134-1549246 | 2113 | 1534432-1536542 | 2111 | - | - | - | - |
| 346 | INSERT_REPEAT | 1549247-1549788 | 542 | 1536543-1536546 | 4 | - | - | intergenic | HqIRS56 |
| 347 | - | 1549789-1562968 | 13180 | 1536547-1549726 | 13180 | - | - | - | - |
| 348 | INSERT_REPEAT | 1562969-1562972 | 4 | 1549727-1550263 | 537 | - | - | intergenic | HqIRS56 |
| 349 | - | 1562973-1576673 | 13701 | 1550264-1563955 | 13692 | - | - | - | - |
| 350 | INDEL_MEDIUM | - | - | 1563956-1565371 | 1416 | 11 | 1427 | in_gene | - |
| 351 | - | 1576663-1576898 | 236 | 1565372-1565607 | 236 | - | - | - | - |
| 352 | INDEL_SHORT | 1576899-1576945 | 47 | - | - | - | - | in_gene | - |
| 353 | - | 1576946-1577049 | 104 | 1565608-1565711 | 104 | - | - | - | - |
| 354 | INDEL_LONG | 1577050-1577052 | 3 | 1565712-1568733 | 3022 | - | - | in_gene | ancestral: HBSQ001 |
| 355 | - | 1577053-1577333 | 281 | 1568734-1569014 | 281 | - | - | - | - |
| 356 | INSERT_TRANSPOSON | 1577334-1578842 | 1509 | - | - | 5 | 1514 | intergenic | ISHwa12 |
| 357 | - | 1578843-1586502 | 7660 | 1569010-1576677 | 7668 | - | - | - | - |
| 358 | INDEL_SHORT | - | - | 1576678-1576702 | 25 | 5 | 30 | intergenic | - |
| 359 | - | 1586498-1592465 | 5968 | 1576703-1582683 | 5981 | - | - | - | - |
| 360 | INSERT_TRANSPOSON | 1592466-1594126 | 1661 | - | - | 10 | 1671 | intergenic | ISHwa2 |
| 361 | - | 1594127-1600465 | 6339 | 1582674-1589011 | 6338 | - | - | - | - |
| 362 | INDEL_POLYREPEAT | 1600466-1600495 | 30 | 1589012-1589059 | 48 | - | - | intergenic | - |
| 363 | - | 1600496-1609341 | 8846 | 1589060-1597915 | 8856 | - | - | - | - |
| 364 | INSERT_REPEAT | 1609342-1609347 | 6 | 1597916-1598029 | 114 | - | - | intergenic | HqIRS43 |
| 365 | - | 1609348-1611113 | 1766 | 1598030-1599785 | 1756 | - | - | - | - |
| 366 | INSERT_REPEAT | 1611114-1611270 | 157 | - | - | - | - | intergenic | HqIRS56(core_deletion,targeted) |
| 367 | - | 1611271-1612886 | 1616 | 1599786-1601409 | 1624 | - | - | - | - |
| 368 | INDEL_POLYREPEAT | 1612887-1612916 | 30 | 1601410-1601493 | 84 | - | - | in_gene | reading frame conserved |
| 369 | - | 1612917-1615792 | 2876 | 1601494-1604369 | 2876 | - | - | - | - |
| 370 | INDEL_LONG | 1615793-1635538 | 19746 | - | - | 22 | 19768 | in_tRNA | DV7; ancestral: HBSQ001 |
| 371 | - | 1635539-1635668 | 130 | 1604348-1604477 | 130 | - | - | - | - |
| 372 | INSERT_REPEAT | 1635669-1635672 | 4 | 1604478-1605112 | 635 | - | - | intergenic | HqIRS54(targeted) |
| 373 | - | 1635673-1636266 | 594 | 1605113-1605706 | 594 | - | - | - | - |
| 374 | INDEL_POLYREPEAT | 1636267-1636278 | 12 | 1605707-1605730 | 24 | - | - | intergenic | - |
| 375 | - | 1636279-1650470 | 14192 | 1605731-1619952 | 14222 | - | - | - | - |
| 376 | INSERT_REPEAT | 1650471-1650554 | 84 | 1619953-1619956 | 4 | - | - | intergenic | HqIRS41 |
| 377 | - | 1650555-1657072 | 6518 | 1619957-1626486 | 6530 | - | - | - | - |
| 378 | INDEL_SHORT | 1657073-1657148 | 76 | - | - | 8 | 84 | in_gene | reading frame conserved |
| 379 | - | 1657149-1659113 | 1965 | 1626479-1628444 | 1966 | - | - | - | - |
| 380 | INSERT_REPEAT | 1659114-1659506 | 393 | 1628445-1628449 | 5 | - | - | intergenic | HqIRS47 |
| 381 | - | 1659507-1668771 | 9265 | 1628450-1637733 | 9284 | - | - | - | - |
| 382 | INSERT_REPEAT | 1668772-1668775 | 4 | 1637734-1638216 | 483 | - | - | intergenic | HqIRS54(targeted) |
| 383 | - | 1668776-1677878 | 9103 | 1638217-1647306 | 9090 | - | - | - | - |
| 384 | INDEL_POLYREPEAT | 1677879-1677902 | 24 | 1647307-1647360 | 54 | - | - | in_gene | reading frame conserved |
| 385 | - | 1677903-1685345 | 7443 | 1647361-1654826 | 7466 | - | - | - | - |
| 386 | INSERT_TRANSPOSON | 1685346-1686630 | 1285 | - | - | 7 | 1292 | intergenic | ISHwa4 |
| 387 | - | 1686631-1695968 | 9338 | 1654820-1664154 | 9335 | - | - | - | - |
| 388 | INDEL_LONG | 1695969-1730200 | 34232 | - | - | 6 | 34238 | in_gene | DV8; ancestral: C23 |
| 389 | - | 1730201-1745273 | 15073 | 1664149-1679215 | 15067 | - | - | - | - |
| 390 | INSERT_REPEAT | - | - | 1679216-1679323 | 108 | - | - | in_repeat | HqIRS43 |
| 391 | - | 1745274-1769602 | 24329 | 1679324-1703660 | 24337 | - | - | - | - |
| 392 | INDEL_MEDIUM | - | - | 1703661-1704004 | 344 | 4 | 348 | intergenic | - |
| 393 | - | 1769599-1781981 | 12383 | 1704005-1716393 | 12389 | - | - | - | - |
| 394 | INSERT_REPEAT | 1781982-1781985 | 4 | 1716394-1716768 | 375 | - | - | intergenic | HqIRS54 |
| 395 | - | 1781986-1793125 | 11140 | 1716769-1727911 | 11143 | - | - | - | - |
| 396 | INSERT_REPEAT | - | - | 1727912-1728021 | 110 | 1 | 111 | intergenic | HqIRS55 |
| 397 | - | 1793125-1795067 | 1943 | 1728022-1729957 | 1936 | - | - | - | - |
| 398 | INSERT_TRANSPOSON | 1795068-1795074 | 7 | 1729958-1731996 | 2039 | - | - | in_transposon | ISHwa17 |
| 399 | - | 1795075-1796070 | 996 | 1731997-1732993 | 997 | - | - | - | - |
| 400 | INDEL_MEDIUM | - | - | 1732994-1733222 | 229 | 8 | 237 | intergenic | - |
| 401 | - | 1796063-1806085 | 10023 | 1733223-1743258 | 10036 | - | - | - | - |
| 402 | INSERT_REPEAT | - | - | 1743259-1743314 | 56 | - | - | in_gene | sequence not affected; HqIRS82 |
| 403 | - | 1806086-1811788 | 5703 | 1743315-1749015 | 5701 | - | - | - | - |
| 404 | REPLACEMENT_SHORT_SHORT | 1811789-1811843 | 55 | 1749016-1749022 | 7 | - | - | intergenic | - |
| 405 | - | 1811844-1817866 | 6023 | 1749023-1755061 | 6039 | - | - | - | - |
| 406 | INDEL_MEDIUM | - | - | 1755062-1755324 | 263 | 1 | 264 | in_repeat | - |
| 407 | - | 1817866-1825355 | 7490 | 1755325-1762833 | 7509 | - | - | - | - |
| 408 | INDEL_POLYREPEAT | 1825356-1825369 | 14 | 1762834-1762859 | 26 | - | - | intergenic | - |
| 409 | - | 1825370-1825431 | 62 | 1762860-1762921 | 62 | - | - | - | - |
| 410 | INDEL_POLYREPEAT | 1825432-1825452 | 21 | 1762922-1762928 | 7 | - | - | intergenic | - |
| 411 | - | 1825453-1831306 | 5854 | 1762929-1768784 | 5856 | - | - | - | - |
| 412 | REPLACEMENT_SHORT_SHORT | 1831307-1831338 | 32 | 1768785-1768828 | 44 | - | - | intergenic | - |
| 413 | - | 1831339-1831379 | 41 | 1768829-1768869 | 41 | - | - | - | - |
| 414 | REPLACEMENT_SHORT_SHORT | 1831380-1831398 | 19 | 1768870-1768906 | 37 | - | - | intergenic | - |
| 415 | - | 1831399-1844130 | 12732 | 1768907-1781660 | 12754 | - | - | - | - |
| 416 | INDEL_SHORT | 1844131-1844176 | 46 | - | - | 1 | 47 | intergenic | - |
| 417 | - | 1844177-1844264 | 88 | 1781660-1781747 | 88 | - | - | - | - |
| 418 | INSERT_REPEAT | 1844265-1844374 | 110 | - | - | 1 | 111 | intergenic | HqIRS55 |
| 419 | - | 1844375-1844820 | 446 | 1781747-1782206 | 460 | - | - | - | - |
| 420 | INDEL_MEDIUM | - | - | 1782207-1783646 | 1440 | - | - | in_gene | ancestral: HBSQ001 |
| 421 | - | 1844821-1847109 | 2289 | 1783647-1785925 | 2279 | - | - | - | - |
| 422 | INDEL_SHORT | - | - | 1785926-1785938 | 13 | 12 | 25 | in_gene | - |
| 423 | - | 1847098-1848793 | 1696 | 1785939-1787634 | 1696 | - | - | - | - |
| 424 | INDEL_MEDIUM | - | - | 1787635-1788393 | 759 | 1 | 760 | in_transposon | - |
| 425 | - | 1848793-1858633 | 9841 | 1788394-1798223 | 9830 | - | - | - | - |
| 426 | REPLACEMENT_SHORT_SHORT | 1858634-1858679 | 46 | 1798224-1798264 | 41 | - | - | in_repeat | - |
| 427 | - | 1858680-1868538 | 9859 | 1798265-1808129 | 9865 | - | - | - | - |
| 428 | DELETE_REPEATCORE | - | - | 1808130-1809558 | 1429 | 19 | 1448 | in_repeat | ISHwa23 |
| 429 | - | 1868520-1872955 | 4436 | 1809559-1813994 | 4436 | - | - | - | - |
| 430 | INSERT_TRANSPOSON | 1872956-1873900 | 945 | - | - | 8 | 953 | in_gene | HqIRS11 |
| 431 | - | 1873901-1875929 | 2029 | 1813987-1816020 | 2034 | - | - | - | - |
| 432 | INSERT_TRANSPOSON | 1875930-1878732 | 2803 | - | - | 8 | 2811 | intergenic | ISHwa9(targeted) |
| 433 | - | 1878733-1880962 | 2230 | 1816013-1818243 | 2231 | - | - | - | - |
| 434 | INSERT_MITE | 1880963-1881221 | 259 | - | - | 10 | 269 | intergenic | HqIRS35 |
| 435 | - | 1881222-1882111 | 890 | 1818234-1819110 | 877 | - | - | - | - |
| 436 | INDEL_SHORT | 1882112-1882122 | 11 | - | - | 10 | 21 | intergenic | - |
| 437 | - | 1882123-1882160 | 38 | 1819101-1819138 | 38 | - | - | - | - |
| 438 | REPLACEMENT_MEDIUM_MEDIUM | 1882161-1883175 | 1015 | 1819139-1819682 | 544 | - | - | N/A | - |
| 439 | - | 1883176-1885346 | 2171 | 1819683-1821854 | 2172 | - | - | - | - |
| 440 | INSERT_REPEAT | 1885347-1885350 | 4 | 1821855-1822511 | 657 | - | - | intergenic | HqIRS56(targeted) |
| 441 | - | 1885351-1885639 | 289 | 1822512-1822802 | 291 | - | - | - | - |
| 442 | INSERT_REPEAT | 1885640-1885748 | 109 | - | - | 1 | 110 | in_repeat | HqIRS43 |
| 443 | - | 1885749-1905248 | 19500 | 1822802-1842310 | 19509 | - | - | - | - |
| 444 | INDEL_POLYREPEAT | 1905249-1905290 | 42 | 1842311-1842334 | 24 | - | - | in_repeat | - |
| 445 | - | 1905291-1909101 | 3811 | 1842335-1846136 | 3802 | - | - | - | - |
| 446 | INSERT_MITE | - | - | 1846137-1846255 | 119 | 9 | 128 | intergenic | HqIRS39 |
| 447 | - | 1909093-1911315 | 2223 | 1846256-1848478 | 2223 | - | - | - | - |
| 448 | INSERT_TRANSPOSON | 1911316-1912811 | 1496 | - | - | - | - | intergenic | ISHwa23 |
| 449 | - | 1912812-1914635 | 1824 | 1848479-1850303 | 1825 | - | - | - | - |
| 450 | INDEL_SHORT | - | - | 1850304-1850347 | 44 | - | - | intergenic | - |
| 451 | - | 1914636-1914667 | 32 | 1850348-1850379 | 32 | - | - | - | - |
| 452 | INSERT_REPEAT | 1914668-1914720 | 53 | 1850380-1850384 | 5 | - | - | intergenic | ISHwa17(core_deletion) |
| 453 | - | 1914721-1922007 | 7287 | 1850385-1857662 | 7278 | - | - | - | - |
| 454 | INSERT_MITE | 1922008-1922172 | 165 | - | - | 8 | 173 | intergenic | HqIRS36 |
| 455 | - | 1922173-1922376 | 204 | 1857655-1857858 | 204 | - | - | - | - |
| 456 | INSERT_REPEAT | - | - | 1857859-1857968 | 110 | 1 | 111 | intergenic | HqIRS55 |
| 457 | - | 1922376-1944910 | 22535 | 1857969-1880503 | 22535 | - | - | - | - |
| 458 | INDEL_LONG | - | - | 1880504-1884457 | 3954 | - | - | intergenic | - |
| 459 | - | 1944911-1944924 | 14 | 1884458-1884471 | 14 | - | - | - | - |
| 460 | INSERT_MITE | - | - | 1884472-1884731 | 260 | 10 | 270 | intergenic | HqIRS35 |
| 461 | - | 1944915-1949483 | 4569 | 1884732-1889300 | 4569 | - | - | - | - |
| 462 | REPLACEMENT_SHORT_SHORT | 1949484-1949548 | 65 | 1889301-1889366 | 66 | - | - | intergenic | - |
| 463 | - | 1949549-1949726 | 178 | 1889367-1889543 | 177 | - | - | - | - |
| 464 | REPLACEMENT_SHORT_SHORT | 1949727-1949767 | 41 | 1889544-1889621 | 78 | - | - | intergenic | - |
| 465 | - | 1949768-1952400 | 2633 | 1889622-1892267 | 2646 | - | - | - | - |
| 466 | INDEL_LONG | 1952401-1959157 | 6757 | - | - | 17 | 6774 | in_gene | ancestral: C23 |
| 467 | - | 1959158-1990657 | 31500 | 1892251-1923752 | 31502 | - | - | - | - |
| 468 | INDEL_MEDIUM | 1990658-1990978 | 321 | - | - | - | - | in_transposon | ancestral: C23 |
| 469 | - | 1990979-1992330 | 1352 | 1923753-1925110 | 1358 | - | - | - | - |
| 470 | INSERT_TRANSPOSON | 1992331-1993840 | 1510 | - | - | 8 | 1518 | intergenic | ISHwa8 |
| 471 | - | 1993841-2019670 | 25830 | 1925103-1950933 | 25831 | - | - | - | - |
| 472 | INSERT_TRANSPOSON | 2019671-2021179 | 1509 | - | - | 5 | 1514 | intergenic | ISHwa12 |
| 473 | - | 2021180-2023156 | 1977 | 1950929-1952905 | 1977 | - | - | - | - |
| 474 | INSERT_REPEAT | 2023157-2023266 | 110 | - | - | 1 | 111 | intergenic | HqIRS55 |
| 475 | - | 2023267-2024694 | 1428 | 1952905-1954332 | 1428 | - | - | - | - |
| 476 | INSERT_REPEAT | 2024695-2024699 | 5 | 1954333-1954385 | 53 | - | - | intergenic | ISHwa23(core_deletion) |
| 477 | - | 2024700-2031284 | 6585 | 1954386-1960964 | 6579 | - | - | - | - |
| 478 | INSERT_REPEAT | 2031285-2031690 | 406 | 1960965-1960969 | 5 | - | - | intergenic | HqIRS44 |
| 479 | - | 2031691-2046793 | 15103 | 1960970-1976049 | 15080 | - | - | - | - |
| 480 | REPLACEMENT_LONG_LONG | 2046794-2069450 | 22657 | 1976050-1989452 | 13403 | - | - | N/A | DV9; ancestral: C23 |
| 481 | - | 2069451-2071873 | 2423 | 1989453-1991877 | 2425 | - | - | - | - |
| 482 | REPLACEMENT_SHORT_SHORT | 2071874-2071908 | 35 | 1991878-1991924 | 47 | - | - | intergenic | - |
| 483 | - | 2071909-2089451 | 17543 | 1991925-2009460 | 17536 | - | - | - | - |
| 484 | INDEL_POLYREPEAT | 2089452-2089499 | 48 | 2009461-2009532 | 72 | - | - | in_gene | reading frame conserved |
| 485 | - | 2089500-2096386 | 6887 | 2009533-2016419 | 6887 | - | - | - | - |
| 486 | INDEL_POLYREPEAT | 2096387-2096444 | 58 | 2016420-2016441 | 22 | - | - | in_repeat | - |
| 487 | - | 2096445-2105726 | 9282 | 2016442-2025728 | 9287 | - | - | - | - |
| 488 | INSERT_TRANSPOSON | 2105727-2105730 | 4 | 2025729-2027228 | 1500 | - | - | intergenic | ISHwa23 |
| 489 | - | 2105731-2110319 | 4589 | 2027229-2031820 | 4592 | - | - | - | - |
| 490 | INDEL_MEDIUM | - | - | 2031821-2032056 | 236 | 2 | 238 | intergenic | - |
| 491 | - | 2110318-2118200 | 7883 | 2032057-2039916 | 7860 | - | - | - | - |
| 492 | INSERT_REPEAT | 2118201-2118205 | 5 | 2039917-2040482 | 566 | - | - | intergenic | HqIRS71 |
| 493 | - | 2118206-2122552 | 4347 | 2040483-2044824 | 4342 | - | - | - | - |
| 494 | INDEL_POLYREPEAT | 2122553-2122598 | 46 | 2044825-2044948 | 124 | - | - | in_repeat | - |
| 495 | - | 2122599-2122862 | 264 | 2044949-2045212 | 264 | - | - | - | - |
| 496 | INDEL_POLYREPEAT | 2122863-2122886 | 24 | 2045213-2045290 | 78 | - | - | in_repeat | - |
| 497 | - | 2122887-2128469 | 5583 | 2045291-2050857 | 5567 | - | - | - | - |
| 498 | INDEL_MEDIUM | - | - | 2050858-2051052 | 195 | 3 | 198 | in_transposons | ancestral: HBSQ |
| 499 | - | 2128467-2131826 | 3360 | 2051053-2054413 | 3361 | - | - | - | - |
| 500 | INSERT_REPEAT | - | - | 2054414-2054521 | 108 | - | - | in_repeat | HqIRS42 |
| 501 | - | 2131827-2146712 | 14886 | 2054522-2069384 | 14863 | - | - | - | - |
| 502 | INDEL_MEDIUM | 2146713-2146876 | 164 | - | - | 5 | 169 | in_transposon | ancestral: C23 |
| 503 | - | 2146877-2154831 | 7955 | 2069380-2077339 | 7960 | - | - | - | - |
| 504 | INSERT_REPEAT | 2154832-2155388 | 557 | 2077340-2077344 | 5 | - | - | intergenic | HqIRS56 |
| 505 | - | 2155389-2158947 | 3559 | 2077345-2080902 | 3558 | - | - | - | - |
| 506 | INSERT_MITE | 2158948-2159066 | 119 | - | - | 8 | 127 | intergenic | HqIRS39 |
| 507 | - | 2159067-2161760 | 2694 | 2080895-2083588 | 2694 | - | - | - | - |
| 508 | REPLACEMENT_LONG_MEDIUM | 2161761-2174095 | 12335 | 2083589-2084272 | 684 | - | - | N/A | - |
| 509 | - | 2174096-2188250 | 14155 | 2084273-2098450 | 14178 | - | - | - | - |
| 510 | INDEL_SHORT | 2188251-2188330 | 80 | 2098451-2098454 | 4 | - | - | in_gene | sequence not affected |
| 511 | - | 2188331-2190167 | 1837 | 2098455-2100291 | 1837 | - | - | - | - |
| 512 | INSERT_REPEAT | 2190168-2190171 | 4 | 2100292-2100343 | 52 | - | - | in_gene | sequence not affected; ISHwa23(core_deletion) |
| 513 | - | 2190172-2202311 | 12140 | 2100344-2112495 | 12152 | - | - | - | - |
| 514 | INSERT_MITE | - | - | 2112496-2112754 | 259 | 10 | 269 | in_transposon | HqIRS35 |
| 515 | - | 2202302-2209291 | 6990 | 2112755-2119743 | 6989 | - | - | - | - |
| 516 | INSERT_TRANSPOSON | 2209292-2212797 | 3506 | 2119744-2119747 | 4 | - | - | intergenic | ISHwa21(targeted) |
| 517 | - | 2212798-2214313 | 1516 | 2119748-2121268 | 1521 | - | - | - | - |
| 518 | INDEL_SHORT | - | - | 2121269-2121284 | 16 | 4 | 20 | intergenic | - |
| 519 | - | 2214310-2214384 | 75 | 2121285-2121359 | 75 | - | - | - | - |
| 520 | INDEL_MEDIUM | 2214385-2214671 | 287 | - | - | 2 | 289 | intergenic | - |
| 521 | - | 2214672-2220231 | 5560 | 2121358-2126932 | 5575 | - | - | - | - |
| 522 | INDEL_SHORT | 2220232-2220254 | 23 | 2126933-2126936 | 4 | - | - | intergenic | - |
| 523 | - | 2220255-2220322 | 68 | 2126937-2127005 | 69 | - | - | - | - |
| 524 | INDEL_LONG | 2220323-2225758 | 5436 | - | - | 15 | 5451 | in_gene | sequence not affected |
| 525 | - | 2225759-2241773 | 16015 | 2126991-2142973 | 15983 | - | - | - | - |
| 526 | INSERT_TRANSPOSON | 2241774-2243283 | 1510 | - | - | 8 | 1518 | intergenic | ISHwa8 |
| 527 | - | 2243284-2247248 | 3965 | 2142966-2146931 | 3966 | - | - | - | - |
| 528 | INSERT_REPEAT | 2247249-2247409 | 161 | 2146932-2146935 | 4 | - | - | intergenic | HqIRS56 |
| 529 | - | 2247410-2247533 | 124 | 2146936-2147059 | 124 | - | - | - | - |
| 530 | INSERT_TRANSPOSON | 2247534-2248818 | 1285 | - | - | 7 | 1292 | in_repeat | ISHwa4 |
| 531 | - | 2248819-2254863 | 6045 | 2147053-2153084 | 6032 | - | - | - | - |
| 532 | INSERT_TRANSPOSON | 2254864-2257119 | 2256 | - | - | 11 | 2267 | in_repeat | ISHwa5 |
| 533 | - | 2257120-2287217 | 30098 | 2153074-2183176 | 30103 | - | - | - | - |
| 534 | REPLACEMENT_LONG_LONG | 2287218-2289441 | 2224 | 2183177-2185325 | 2149 | - | - | N/A | ancestral: HBSQ |
| 535 | - | 2289442-2299738 | 10297 | 2185326-2195621 | 10296 | - | - | - | - |
| 536 | REPLACEMENT_MEDIUM_SHORT | 2299739-2300080 | 342 | 2195622-2195735 | 114 | - | - | intergenic | - |
| 537 | - | 2300081-2306198 | 6118 | 2195736-2201841 | 6106 | - | - | - | - |
| 538 | INDEL_SHORT | 2306199-2306242 | 44 | - | - | 4 | 48 | intergenic | - |
| 539 | - | 2306243-2318285 | 12043 | 2201838-2213867 | 12030 | - | - | - | - |
| 540 | INDEL_LONG | 2318286-2322586 | 4301 | - | - | 11 | 4312 | in_gene | sequence not affected |
| 541 | - | 2322587-2325182 | 2596 | 2213857-2216452 | 2596 | - | - | - | - |
| 542 | REPLACEMENT_SHORT_SHORT | 2325183-2325244 | 62 | 2216453-2216465 | 13 | - | - | in_gene | sequence not affected |
| 543 | - | 2325245-2325488 | 244 | 2216466-2216709 | 244 | - | - | - | - |
| 544 | REPLACEMENT_LONG_MEDIUM | 2325489-2328808 | 3320 | 2216710-2217542 | 833 | - | - | N/A | - |
| 545 | - | 2328809-2329452 | 644 | 2217543-2218187 | 645 | - | - | - | - |
| 546 | INSERT_REPEAT | 2329453-2329504 | 52 | 2218188-2218192 | 5 | - | - | in_gene | sequence not affected; HqIRS46(core_deletion) |
| 547 | - | 2329505-2330207 | 703 | 2218193-2218895 | 703 | - | - | - | - |
| 548 | REPLACEMENT_LONG_MEDIUM | 2330208-2332309 | 2102 | 2218896-2219136 | 241 | - | - | N/A | - |
| 549 | - | 2332310-2347564 | 15255 | 2219137-2234366 | 15230 | - | - | - | - |
| 550 | INSERT_TRANSPOSON | 2347565-2348849 | 1285 | - | - | 7 | 1292 | intergenic | ISHwa4 |
| 551 | - | 2348850-2349782 | 933 | 2234360-2235290 | 931 | - | - | - | - |
| 552 | REPLACEMENT_LONG_MEDIUM | 2349783-2351951 | 2169 | 2235291-2235449 | 159 | - | - | N/A | - |
| 553 | - | 2351952-2363943 | 11992 | 2235450-2247436 | 11987 | - | - | - | - |
| 554 | INSERT_TRANSPOSON | 2363944-2365228 | 1285 | - | - | 7 | 1292 | intergenic | ISHwa4 |
| 555 | - | 2365229-2367512 | 2284 | 2247430-2249713 | 2284 | - | - | - | - |
| 556 | REPLACEMENT_LONG_LONG | 2367513-2383440 | 15928 | 2249714-2252070 | 2357 | - | - | N/A | - |
| 557 | - | 2383441-2384004 | 564 | 2252071-2252634 | 564 | - | - | - | - |
| 558 | INDEL_SHORT | 2384005-2384014 | 10 | - | - | 10 | 20 | intergenic | - |
| 559 | - | 2384015-2385092 | 1078 | 2252625-2253697 | 1073 | - | - | - | - |
| 560 | INSERT_TRANSPOSON | 2385093-2385093 | 1 | 2253698-2255146 | 1449 | - | - | in_gene | ISHwa18 |
| 561 | - | 2385094-2391221 | 6128 | 2255147-2261268 | 6122 | - | - | - | - |
| 562 | INDEL_POLYREPEAT | 2391222-2391500 | 279 | 2261269-2261421 | 153 | - | - | intergenic | - |
| 563 | - | 2391501-2397080 | 5580 | 2261422-2267006 | 5585 | - | - | - | - |
| 564 | REPLACEMENT_LONG_MEDIUM | 2397081-2398685 | 1605 | 2267007-2267475 | 469 | - | - | N/A | - |
| 565 | - | 2398686-2400500 | 1815 | 2267476-2269287 | 1812 | - | - | - | - |
| 566 | INDEL_LONG | 2400501-2403144 | 2644 | - | - | 1 | 2645 | intergenic | - |
| 567 | - | 2403145-2404941 | 1797 | 2269287-2271082 | 1796 | - | - | - | - |
| 568 | REPLACEMENT_MEDIUM_MEDIUM | 2404942-2405765 | 824 | 2271083-2271307 | 225 | - | - | N/A | - |
| 569 | - | 2405766-2406164 | 399 | 2271308-2271706 | 399 | - | - | - | - |
| 570 | INSERT_TRANSPOSON | 2406165-2407449 | 1285 | - | - | 7 | 1292 | intergenic | ISHwa4 |
| 571 | - | 2407450-2407878 | 429 | 2271700-2272129 | 430 | - | - | - | - |
| 572 | INDEL_POLYREPEAT | 2407879-2408201 | 323 | 2272130-2272451 | 322 | - | - | intergenic | - |
| 573 | - | 2408202-2408251 | 50 | 2272452-2272501 | 50 | - | - | - | - |
| 574 | INDEL_SHORT | - | - | 2272502-2272546 | 45 | 2 | 47 | intergenic | - |
| 575 | - | 2408250-2409035 | 786 | 2272547-2273332 | 786 | - | - | - | - |
| 576 | REPLACEMENT_LONG_LONG | 2409036-2410943 | 1908 | 2273333-2274963 | 1631 | - | - | N/A | - |
| 577 | - | 2410944-2411938 | 995 | 2274964-2275946 | 983 | - | - | - | - |
| 578 | REPLACEMENT_LONG_SHORT | 2411939-2454921 | 42983 | 2275947-2275978 | 32 | - | - | N/A | DV10 |
| 579 | - | 2454922-2455461 | 540 | 2275979-2276510 | 532 | - | - | - | - |
| 580 | REPLACEMENT_SHORT_SHORT | 2455462-2455480 | 19 | 2276511-2276552 | 42 | - | - | in_gene | - |
| 581 | - | 2455481-2455554 | 74 | 2276553-2276626 | 74 | - | - | - | - |
| 582 | REPLACEMENT_LONG_LONG | 2455555-2474175 | 18621 | 2276627-2278399 | 1773 | - | - | N/A | ancestral: C23 |
| 583 | - | 2474176-2474645 | 470 | 2278400-2278887 | 488 | - | - | - | - |
| 584 | REPLACEMENT_LONG_MEDIUM | 2474646-2474999 | 354 | 2278888-2281653 | 2766 | - | - | N/A | - |
| 585 | - | 2475000-2480426 | 5427 | 2281654-2287084 | 5431 | - | - | - | - |
| 586 | REPLACEMENT_SHORT_SHORT | 2480427-2480553 | 127 | 2287085-2287233 | 149 | - | - | intergenic | - |
| 587 | - | 2480554-2491677 | 11124 | 2287234-2298343 | 11110 | - | - | - | - |
| 588 | REPLACEMENT_SHORT_SHORT | 2491678-2491803 | 126 | 2298344-2298465 | 122 | - | - | in_gene | - |
| 589 | - | 2491804-2492272 | 469 | 2298466-2298933 | 468 | - | - | - | - |
| 590 | INDEL_MEDIUM | 2492273-2492974 | 702 | - | - | 3 | 705 | in_gene | sequence not affected |
| 591 | - | 2492975-2509365 | 16391 | 2298931-2315331 | 16401 | - | - | - | - |
| 592 | INDEL_SHORT | 2509366-2509468 | 103 | - | - | 1 | 104 | intergenic | - |
| 593 | - | 2509469-2528884 | 19416 | 2315331-2334724 | 19394 | - | - | - | - |
| 594 | INSERT_MITE | 2528885-2529048 | 164 | - | - | 8 | 172 | intergenic | HqIRS36 |
| 595 | - | 2529049-2531127 | 2079 | 2334717-2336795 | 2079 | - | - | - | - |
| 596 | INDEL_POLYREPEAT | 2531128-2531197 | 70 | 2336796-2336829 | 34 | - | - | in_gene | reading frame conserved |
| 597 | - | 2531198-2546242 | 15045 | 2336830-2351850 | 15021 | - | - | - | - |
| 598 | INSERT_MITE | - | - | 2351851-2352015 | 165 | 9 | 174 | intergenic | HqIRS36 |
| 599 | - | 2546234-2551938 | 5705 | 2352016-2357731 | 5716 | - | - | - | - |
| 600 | REPLACEMENT_SHORT_SHORT | 2551939-2551950 | 12 | 2357732-2357754 | 23 | - | - | in_repeat | - |
| 601 | - | 2551951-2558794 | 6844 | 2357755-2364588 | 6834 | - | - | - | - |
| 602 | REPLACEMENT_LONG_LONG | 2558795-2561002 | 2208 | 2364589-2366865 | 2277 | - | - | N/A | - |
| 603 | - | 2561003-2562435 | 1433 | 2366866-2368297 | 1432 | - | - | - | - |
| 604 | REPLACEMENT_LONG_LONG | 2562436-2564069 | 1634 | 2368298-2369972 | 1675 | - | - | N/A | - |
| 605 | - | 2564070-2572978 | 8909 | 2369973-2378890 | 8918 | - | - | - | - |
| 606 | INDEL_SHORT | - | - | 2378891-2379018 | 128 | - | - | intergenic | - |
| 607 | - | 2572979-2575544 | 2566 | 2379019-2381584 | 2566 | - | - | - | - |
| 608 | INSERT_TRANSPOSON | 2575545-2578205 | 2661 | - | - | 7 | 2668 | intergenic | ISHwa8 |
| 609 | - | 2578206-2584255 | 6050 | 2381578-2387627 | 6050 | - | - | - | - |
| 610 | INDEL_MEDIUM | - | - | 2387628-2388193 | 566 | - | - | in_transposon | - |
| 611 | - | 2584256-2587269 | 3014 | 2388194-2391206 | 3013 | - | - | - | - |
| 612 | INSERT_TRANSPOSON | 2587270-2587277 | 8 | 2391207-2392888 | 1682 | - | - | intergenic | ISHwa26 |
| 613 | - | 2587278-2590161 | 2884 | 2392889-2395764 | 2876 | - | - | - | - |
| 614 | INSERT_MITE | - | - | 2395765-2396018 | 254 | 10 | 264 | intergenic | HqIRS35 |
| 615 | - | 2590152-2592319 | 2168 | 2396019-2398178 | 2160 | - | - | - | - |
| 616 | INSERT_REPEAT | 2592320-2592324 | 5 | 2398179-2398569 | 391 | - | - | intergenic | HqIRS46 |
| 617 | - | 2592325-2607421 | 15097 | 2398570-2413670 | 15101 | - | - | - | - |
| 618 | INDEL_SHORT | - | - | 2413671-2413733 | 63 | 5 | 68 | intergenic | - |
| 619 | - | 2607417-2610366 | 2950 | 2413734-2416682 | 2949 | - | - | - | - |
| 620 | REPLACEMENT_SHORT_SHORT | 2610367-2610416 | 50 | 2416683-2416717 | 35 | - | - | in_transposon | - |
| 621 | - | 2610417-2637537 | 27121 | 2416718-2443843 | 27126 | - | - | - | - |
| 622 | INDEL_POLYREPEAT | 2637538-2637561 | 24 | 2443844-2443879 | 36 | - | - | in_repeat | - |
| 623 | - | 2637562-2640851 | 3290 | 2443880-2447181 | 3302 | - | - | - | - |
| 624 | INDEL_LONG | 2640852-2645132 | 4281 | - | - | 4 | 4285 | intergenic | - |
| 625 | - | 2645133-2648802 | 3670 | 2447178-2450846 | 3669 | - | - | - | - |
| 626 | INSERT_REPEAT | 2648803-2648806 | 4 | 2450847-2451517 | 671 | - | - | intergenic | HqIRS56(targeted) |
| 627 | - | 2648807-2660835 | 12029 | 2451518-2463561 | 12044 | - | - | - | - |
| 628 | INSERT_REPEAT | 2660836-2660915 | 80 | - | - | 1 | 81 | intergenic | HqIRS41 |
| 629 | - | 2660916-2674308 | 13393 | 2463561-2476949 | 13389 | - | - | - | - |
| 630 | REPLACEMENT_SHORT_SHORT | 2674309-2674353 | 45 | 2476950-2476985 | 36 | - | - | intergenic | - |
| 631 | - | 2674354-2683325 | 8972 | 2476986-2485959 | 8974 | - | - | - | - |
| 632 | INSERT_REPEAT | - | - | 2485960-2486069 | 110 | 1 | 111 | intergenic | HqIRS55 |
| 633 | - | 2683325-2687621 | 4297 | 2486070-2490380 | 4311 | - | - | - | - |
| 634 | INDEL_POLYREPEAT | 2687622-2687639 | 18 | 2490381-2490428 | 48 | - | - | intergenic | - |
| 635 | - | 2687640-2690022 | 2383 | 2490429-2492811 | 2383 | - | - | - | - |
| 636 | INSERT_REPEAT | 2690023-2690027 | 5 | 2492812-2492971 | 160 | - | - | intergenic | HqIRS81 |
| 637 | - | 2690028-2697998 | 7971 | 2492972-2500944 | 7973 | - | - | - | - |
| 638 | INSERT_MITE | 2697999-2698251 | 253 | - | - | 10 | 263 | intergenic | HqIRS35 |
| 639 | - | 2698252-2713714 | 15463 | 2500935-2516403 | 15469 | - | - | - | - |
| 640 | INDEL_MEDIUM | 2713715-2713961 | 247 | - | - | 5 | 252 | intergenic | - |
| 641 | - | 2713962-2717681 | 3720 | 2516399-2520109 | 3711 | - | - | - | - |
| 642 | REPLACEMENT_LONG_MEDIUM | 2717682-2730852 | 13171 | 2520110-2520298 | 189 | - | - | N/A | ancestral: HBSQ001 |
| 643 | - | 2730853-2741880 | 11028 | 2520299-2531328 | 11030 | - | - | - | - |
| 644 | INSERT_REPEAT | 2741881-2741932 | 52 | 2531329-2531332 | 4 | - | - | in_gene | ISHwa23(core_deletion) |
| 645 | - | 2741933-2747899 | 5967 | 2531333-2537318 | 5986 | - | - | - | - |
| 646 | INSERT_REPEAT | 2747900-2748431 | 532 | 2537319-2537322 | 4 | - | - | intergenic | HqIRS56 |
| 647 | - | 2748432-2753680 | 5249 | 2537323-2542572 | 5250 | - | - | - | - |
| 648 | INDEL_SHORT | - | - | 2542573-2542582 | 10 | 14 | 24 | in_gene | reading frame conserved |
| 649 | - | 2753667-2776951 | 23285 | 2542583-2565867 | 23285 | - | - | - | - |
| 650 | INSERT_TRANSPOSON | 2776952-2776955 | 4 | 2565868-2567541 | 1674 | - | - | intergenic | ISHwa29 |
| 651 | - | 2776956-2780658 | 3703 | 2567542-2571271 | 3730 | - | - | - | - |
| 652 | INSERT_TRANSPOSON | 2780659-2780663 | 5 | 2571272-2572757 | 1486 | - | - | intergenic | ISHwa19 |
| 653 | - | 2780664-2785096 | 4433 | 2572758-2577191 | 4434 | - | - | - | - |
| 654 | INSERT_MITE | 2785097-2785272 | 176 | - | - | - | - | intergenic | HqIRS36 |
| 655 | - | 2785273-2785347 | 75 | 2577192-2577266 | 75 | - | - | - | - |
| 656 | REPLACEMENT_SWITCHREPEATS | 2785348-2785602 | 255 | 2577267-2577520 | 254 | - | - | intergenic | HqIRS35(fwd/rev) |
| 657 | - | 2785603-2810704 | 25102 | 2577521-2602625 | 25105 | - | - | - | - |
| 658 | INDEL_LONG | - | - | 2602626-2655217 | 52592 | 43 | 52635 | in_tRNA | DV11,GI3; ancestral: C23 |
| 659 | - | 2810662-2852615 | 41954 | 2655218-2697164 | 41947 | - | - | - | - |
| 660 | INSERT_TRANSPOSON | 2852616-2852634 | 19 | 2697165-2699201 | 2037 | - | - | intergenic | ISHwa17 |
| 661 | - | 2852635-2870210 | 17576 | 2699202-2716782 | 17581 | - | - | - | - |
| 662 | DELETE_REPEATCORE | 2870211-2870529 | 319 | - | - | 22 | 341 | in_repeat | HqIRS46(core_deletion) |
| 663 | - | 2870530-2873577 | 3048 | 2716761-2719808 | 3048 | - | - | - | - |
| 664 | INDEL_MEDIUM | - | - | 2719809-2720785 | 977 | - | - | intergenic | - |
| 665 | - | 2873578-2879443 | 5866 | 2720786-2726651 | 5866 | - | - | - | - |
| 666 | INDEL_SHORT | - | - | 2726652-2726691 | 40 | 8 | 48 | in_transposon | - |
| 667 | - | 2879436-2882992 | 3557 | 2726692-2730250 | 3559 | - | - | - | - |
| 668 | INSERT_REPEAT | 2882993-2883463 | 471 | 2730251-2730254 | 4 | - | - | intergenic | HqIRS57 |
| 669 | - | 2883464-2884566 | 1103 | 2730255-2731357 | 1103 | - | - | - | - |
| 670 | INDEL_LONG | - | - | 2731358-2733324 | 1967 | 9 | 1976 | in_transposon | ancestral: HBSQ |
| 671 | - | 2884558-2890643 | 6086 | 2733325-2739414 | 6090 | - | - | - | - |
| 672 | INSERT_TRANSPOSON | 2890644-2892485 | 1842 | 2739415-2739418 | 4 | - | - | in_transposon | ISHwa21(targeted) |
| 673 | - | 2892486-2897291 | 4806 | 2739419-2744224 | 4806 | - | - | - | - |
| 674 | INDEL_SHORT | 2897292-2897342 | 51 | - | - | 1 | 52 | intergenic | - |
| 675 | - | 2897343-2924166 | 26824 | 2744224-2771047 | 26824 | - | - | - | - |
| 676 | INSERT_REPEAT | 2924167-2924463 | 297 | - | - | - | - | intergenic | HqIRS40 |
| 677 | - | 2924464-2940364 | 15901 | 2771048-2786950 | 15903 | - | - | - | - |
| 678 | INSERT_REPEAT | 2940365-2940417 | 53 | 2786951-2786954 | 4 | - | - | intergenic | HqIRS47(core_deletion) |
| 679 | - | 2940418-2948824 | 8407 | 2786955-2795359 | 8405 | - | - | - | - |
| 680 | INSERT_MITE | 2948825-2948942 | 118 | - | - | 8 | 126 | in_repeat | HqIRS39 |
| 681 | - | 2948943-2952487 | 3545 | 2795352-2798882 | 3531 | - | - | - | - |
| 682 | REPLACEMENT_MEDIUM_SHORT | 2952488-2952521 | 34 | 2798883-2799839 | 957 | - | - | in_transposon | DV12,GI4; ancestral: HBSQ |
| 683 | - | 2952522-2963866 | 11345 | 2799840-2811201 | 11362 | - | - | - | - |
| 684 | INSERT_TRANSPOSON | 2963867-2964868 | 1002 | - | - | 8 | 1010 | in_repeat | DV12,GI4; ISHwa6 |
| 685 | - | 2964869-2970812 | 5944 | 2811194-2817137 | 5944 | - | - | - | - |
| 686 | INDEL_MEDIUM | - | - | 2817138-2818448 | 1311 | 2 | 1313 | intergenic | DV12,GI4 |
| 687 | - | 2970811-2979146 | 8336 | 2818449-2826799 | 8351 | - | - | - | - |
| 688 | INSERT_REPEAT | 2979147-2979150 | 4 | 2826800-2826851 | 52 | - | - | intergenic | DV12,GI4; ISHwa23(core_deletion) |
| 689 | - | 2979151-2989257 | 10107 | 2826852-2836959 | 10108 | - | - | - | - |
| 690 | INSERT_REPEAT | 2989258-2989269 | 12 | 2836960-2837359 | 400 | - | - | intergenic | DV12,GI4; HqIRS46 |
| 691 | - | 2989270-2993144 | 3875 | 2837360-2841232 | 3873 | - | - | - | - |
| 692 | REPLACEMENT_LONG_MEDIUM | 2993145-2993573 | 429 | 2841233-2945868 | 104636 | - | - | N/A | DV12,GI4; ancestral: C23 |
| 693 | - | 2993574-2993784 | 211 | 2945869-2946079 | 211 | - | - | - | - |
| 694 | REPLACEMENT_SWITCHREPEATS | 2993785-2993892 | 108 | 2946080-2946189 | 110 | - | - | intergenic | DV12,GI4; HqIRS42(fwd/rev) |
| 695 | - | 2993893-2994777 | 885 | 2946190-2947074 | 885 | - | - | - | - |
| 696 | INSERT_TRANSPOSON | 2994778-2994781 | 4 | 2947075-2948526 | 1452 | - | - | in_gene | DV12,GI4; HqIRS98 |
| 697 | - | 2994782-2996060 | 1279 | 2948527-2949790 | 1264 | - | - | - | - |
| 698 | REPLACEMENT_LONG_LONG | 2996061-3001544 | 5484 | 2949791-2983415 | 33625 | - | - | N/A | DV12,GI4; ancestral: C23 |
| 699 | - | 3001545-3004281 | 2737 | 2983416-2986154 | 2739 | - | - | - | - |
| 700 | INDEL_SHORT | 3004282-3004319 | 38 | - | - | 8 | 46 | intergenic | DV12,GI4 |
| 701 | - | 3004320-3022436 | 18117 | 2986147-3004267 | 18121 | - | - | - | - |
| 702 | INDEL_SHORT | - | - | 3004268-3004297 | 30 | 9 | 39 | in_gene | DV12,GI4; reading frame conserved |
| 703 | - | 3022428-3024116 | 1689 | 3004298-3005986 | 1689 | - | - | - | - |
| 704 | INDEL_LONG | - | - | 3005987-3008134 | 2148 | - | - | intergenic | DV12,GI4 |
| 705 | - | 3024117-3041471 | 17355 | 3008135-3025491 | 17357 | - | - | - | - |
| 706 | INDEL_POLYREPEAT | 3041472-3041485 | 14 | 3025492-3025526 | 35 | - | - | in_repeat | - |
| 707 | - | 3041486-3042026 | 541 | 3025527-3026061 | 535 | - | - | - | - |
| 708 | REPLACEMENT_SWITCHREPEATS | 3042027-3042280 | 254 | 3026062-3026321 | 260 | - | - | in_repeat | HqIRS35(fwd/rev) |
| 709 | - | 3042281-3078434 | 36154 | 3026322-3062472 | 36151 | - | - | - | - |
| 710 | INSERT_MITE | 3078435-3078688 | 254 | - | - | 10 | 264 | in_repeat | HqIRS35 |
| 711 | - | 3078689-3078903 | 215 | 3062463-3062693 | 231 | - | - | - | - |
| 712 | INDEL_MEDIUM | 3078904-3079196 | 293 | - | - | 3 | 296 | in_repeat | - |
| 713 | - | 3079197-3087017 | 7821 | 3062691-3070459 | 7769 | - | - | - | - |
| 714 | INDEL_MEDIUM | - | - | 3070460-3070666 | 207 | 2 | 209 | intergenic | - |
| 715 | - | 3087016-3136187 | 49172 | 3070667-3119826 | 49160 | - | - | - | - |
| 716 | INSERT_REPEAT | 3136188-3136294 | 107 | - | - | 3 | 110 | in_repeat | HqIRS55 |
| 717 | - | 3136295-3142429 | 6135 | 3119824-3125958 | 6135 | - | - | - | - |
| 718 | INSERT_REPEAT | 3142430-3142434 | 5 | 3125959-3126346 | 388 | - | - | in_transposon | HqIRS46 |
| 719 | - | 3142435-3145698 | 3264 | 3126347-3129601 | 3255 | - | - | - | - |
| 720 | INSERT_REPEAT | 3145699-3145703 | 5 | 3129602-3130164 | 563 | - | - | intergenic | HqIRS56 |
| 721 | - | 3145704-3148034 | 2331 | 3130165-3132494 | 2330 | - | - | - | - |
|  |  |  |  |  |  |  |  |  |  |
