# Supplementary material for: Haloquadratum walsbyi : Limited Diversity in a Global Pond
Source: PLoS One. 2011 Jun 20;6(6):e20968. doi: 10.1371/journal.pone.0020968 (PMC3119063; doi:10.1371/journal.pone.0020968)
Supplement: Table S6 — Listing of indels which are bounded by direct repeats. This table is a subset of Table S4, and contains only those indels bounded by direct repeats. The same region numbers are used. All other columns are as described for Table S4. The indels are sorted according to the length in the direct repeat (overlap column). Transposons and MITEs, which cause target duplications, have been excluded from this table. (DOC) [file pone.0020968.s007.doc]

### Table S6. Listing of indels which are bounded by direct repeats

| **Region Number** | **Region Category** | **Strain C23^T^** | | **Strain HBSQ001** | | **Overlap** | **SUM** (len+ovl) | **Location** | **Description** |
| --- | --- | --- | --- | --- | --- | --- | --- | --- | --- |
|  |  | **position** | **length** | **position** | **length** |  |  |  |  |
| 278 | INDEL_MEDIUM | 1331764-1332370 | 607 | - | - | 734 | 1341 | intergenic | DV6,GI2 |
| 290 | INDEL_LONG | - | - | 1370762-1373427 | 2666 | 139 | 2805 | intergenic | DV6,GI2 |
| 658 | INDEL_LONG | - | - | 2602626-2655217 | 52592 | 43 | 52635 | in_tRNA | DV11,GI3; ancestral: C23 |
| 370 | INDEL_LONG | 1615793-1635538 | 19746 | - | - | 22 | 19768 | in_tRNA | DV7; ancestral: HBSQ001 |
| 662 | DELETE_REPEATCORE | 2870211-2870529 | 319 | - | - | 22 | 341 | in_repeat | HqIRS46(core_deletion) |
| 32 | DELETE_REPEATCORE | - | - | 178871-179186 | 316 | 22 | 338 | in_repeat | HqIRS46(core_deletion) |
| 428 | DELETE_REPEATCORE | - | - | 1808130-1809558 | 1429 | 19 | 1448 | in_repeat | ISHwa23 |
| 2 | DELETE_REPEATCORE | - | - | 28013-28332 | 320 | 19 | 339 | in_repeat | HqIRS46(core_deletion) |
| 134 | DELETE_REPEATCORE | - | - | 647855-648174 | 320 | 19 | 339 | in_repeat | HqIRS46(core_deletion) |
| 466 | INDEL_LONG | 1952401-1959157 | 6757 | - | - | 17 | 6774 | in_gene | ancestral: C23 |
| 524 | INDEL_LONG | 2220323-2225758 | 5436 | - | - | 15 | 5451 | in_gene | sequence not affected |
| 648 | INDEL_SHORT | - | - | 2542573-2542582 | 10 | 14 | 24 | in_gene | reading frame conserved |
| 226 | INDEL_MEDIUM | - | - | 1088150-1088462 | 313 | 13 | 326 | intergenic | - |
| 336 | INDEL_SHORT | - | - | 1522022-1522137 | 116 | 13 | 129 | intergenic | ancestral: HBSQ001 |
| 150 | INDEL_SHORT | - | - | 697483-697575 | 93 | 13 | 106 | intergenic | - |
| 282 | INDEL_SHORT | - | - | 1338718-1338760 | 43 | 13 | 56 | in_gene | DV6,GI2 |
| 422 | INDEL_SHORT | - | - | 1785926-1785938 | 13 | 12 | 25 | in_gene | - |
| 100 | INDEL_SHORT | - | - | 456793-456804 | 12 | 12 | 24 | intergenic | - |
| 540 | INDEL_LONG | 2318286-2322586 | 4301 | - | - | 11 | 4312 | in_gene | sequence not affected |
| 350 | INDEL_MEDIUM | - | - | 1563956-1565371 | 1416 | 11 | 1427 | in_gene | - |
| 264 | INDEL_LONG | 1268551-1284680 | 16130 | - | - | 10 | 16141 | in_gene | DV6,GI2; ancestral: C23 |
| 298 | INDEL_LONG | - | - | 1398632-1403402 | 4771 | 10 | 4781 | in_gene | DV6,GI2; ancestral: HBSQ001 |
| 108 | INDEL_SHORT | 484232-484247 | 16 | - | - | 10 | 26 | intergenic | - |
| 436 | INDEL_SHORT | 1882112-1882122 | 11 | - | - | 10 | 21 | intergenic | - |
| 558 | INDEL_SHORT | 2384005-2384014 | 10 | - | - | 10 | 20 | intergenic | - |
| 670 | INDEL_LONG | - | - | 2731358-2733324 | 1967 | 9 | 1976 | in_transposon | ancestral: HBSQ |
| 342 | INDEL_MEDIUM | - | - | 1532496-1533471 | 976 | 9 | 985 | in_transposon | ancestral: HBSQ001 |
| 702 | INDEL_SHORT | - | - | 3004268-3004297 | 30 | 9 | 39 | in_gene | DV12,GI4; reading frame conserved |
| 228 | INDEL_LONG | 1116767-1119250 | 2484 | - | - | 8 | 2492 | in_gene | ancestral: C23 |
| 400 | INDEL_MEDIUM | - | - | 1732994-1733222 | 229 | 8 | 237 | intergenic | - |
| 378 | INDEL_SHORT | 1657073-1657148 | 76 | - | - | 8 | 84 | in_gene | reading frame conserved |
| 666 | INDEL_SHORT | - | - | 2726652-2726691 | 40 | 8 | 48 | in_transposon | - |
| 700 | INDEL_SHORT | 3004282-3004319 | 38 | - | - | 8 | 46 | intergenic | DV12,GI4 |
| 10 | INDEL_SHORT | 73398-73413 | 16 | - | - | 8 | 24 | intergenic | - |
| 148 | INDEL_MEDIUM | 714550-714986 | 437 | - | - | 7 | 444 | intergenic | - |
| 70 | INDEL_SHORT | 358218-358230 | 13 | - | - | 7 | 20 | intergenic | - |
| 388 | INDEL_LONG | 1695969-1730200 | 34232 | - | - | 6 | 34238 | in_gene | DV8; ancestral: C23 |
| 232 | INDEL_LONG | - | - | 1101407-1105429 | 4023 | 6 | 4029 | intergenic | - |
| 280 | INDEL_SHORT | 1339167-1339186 | 20 | - | - | 6 | 26 | in_gene | DV6,GI2 |
| 640 | INDEL_MEDIUM | 2713715-2713961 | 247 | - | - | 5 | 252 | intergenic | - |
| 502 | INDEL_MEDIUM | 2146713-2146876 | 164 | - | - | 5 | 169 | in_transposon | ancestral: C23 |
| 292 | INDEL_MEDIUM | 1383733-1383892 | 160 | - | - | 5 | 165 | intergenic | DV6,GI2; ancestral: HBSQ001 |
| 618 | INDEL_SHORT | - | - | 2413671-2413733 | 63 | 5 | 68 | intergenic | - |
| 358 | INDEL_SHORT | - | - | 1576678-1576702 | 25 | 5 | 30 | intergenic | - |
| 624 | INDEL_LONG | 2640852-2645132 | 4281 | - | - | 4 | 4285 | intergenic | - |
| 266 | INDEL_LONG | 1286415-1288891 | 2477 | - | - | 4 | 2481 | in_gene | DV6,GI2; ancestral: C23 |
| 392 | INDEL_MEDIUM | - | - | 1703661-1704004 | 344 | 4 | 348 | intergenic | - |
| 538 | INDEL_SHORT | 2306199-2306242 | 44 | - | - | 4 | 48 | intergenic | - |
| 198 | INDEL_SHORT | 1008269-1008290 | 22 | - | - | 4 | 26 | intergenic | - |
| 518 | INDEL_SHORT | - | - | 2121269-2121284 | 16 | 4 | 20 | intergenic | - |
| 106 | INDEL_LONG | 472969-477786 | 4818 | - | - | 3 | 4821 | intergenic | - |
| 590 | INDEL_MEDIUM | 2492273-2492974 | 702 | - | - | 3 | 705 | in_gene | sequence not affected |
| 712 | INDEL_MEDIUM | 3078904-3079196 | 293 | - | - | 3 | 296 | in_repeat | - |
| 498 | INDEL_MEDIUM | - | - | 2050858-2051052 | 195 | 3 | 198 | in_transposon | ancestral: HBSQ |
| 6 | INDEL_SHORT | - | - | 37577-37637 | 61 | 3 | 64 | intergenic | - |
| 158 | INDEL_SHORT | 736578-736598 | 21 | - | - | 3 | 24 | intergenic | - |
| 686 | INDEL_MEDIUM | - | - | 2817138-2818448 | 1311 | 2 | 1313 | intergenic | DV12,GI4 |
| 520 | INDEL_MEDIUM | 2214385-2214671 | 287 | - | - | 2 | 289 | intergenic | - |
| 490 | INDEL_MEDIUM | - | - | 2031821-2032056 | 236 | 2 | 238 | intergenic | - |
| 714 | INDEL_MEDIUM | - | - | 3070460-3070666 | 207 | 2 | 209 | intergenic | - |
| 574 | INDEL_SHORT | - | - | 2272502-2272546 | 45 | 2 | 47 | intergenic | - |
| 220 | INDEL_LONG | 1098764-1101694 | 2931 | - | - | 1 | 2932 | intergenic | - |
| 566 | INDEL_LONG | 2400501-2403144 | 2644 | - | - | 1 | 2645 | intergenic | - |
| 12 | INDEL_MEDIUM | - | - | 76424-77200 | 777 | 1 | 778 | intergenic | - |
| 424 | INDEL_MEDIUM | - | - | 1787635-1788393 | 759 | 1 | 760 | in_transposon | - |
| 66 | INDEL_MEDIUM | - | - | 372584-372902 | 319 | 1 | 320 | intergenic | - |
| 406 | INDEL_MEDIUM | - | - | 1755062-1755324 | 263 | 1 | 264 | in_repeat | - |
| 42 | INDEL_SHORT | 237961-238096 | 136 | - | - | 1 | 137 | intergenic | - |
| 592 | INDEL_SHORT | 2509366-2509468 | 103 | - | - | 1 | 104 | intergenic | - |
| 674 | INDEL_SHORT | 2897292-2897342 | 51 | - | - | 1 | 52 | intergenic | - |
| 416 | INDEL_SHORT | 1844131-1844176 | 46 | - | - | 1 | 47 | intergenic | - |
| 236 | INDEL_SHORT | - | - | 1131017-1131052 | 36 | 1 | 37 | intergenic | - |
| 160 | INDEL_SHORT | 745876-745904 | 29 | - | - | 1 | 30 | in_gene | - |
|  |  |  |  |  |  |  |  |  |  |
