# Supplementary material for: Haloquadratum walsbyi : Limited Diversity in a Global Pond
Source: PLoS One. 2011 Jun 20;6(6):e20968. doi: 10.1371/journal.pone.0020968 (PMC3119063; doi:10.1371/journal.pone.0020968)
Supplement: Table S7 — Sequence matches to CRISPR spacers in strains HBSQ001 and C23T. (DOC) [file pone.0020968.s008.doc]

### Table S7. Sequence matches to CRISPR spacers in strains HBSQ001 and C23^T^

| **Strain/spacer** | **Position (nt)** | **Significant matches/strain** | **Source/Location** | **Comment** |
| --- | --- | --- | --- | --- |
| HBSQ001/spacer 2 | 1383407-1383440 | Cas4 gene (Hqrw_2363) of C23^T^ (31/32 nt identical to nt 1401796 - 1401765) | located between crispr-2 and crispr-3 |  |
| HBSQ001/spacer 3 | 1383471-1383487 + 1383645-1383662 | Intergenic region of C23^T^ (31/33 nt identical to nt 1393686 - 1393718) | located between crispr-2 and crispr-3 | Reconstructed (after removal of an inserted HqIRS37 MITE) as: GATAGTCATGGATAAC  ATAAGGCAATGCAAATAGA |
| HBSQ001/spacer 4 | 1383693- 1383728 | Cas6 gene (Hqrw_2358) (32/36 nt identical to nt 1394610 - 1394645) | located between crispr-2 and crispr-3 |  |
| C23^T^/crispr1/spacer2 | 403576-403610 | GU735132 (contig036, nt 58-86,) (29/29 nt, 11/11 aa) | metavirome from solar salterns in Santa Pola, Spain |  |
| C23^T^/crispr1/spacer19 | 404825-404860 | ORF ADE29246 of GU735260.1 (contig165, nt 503-548), probable transcriptional autoregulator (uncultured virus) 36/36 nt identity, 12/12 aa identity. | metavirome from solar salterns in Santa Pola, Spain | **Exact match** |
| C23^T^/crispr1/spacer21 | 404968-405004 | GU735374.1 (virus EHP-C8R) and GU735106.1 (nt 2471-2507, Uncultured virus contig009) 31/35 nt identity and 12/12 aa identity. | metavirome from solar salterns in Santa Pola, Spain |  |
| C23^T^/crispr1/spacer25 | 405260-405295 | GU735124.1 (nt 371-406), uncultured virus contig026, genomic sequence, 36/36 nt identity and 12/12 aa identity. | metavirome from solar salterns in Santa Pola, Spain | **Exact match**. Targets a probable phage/plasmid primase, P4 family protein coded by GU735124. |
| C23^T^/crispr1/spacer29 | 405551-405585 | Related to ORF ADE29296 of GU735358.1 (nt 2315-2343, contig003). Uncultured virus, 27/29 nt identity, 11/11 aa identity. | metavirome from solar salterns in Santa Pola, Spain. |  |
| C23^T^/crispr1/spacer34 | 405916- 405950 | ORF ADE29297 of GU735358.1 (nt 2659 - 2693, contig003), hypothetical membrane protein (uncultured virus), 30/31 nt identity, 10/11 aa identity | metavirome from solar salterns in Santa Pola, Spain. | Metavirome Contig003 shows similarity to halovirus His1_gp16. |
| C23^T^/crispr2/spacer13 | 1391896-1391930 | ORF ADE29126.1 of GU735106.1 (nt 1708-1736, contig009), hypothetical translocation domain protein, 28/29 nt identity, 10/11 aa identity | metavirome from solar salterns in Santa Pola, Spain |  |
| C23^T^/crispr2/spacer17 | 1392157- 1392193 | ORF Hqrw_6004 of plasmid PL6A. 29/36 nt identical, 11/12 aa identical |  |  |
| C23^T^/crispr3/spacer1 | 1403351-1403386 | GU735112.1, nt 3537-3572, uncultured virus contig015, genomic sequence, 35/35 nt identity, 12/12 aa identity. | metavirome from solar salterns in Santa Pola, Spain | **Exact match**. The metavirome target is closely related to *Nmn. pharaonis* plasmid PL23 ORF NP7036A, predicted to be a secreted protein (SignalP) with C-terminal transmembrane domains. |
| C23^T^/crispr3/spacer5 | 1403613-1403646 | Contig015 (GU735112.1, nt 4441-4470), contig002 (GU735101.1, nt 2950-2979) of uncultured virus sequences. 30/30 nt identity, and 10/11 aa indentity. | metavirome from solar salterns in Santa Pola, Spain |  |
| C23^T^/crispr3/spacer6 | 1403677-1403712 | ORF ADE29107 (GU735100.1 nt 1440-1465), contig005. Conserved hypothetical protein (uncultured virus), 10/11 aa identity | metavirome from solar salterns in Santa Pola, Spain | ORF target related to halovirus BJ1 ORF BJ1_gp43, putative function: putative small subunit of the DNA polymerase. |
| C23^T^/crispr3/spacer14 | 1404207-1404243 | GU735112.1 (nt 404-440 of contig015), uncultured virus sequence 37/37 nt and 12/12 aa identical. | metavirome from solar salterns in Santa Pola, Spain | **Exact match**. |
| C23^T^/crispr3/spacer15 | 1404274- 1404309 | ORF ADE29293 of GU735358.1 (nt 417-452, contig003), hypothetical membrane protein (uncultured virus), 33/36 nt identity, 12/12 aa identity. | metavirome from solar salterns in Santa Pola, Spain | Targets different ORF on same contig as C23^T^/crispr3/spacer34. Contig003 shows similarity to halovirus His1. |
| C23^T^/crispr3/spacer20 | 1404608- 1404642 | Strain HBSQ001, ORF HQ2151A (CAJ52277.1), nt 1385100- 1385134 of AM180088, 35/35 nt identity, 11/11 aa identity. | Strain isolated from the Santa Pola saltern, Spain. | **Exact match**. HQ2151A is predicted to be a restriction endonuclease, COG3440. |
