# Supplementary material for: Haloquadratum walsbyi : Limited Diversity in a Global Pond
Source: PLoS One. 2011 Jun 20;6(6):e20968. doi: 10.1371/journal.pone.0020968 (PMC3119063; doi:10.1371/journal.pone.0020968)
Supplement: Table S8 — Listing of all Haloquadratum MGEs. MGE codes start either with ISHwa or with HqIRS. MGEs with ISHwa codes are present in (or submitted to) ISFinder. A prerequisite for an ISFinder submission is that the MGE is complete, its boundaries are defined, and the MGE codes for a complete transposase. Element groups are a clustering of MGEs based on sequence similarity and common characteristics. The categories are: TP-A (IS605-type transposon), TP-B (common transposon), MITE (miniature inverted-repeat transposable elements), PATE (palindrome-associated transposable element), or SMR-A, SMR-B (short mobile repeat of types A, B). Length, indicates the length range of complete copies of the element. A hyphen indicates that none of the copies is complete. The ITR and TD fields contain data only if at least one of the elements has complete boundaries on both sides. ITR provides the inverted terminal repeat of a typical member in ISFinder style where the second number corresponds to the ITR length and the first number to the number of matching bases within the ITR. The term “none” indicates that the MGE does not cause target duplications. TD provides the length range of target duplications. The term “none” indicates that the MGE does not cause target duplications. The #(C23T) and #(HBSQ001) columns provides the total number of MGEs, followed in parenthesis by the number of elements with complete sequences and the number of elements with complete transposases (devoid of deletions, frame-shifts or in-frame stop codons). The SSEI column provides the number of element copies that occur as SSEIs (strain-specific element insertions). Some of these data are replaced by hyphens if the element is not present in the strain (total number is zero. The remarks column specifies the relationship between MITEs and their parent transposons. (DOC) [file pone.0020968.s009.doc]

### Table S8. Listing of all *Haloquadratum* MGEs

| **Code** | **Element Group** | **Category** | **Length** | **ITR** | **TD** | **#(C23)** | **#(HBSQ001)** | **SSEIs**  **(C23/HBSQ001)** | **remarks** |
| --- | --- | --- | --- | --- | --- | --- | --- | --- | --- |
| ISHwa1 | ISH9-type | TP-B | 934-937 | 14/16 | 8 | 1 (1,1) | 2 (2, 1) | 0/1 | - |
| ISHwa3 | ISH9-type | TP-B | 940 | 21/25 | 8 | 1 (1,1) | 1 (0, 0) | 0/0 | - |
| HqIRS1 | ISH9-type | TP-B | 934 | 14/15 | - | 0 (-,-) | 1 (1, 0) | -/0 | - |
| HqIRS2 | ISH9-type | TP-B | 885 | 13/20 | - | 1 (1,0) | 1 (1, 0) | 0/0 | - |
| HqIRS3 | ISH9-type | TP-B | 899 | 15/17 | - | 0 (-,-) | 1 (1, 0) | -/0 | - |
| HqIRS4 | ISH9-type | TP-B | - | - | - | 1 (0,0) | 0 (-,-) | 0/- | - |
| HqIRS5 | ISH9-type | TP-B | - | - | - | 1 (0,0) | 1 (0, 0) | 0/0 | - |
| ISHwa2 | Tn5-type | TP-B | 1661-1668 | 19/20 | 10-11 | 5 (5,5) | 1 (1, 1) | 3/0 | MITE:HqIRS35 |
| HqIRS6 | Tn5-type | TP-B | - | - | - | 1 (0,0) | 1 (0, 0) | 0/0 | - |
| ISHwa4 | ISHwa4-type | TP-B | 1285 | 17/18 | 7-8 | 10 (10,10) | 0 (-,-) | 8/- | - |
| HqIRS7 | ISHwa4-type | TP-B | - | - | - | 1 (0,0) | 3 (0, 0) | 0/0 | - |
| HqIRS8 | ISHwa4-type | TP-B | 1265 | 13/18 | - | 0 (-,-) | 1 (1, 0) | -/0 | - |
| HqIRS9 | ISHwa4-type | TP-B | - | - | - | 0 (-,-) | 1 (0, 0) | -/0 | - |
| ISHwa5 | ISH5-type | TP-B | 2256 | 18/18 | 11 | 1 (1,1) | 0 (-,-) | 1/- | - |
| HqIRS10 | ISH5-type | TP-B | 1405 | 18/20 | - | 0 (-,-) | 1 (1, 0) | -/0 | - |
| ISHwa6 | ISH4-type | TP-B | 1002 | 22/25 | 8-9 | 5 (3,3) | 2 (0, 0) | 1/0 | MITE:HqIRS36 |
| HqIRS11 | ISH4-type | TP-B | 945 | 19/25 | 8 | 2 (2,0) | 0 (-,-) | 2/- | - |
| HqIRS12 | ISH4-type | TP-B | - | - | - | 3 (0,0) | 2 (0, 0) | 0/0 | MITE:HqIRS39 |
| ISHwa7 | IS630-type | TP-B | 1146 | 16/21 | - | 1 (1,1) | 0 (-,-) | 0/- | - |
| HqIRS13 | IS630-type | TP-B | - | - | - | 0 (-,-) | 1 (0, 0) | -/0 | - |
| HqIRS14 | IS630-type | TP-B | 1163 | 18/30 | - | 2 (1,0) | 4 (1, 0) | 0/0 | - |
| HqIRS15 | IS630-type | TP-B | - | - | - | 0 (-,-) | 1 (0, 0) | -/0 | - |
| ISHwa8 | ISH8-type | TP-B | 1509-1516 | 20/23 | 7-9 | 6 (6,2) | 2 (2, 2) | 4/0 | MITE:HqIRS32 |
| ISHwa9 | ISH8-type | TP-B | 1511-1518 | 21/23 | 8 | 2 (2,2) | 0 (-,-) | 2/- | - |
| HqIRS16 | ISH8-type | TP-B | 1459 | 15/20 | - | 0 (-,-) | 1 (1, 0) | -/0 | - |
| HqIRS17 | ISH8-type | TP-B | - | - | - | 0 (-,-) | 1 (0, 0) | -/0 | - |
| HqIRS18 | ISH8-type | TP-B | - | - | - | 0 (-,-) | 1 (0, 0) | -/0 | - |
| HqIRS19 | ISH8-type | TP-B | - | - | - | 1 (0,0) | 0 (-,-) | 0/- | - |
| HqIRS20 | ISH8-type | TP-B | - | - | - | 1 (0,0) | 0 (-,-) | 0/- | - |
| HqIRS21 | ISH8-type | TP-B | - | 16/18 | 10 | 1 (0,0) | 0 (-,-) | 0/- | - |
| HqIRS22 | ISH8-type | TP-B | - | - | - | 0 (-,-) | 1 (0, 0) | -/0 | - |
| ISHwa10 | ISH10-type | TP-B | 1597-1598 | 14/17 | 8 | 2 (2,2) | 0 (-,-) | 1/- | - |
| ISHwa11 | ISH10-type | TP-B | 1579 | 16/20 | 8 | 2 (1,1) | 0 (-,-) | 0/- | - |
| HqIRS23 | ISH10-type | TP-B | - | - | - | 1 (0,0) | 0 (-,-) | 0/- | - |
| ISHwa12 | ISH3-type | TP-B | 1500-1509 | 17/17 | 5 | 6 (5,5) | 0 (-,-) | 4/- | - |
| ISHwa13 | ISH3-type | TP-B | 1459-1465 | 14/14 | 5 | 3 (3,2) | 1 (1, 0) | 2/0 | - |
| HqIRS24 | ISH3-type | TP-B | - | - | - | 1 (0,0) | 1 (0, 0) | 0/0 | - |
| HqIRS25 | ISH3-type | TP-B | 1313 | 10/12 | 5 | 1 (0,0) | 1 (1, 0) | 0/0 | - |
| HqIRS26 | ISH3-type | TP-B | - | - | - | 1 (0,0) | 0 (-,-) | 0/- | - |
| ISHwa14 | ISH11-type | TP-B | 1070 | 21/25 | 8 | 1 (1,1) | 0 (-,-) | 0/- | - |
| HqIRS27 | ISH11-type | TP-B | 1067 | 13/23 | - | 0 (-,-) | 1 (1, 0) | -/0 | - |
| ISHwa20 | ISH11-type | TP-B | 1063-1078 | 22/28 | 6-10 | 4 (3,0) | 1 (0, 0) | 0/0 | MITE:HqIRS37,HqIRS38 |
| ISHwa15 | ISH7-type | TP-B | 1845 | 13/14 | 8 | 1 (1,0) | 1 (0, 0) | 0/0 | - |
| HqIRS29 | ISH7-type | TP-B | - | - | - | 0 (-,-) | 1 (0, 0) | -/0 | - |
| HqIRS30 | ISH7-type | TP-B | - | - | - | 0 (-,-) | 1 (0, 0) | -/0 | - |
| ISHwa16 | ISSod10-type | TP-B | 1070 | 17/27 | - | 1 (1,1) | 1 (1, 1) | 0/0 | - |
| HqIRS31 | IS240-type | TP-B | - | - | - | 2 (0,0) | 1 (0, 0) | 0/0 | - |
| HqIRS32 | ISH2-type | MITE | 416 | 19/23 | 8 | 2 (2,2) | 0 (-,-) | 1/- | TP:ISHwa8 |
| HqIRS33 | ISH2-type | MITE | - | - | - | 1 (0,1) | 0 (-,-) | 0/- | - |
| HqIRS34 | ISH2-type | MITE | - | - | - | 0 (-,-) | 1 (0, 1) | -/0 | - |
| HqIRS35 | HqIRS35-type | MITE | 241-266 | 16/19 | 10 | 34 (30,0) | 36 (34, 0) | 5/5 | TP:ISHwa2, Duanwu in [69] |
| HqIRS36 | HqIRS36-type | MITE | 164-166 | 22/26 | 8-9 | 6 (6,0) | 8 (8, 0) | 4/5 | TP:ISHwa6 , Qixi in [69] |
| HqIRS37 | HqIRS37-type | MITE | 144-149 | 19/24 | 6-8 | 3 (3,0) | 2 (2, 0) | 1/0 | TP:ISHwa20 |
| HqIRS38 | HqIRS37-type | MITE | 146 | 18/24 | - | 1 (1,0) | 1 (1, 0) | 0/0 | TP:ISHwa20 |
| HqIRS39 | HqIRS39-type | MITE | 118-119 | 19/24 | 8-9 | 8 (8,0) | 7 (7, 0) | 3/2 | TP:HqIRS12, Chongyang in [69] |
| HqIRS40 | HqIRS40-type | SMR-A | 296-297 | 18/29 | - | 9 (9,0) | 0 (-,-) | 2/- | - |
| HqIRS41 | HqIRS41-type | SMR-A | 79-80 | 17/21 | - | 6 (6,0) | 0 (-,-) | 3/- | - |
| HqIRS42 | HqIRS42-type | SMR-B | 103-112 | 15/25 | - | 29 (28,0) | 41 (40, 0) | 1/4 | - |
| HqIRS43 | HqIRS43-type | SMR-B | 107-109 | 16/23 | - | 26 (25,0) | 36 (36, 0) | 3/7 | - |
| HqIRS44 | HqIRS44-type | PATE | 405-406 | none | - | 4 (4,0) | 3 (3, 0) | 2/1 | Homogeneous PATE |
| HqIRS45 | HqIRS44-type | PATE | 403-406 | none | - | 6 (1,0) | 6 (2, 0) | 0/0 | - |
| HqIRS46 | HqIRS46-type | PATE | 386-393 | none | - | 16 (9,0) | 22 (17, 0) | 2/6 | Homogeneous PATE |
| HqIRS47 | HqIRS46-type | PATE | 392-396 | none | - | 9 (2,0) | 6 (1, 0) | 2/0 | - |
| HqIRS48 | HqIRS46-type | PATE | 493 | none | - | 0 (-,-) | 1 (1, 0) | -/0 | - |
| HqIRS49 | HqIRS46-type | PATE | 385 | none | - | 1 (1,0) | 1 (1, 0) | 0/0 | - |
| HqIRS50 | HqIRS46-type | PATE | 455 | none | - | 1 (0,0) | 1 (1, 0) | 0/0 | - |
| HqIRS51 | HqIRS46-type | PATE | 376 | none | - | 1 (1,0) | 0 (-,-) | 0/- | - |
| HqIRS52 | HqIRS46-type | PATE | 389-416 | none | - | 2 (1,0) | 2 (2, 0) | 0/0 | - |
| HqIRS53 | HqIRS46-type | PATE | - | - | - | 4 (0,0) | 5 (0, 0) | 0/0 | - |
| HqIRS54 | HqIRS54-type | PATE | 370-381 | none | - | 3 (3,0) | 6 (6, 0) | 0/3 | Homogeneous PATE |
| HqIRS55 | HqIRS55-type | SMR-A | 109-122 | 23/31 | - | 54 (52,0) | 51 (49, 0) | 10/5 | - |
| HqIRS56 | HqIRS56-type | PATE | 532-574 | none | - | 19 (12,0) | 23 (20, 0) | 7/7 | Homogeneous PATE |
| HqIRS57 | HqIRS56-type | PATE | 466-688 | none | - | 12 (12,0) | 14 (14, 0) | 1/2 | - |
| HqIRS58 | HqIRS56-type | PATE | 497-522 | none | - | 8 (7,0) | 8 (7, 0) | 0/0 | - |
| HqIRS59 | HqIRS56-type | PATE | 507-535 | none | - | 5 (3,0) | 5 (3, 0) | 0/0 | - |
| HqIRS60 | HqIRS56-type | PATE | 530-599 | none | - | 20 (13,0) | 19 (14, 0) | 0/0 | - |
| HqIRS61 | HqIRS56-type | PATE | 530-532 | none | - | 2 (2,0) | 2 (2, 0) | 0/0 | - |
| HqIRS62 | HqIRS56-type | PATE | 536-542 | none | - | 1 (1,0) | 1 (1, 0) | 0/0 | - |
| HqIRS63 | HqIRS56-type | PATE | 590-591 | none | - | 5 (1,0) | 5 (1, 0) | 0/0 | - |
| HqIRS64 | HqIRS56-type | PATE | 558 | none | - | 1 (1,0) | 1 (1, 0) | 0/0 | - |
| HqIRS65 | HqIRS56-type | PATE | 517 | none | - | 1 (1,0) | 1 (1, 0) | 0/0 | - |
| HqIRS66 | HqIRS56-type | PATE | 543 | none | - | 1 (1,0) | 1 (1, 0) | 0/0 | - |
| HqIRS67 | HqIRS56-type | PATE | 572 | none | - | 1 (1,0) | 0 (-,-) | 0/- | - |
| HqIRS68 | HqIRS56-type | PATE | - | none | - | 1 (0,0) | 1 (0, 0) | 0/0 | - |
| HqIRS69 | HqIRS56-type | PATE | - | none | - | 1 (0,0) | 1 (0, 0) | 0/0 | - |
| HqIRS70 | HqIRS56-type | PATE | - | none | - | 4 (0,0) | 4 (0, 0) | 0/0 | - |
| HqIRS71 | HqIRS71-type | PATE | 543-590 | none | - | 9 (5,0) | 15 (11, 0) | 0/5 | Homogeneous PATE |
| HqIRS72 | HqIRS71-type | PATE | 340-341 | none | - | 1 (1,0) | 1 (1, 0) | 0/0 | - |
| HqIRS73 | HqIRS71-type | PATE | 383-396 | none | - | 4 (4,0) | 3 (3, 0) | 0/0 | - |
| HqIRS74 | HqIRS71-type | PATE | 380-386 | none | - | 3 (3,0) | 3 (3, 0) | 0/0 | - |
| HqIRS75 | HqIRS71-type | PATE | 376-390 | none | - | 3 (3,0) | 3 (3, 0) | 0/0 | - |
| HqIRS76 | HqIRS71-type | PATE | 368-378 | none | - | 1 (1,0) | 1 (1, 0) | 0/0 | - |
| HqIRS77 | HqIRS71-type | PATE | 376 | none | - | 1 (1,0) | 1 (1, 0) | 0/0 | - |
| HqIRS78 | HqIRS71-type | PATE | 389 | none | - | 1 (1,0) | 1 (1, 0) | 0/0 | - |
| HqIRS79 | HqIRS71-type | PATE | - | - | - | 1 (0,0) | 1 (0, 0) | 0/0 | - |
| HqIRS80 | HqIRS71-type | PATE | - | - | - | 1 (0,0) | 1 (0, 0) | 0/0 | - |
| HqIRS81 | HqIRS81-type | PATE | 51-59 | none | - | 16 (12,0) | 16 (12, 0) | 0/1 | - |
| HqIRS82 | HqIRS81-type | PATE | 55-60 | none | - | 4 (4,0) | 6 (6, 0) | 0/1 | - |
| HqIRS83 | HqIRS81-type | PATE | - | - | - | 3 (0,0) | 3 (0, 0) | 0/0 | - |
| ISHwa17 | ISNph7-type | TP-A | 2035-2037 | none | - | 4 (0,0) | 8 (3, 0) | 2/4 | - |
| ISHwa18 | ISNph15-type | TP-A | 1453 | none | - | 0 (-,-) | 1 (1, 1) | -/1 | - |
| ISHwa19 | ISNph15-type | TP-A | 1486 | none | - | 0 (-,-) | 1 (1, 1) | -/1 | - |
| HqIRS84 | ISNph15-type | TP-A | 1590 | none | - | 1 (0,0) | 2 (1, 0) | 0/0 | - |
| HqIRS107 | ISNph15-type | TP-A | - | none | - | 1 (0,0) | 1 (0, 0) | 0/0 | - |
| ISHwa21 | ISHwa21-type | TP-A | 1576-1604 | none | - | 2 (2,2) | 2 (2, 2) | 2/1 | - |
| ISHwa22 | ISHwa21-type | TP-A | 1545 | none | - | 1 (0,0) | 1 (1, 1) | 0/0 | - |
| ISHwa26 | ISHwa21-type | TP-A | 1574-1593 | none | - | 1 (1,1) | 1 (1, 1) | 1/1 | - |
| HqIRS85 | ISHwa21-type | TP-A | - | - | - | 1 (0,0) | 1 (0, 0) | 0/0 | - |
| HqIRS86 | ISHwa21-type | TP-A | - | - | - | 8 (0,0) | 8 (0, 0) | 0/0 | - |
| HqIRS108 | ISHwa21-type | TP-A | - | none | - | 1 (0,0) | 1 (0, 0) | 0/0 | - |
| ISHwa23 | ISHwa23-type | TP-A | 1500 | none | - | 6 (1,1) | 10 (2, 2) | 2/7 | - |
| ISHwa24 | ISHwa23-type | TP-A | 1504 | none | - | 1 (1,1) | 1 (1, 1) | 0/0 | - |
| HqIRS87 | ISHwa23-type | TP-A | - | - | - | 1 (0,0) | 1 (0, 0) | 0/0 | - |
| HqIRS106 | ISHwa23-type | TP-A | - | none | - | 3 (0,0) | 2 (0, 0) | 0/0 | - |
| ISHwa25 | ISNph10-type | TP-A | 1355 | none | - | 1 (0,0) | 2 (2, 1) | 0/0 | - |
| HqIRS88 | ISNph10-type | TP-A | - | none | - | 3 (0,0) | 5 (0, 0) | 0/0 | - |
| HqIRS89 | ISNph6-type | TP-A | - | none | - | 8 (0,0) | 8 (0, 0) | 0/0 | - |
| HqIRS90 | ISNph6-type | TP-A | - | - | - | 2 (0,0) | 2 (0, 0) | 0/0 | - |
| HqIRS91 | HqIRS91-type | TP-A | 1591-1626 | none | - | 1 (1,0) | 1 (1, 0) | 0/0 | - |
| HqIRS92 | ISNph20-type | TP-A | - | - | - | 1 (0,0) | 1 (0, 0) | 0/0 | - |
| HqIRS93 | HqIRS93-type | TP-A | 1479 | none | - | 1 (0,0) | 2 (1, 0) | 0/0 | - |
| ISHwa27 | ISHwa27-type | TP-A | 1492 | none | - | 3 (0,0) | 3 (1, 1) | 0/0 | - |
| HqIRS94 | ISNph14-type | TP-A | 1523 | none | - | 2 (1,0) | 2 (1, 0) | 0/0 | - |
| ISHwa28 | ISNph20-type | TP-A | 1486-1536 | none | - | 2 (1,0) | 3 (3, 1) | 0/0 | - |
| HqIRS95 | ISNph20-type | TP-A | - | - | - | 1 (0,0) | 1 (0, 0) | 0/0 | - |
| HqIRS96 | HqIRS96-type | TP-A | - | - | - | 1 (0,0) | 1 (0, 1) | 0/0 | - |
| ISHwa29 | ISHwa29-type | TP-A | 1674 | none | - | 3 (0,0) | 4 (1, 1) | 0/1 | - |
| HqIRS97 | ISHwa29-type | TP-A | - | none | - | 1 (0,0) | 1 (0, 0) | 0/0 | - |
| HqIRS98 | HqIRS98-type | TP-A | 1452 | none | - | 0 (-,-) | 1 (1, 0) | -/1 | - |
| HqIRS99 | HqIRS99-type | TP-A | - | - | - | 0 (-,-) | 1 (0, 1) | -/0 | - |
| HqIRS100 | HqIRS100-type | TP-A | - | none | - | 2 (0,0) | 2 (0, 0) | 0/0 | - |
| HqIRS101 | HqIRS101-type | TP-A | - | - | - | 1 (0,0) | 2 (0, 0) | 0/0 | - |
| HqIRS102 | HqIRS102-type | TP-A | - | - | - | 1 (0,0) | 1 (0, 0) | 0/0 | - |
| HqIRS103 | ISNph17-type | TP-A | - | - | - | 1 (0,0) | 1 (0, 0) | 0/0 | - |
| HqIRS104 | HqIRS104-type | TP-A | - | - | - | 1 (0,0) | 1 (0, 0) | 0/0 | - |
| HqIRS105 | HqIRS105-type | TP-A | - | - | - | 1 (0,0) | 1 (0, 0) | 0/0 | - |
| HqIRS109 | ISNph19-type | TP-A | - | none | - | 1 (0,0) | 1 (0, 0) | 0/0 | - |
| HqIRS110 | HqIRS110-type | TP-A | - | none | - | 1 (0,0) | 1 (0, 0) | 0/0 | - |
| HqIRS111 | HqIRS111-type | TP-A | - | none | - | 1 (0,0) | 1 (0, 0) | 0/0 | - |
| HqIRS112 | HqIRS112-type | TP-A | - | none | - | 1 (0,0) | 1 (0, 0) | 0/0 | - |
| HqIRS113 | ISNph21-type | TP-A | - | - | - | 1 (0,0) | 1 (0, 0) | 0/0 | - |
| HqIRS114 | HqIRS114-type | TP-A | - | - | - | 23 (0,0) | 23 (0, 0) | 0/0 | - |
| HqIRS115 | HqIRS115-type | TP-A | - | none | - | 3 (0,0) | 3 (0, 0) | 0/0 | - |
|  |  |  |  |  |  |  |  |  |  |
