# Supplementary material for: Haloquadratum walsbyi : Limited Diversity in a Global Pond
Source: PLoS One. 2011 Jun 20;6(6):e20968. doi: 10.1371/journal.pone.0020968 (PMC3119063; doi:10.1371/journal.pone.0020968)
Supplement: Table S9 — Near terminal palindromes in homogeneous PATEs and relation to IS605 type transposons. The terminal sequences of homogenous PATEs are compared in several ways. In section A, terminal sequences are compared within each element. The 5′ and 3′ “palindromic end of element” highlights palindromes by comparison of the terminal sequence to its reverse-complement. “Comparison of 5′ and 3′ palindromic ends” highlights similarities between the terminal sequences. For HqIRS46, the 52 bp terminal_only version of the element, generated by repeat-mediated deletion within a perfect 22 bp repeat, is shown (core del). In all cases, internal sequence ends are indicated by three dots. For the 3′ end, both directions show the terminal sequence fragment. It should be noted that the terminal sequences of distinct PATEs show similarities at the very 3′ end. For the 5′ end, the targeting sequence of 4–5 bp is included. The distance between the element start and the near-terminal palindrome is longer at the 5′ end compared to the 3′ end and this extended sequence is not displayed for the reverse strand. In section B, the terminal sequence of the PATE is compared to that of other elements. There is a remarkably high level of sequence similarity between homogenous PATEs from Haloquadratum and IS605-type transposases from Natronomonas pharaonis. (DOC) [file pone.0020968.s010.doc]

### Table S9. Near terminal palindromes in homogeneous PATEs and relation to IS605 type transposons

| **Section A: Near Terminal Palindromes** | | | | |
| --- | --- | --- | --- | --- |
| **Mobile Element** | **5' palindromic end of element** | **3' palindromic end of element** | | **Comparison of 5' and 3' palindromic ends** |
| **HqIRS44 (405-406 bp)** | 5pr_fwd TCTAA ACGAAAAGCAGCGCGAGTTCCCCGCCCCACCGTGGGCGGGGGTGAA...  |||| ||||| ||||| ||||  5pr_rev ...TTCACCCCCGCCCACGGTGGGGCGGGGAACT... | 3pr_fwd ...GCGAAG--CCACGGGCCACCGCGCCCGTGGTACTTCAC  |||| |||||||| |||||||| ||||  3pr_rev GTGAAGTACCACGGGCGCGGTGGCCCGTGG--CTTCGC... | | 5pr_fwd TCTAA ACGAAAAGCAGCGCGAGTTCCCCGCCCCACCGTGGGCGGGGGTGAA...  || || |||||| | || ||  3pr_fwd ...GCGAAGCCACGGGCCACCGCGCCCGTGGTACTTCAC |
| **HqIRS46 (386-393 bp)** | 5pr_fwd TCTAC GCTACAGATTAACAGGCAGAGTGCCTCGGGGCTTGACCCCGAGGGTGAATGCCGTC...  |||| |||||||| |||||||| ||||  5pr_rev ...GACGGCATTCACCCTCGGGGTCAAGCCCCGAGGCACTCTGCCTGT... | 3pr_fwd ...CGGTGAAG--CCTCGGGGCTTGACCCCGAGGTACTTCAC  |||||| |||||||| |||||||| ||||||  3pr_rev GTGAAGTACCTCGGGGTCAAGCCCCGAGG--CTTCACCG... | | 5pr_fwd TCTAC AGATTAACAGGCAGAGTGCCTCGGGGCTTGACCCCGAGGGTGAATGCCGTCACT...  ||||||||||||||||||||||  3pr_fwd ...CGGTGAAGCCTCGGGGCTTGACCCCGAGGTACTTCAC  core del TCTAC AGATTAACAGGCAGAGTGCCTCGGGGCTTGACCCCGAGGTACTTCAC |
| **HqIRS54 (370-381 bp)** | 5pr_fwd TTAC GGCGGTGCGAGGAGAAAGCCCCGCCGTTCACGGCGGGATGAATC...  |||||||| ||||||||  5pr_rev ..GATTCATCCCGCCGTGAACGGCGGGGCTTTCT... | 3pr_fwd ...CGGCGCGGTGCCGTGGGAATCTTTGCCCTTCAGGGCGGAGGGGATGTCAA  || ||||| ||||| ||  3pr_rev TTGACATCCCCTCCGCCCTGAAGGGCAAAGATTCCCACGGCACCGCGCCG... | | 5pr_fwd .TTAC GGCGGTGCGAGGAGAAAGCCCCGCCGTTCACGGCGGGATGAATC...  . ||||||| | ||| ||| |||| |||||  3pr_fwd ...CGGCGCGGTGCCGTGGGAATCTTT-GCCCTTCAGGGCGGAGGGGATGTCAA |
| **HqIRS56 (532-574 bp)** | 5pr_fwd TTATA TGCTGAGCAGGCGCAATACCACGATCTTCAGGCCGTGGATACGCGCCGTCA...  ||||| ||||| ||||| |||||  5pr_rev ...TGACGGCGCGTATCCACGGCCTGAAGATCGTGGTATTGCGCCTGCT... | 3pr_fwd ...CCACCGCTCGGGATTCCTCCGCGTGAACCCGGAGGAGGATGTCAA  ||||||| || || |||||||  3pr_rev TTGACATCCTCCTCCGGGTTCACGCGGAGGAATCCCGAGCGGTGG... | | 5pr_fwd TTATA TGCTGAGCAGGCGCAATACCACGATCTTCAGGC-CGTGGATACGCGCCGTCACGC...  | | | || ||| | |||| | ||  3pr_fwd ...CCACCGCTCGGGATTCCTCCGCGTGAACCCGGAGGAGGATGTCAA |
| **HqIRS71 (543-590 bp)** | 5pr_fwd TTAG CTGAATACAGGCGCTAAGCTCCCTCCCTCAAGGAGCACCGCAGTC...  || ||||| ||||| ||  5pr_rev ...GGACTGCGGTGCTCCTTGAGGGAGGGAGCTTAGCGCCT... | 3pr_fwd ...AGCCCCGCCCTCAGCGAGGCCGTCAGGC--CGAGCACGGCGGGGTAGTTCAC  ||||||| | || ||| ||| || | |||||||  3pr_rev GTGAACTACCCCGCCGTGCTCG--GCCTGACGGCCTCGCTGAGGGCGGGGCT... | | 5pr_fwd TTAG CTGAATACAGGCGCTAAGCTCCCTCCCTCAAG--GAGCACCGCAGTC...  || || ||| || ||| | |||||| || |  3pr_fwd ...AGCCCCGCCCTCAGCGAGG-CCGTCAGGCCGAGCACGGCGGGGTAGTTCAC |
| **Section B: Relation to other elements** | | | | |
| **Mobile Element** | **5' End** | | **3' End** | |
| **HqIRS44 (405-406 bp)** | **HqIRS44 vs ISNph6 at 5pr end**  HqIRS44_5pr TCTAA ACGAAAAGCAGCGCGAGTTCCCCGCCCCACCGTGGGCGGGGGTGAAGCTC...  ||||| |||||||||||||||||||||||||||||| ||||||||||||||||| |  ISNph6_5pr TCTAA ACGAAAAGCAGCGCGAGTTCCCCGCCCCACTGTGGGCGGGGGTGAAGCGC... | | **HqIRS44 vs ISNph6 at 3pr end**  HqIRS44_3pr ...GAGACCACGGCGAAGCCACGGGCCACCGCGCCCGTGGTACTTCAC  |||||| |||||||||||||||||||||||||| | |||||  ISNph8_3pr ...TAATCCACGG-GAAGCCACGGGCCACCGCGCCCGTGGCAGTTCAC | |
| **HqIRS46 (386-393 bp)** | **HqIRS46 vs ISNph7 and ISHwa17 at 5pr end**  ISNph7_5pr TCTAC AGAGAAAGCAGGCAGAGTGCCTCGGGGCTTGACCCCGAGGGTGAATGCCGTAAGC  ||||| ||| || |||||||||||||||||||||||||||||||||||||||||||  HqIRS46_5pr TCTAC AGATTAA-CAGGCAGAGTGCCTCGGGGCTTGACCCCGAGGGTGAATGCCGTCACT  ||||| ||| || ||| |||||||||||||||||||||||||||| ||||||||||  ISHwa17_5pr TCTAC AGAGAAAGCAGCCAGAGTGCCTCGGGGCTTGACCCCGAGGCTGAATGCCGTAAGC | | **HqIRS46 vs ISNph12 at 3pr end**  HqIRS46_3pr ...CGGTGAAGCCTCGGGG------------------------------------------------------CTTGACCCCGAGGTACTTCAC  |||||||||||| ||||||||||||| |||||  ISNph12_3pr ...ACGGGAAGCCTCGGGGTCGTACGGAAGACGGAGTCTTCCGTGATGACGAGAGACGAAGTCCCTCGAACCACTTGACCCCGAGGCGGTTCAC | |
| **HqIRS54 (370-381 bp)** | **HqIRS54 vs ISNph18 and ISHwa24 at 5pr end**  ISNph18_5pr TTAC AGCGGTAGCAAGGCGAAAGCCCACCCGTTTACGGGTGTGGATGAAGC...  |||| ||||| || ||| |||||||| ||||| |||| ||||||| |  HqIRS54_5pr TTAC GGCGGT-GCGAGGAGAAAGCCCCGCCGTTCACGG--CGGGATGAATC...  |||| || | ||||||||||||| ||| |||| || ||||||||||  ISHwa24_5pr TTAC GGAGAG-CCGAGGAGAAAGCCTCGCGCTTCAGCG-CTGGGATGAATC... | | **HqIRS54 vs ISNph18 and ISHwa24 at 3pr end**  ISNph18_3pr ...ACCGTAAACCCTAATATCCCAA-CGTGGGAATCCCACGGCTTCAGCCGTGTGGAGGATGTCAA  | | || || | |||||||||| |||||| | || |||||||||  HqIRS54_3pr ...ATCCCAATCCAGCGGCGCGGTGCCGTGGGAATCTTTGCCCTTCAGGGCGGAGG-GGATGTCAA  ||||||| |||||| ||||||||||||||||| | || |||||| |||| | |||||||||  ISHwa24_3pr ...ATCCCAACTCAGCGGTGCGGTGCCGTGGGAATCCTCGCGCTTCAGCGCGGGGA-GGATGTCAA | |
| **HqIRS56 (532-574 bp)** | **HqIRS56 vs ISHwa21 at 3pr end**  HqIRS56_3pr ...CCACCGCTCGGGATTCCTCCGCGTGAACCCGGAGGAGGATGTCAA  | | ||||||||||||||||||| ||||||||||||||||  ISHwa21_3pr ...CGGTGCCGTGGGATTCCTCCGCGTGAACGCGGAGGAGGATGTCAA | |  | |
| **HqIRS71 (543-590 bp)** | **HqIRS71 vs ISNph20 at 5pr end**  HqIRS71_5pr TTAG CTGAATACAGGCGCTAAGCTCCCTCCCTCAAGGAGCACCGCAGTCCGCGCG...  |||| |||||| |||||| ||||| |||| |||||||||||| ||||| |||  ISNph20_5pr TTAG CTGAATGCAGGCGGTAAGCCCCCTTCCTCAAGGAGCAACGCAGGCCGAAGG... | | **HqIRS71 vs ISNph20 at 3pr end**  HqIRS71_3pr ...ACAACGTCGTAGAAGCAGGAAGCCCCGCCCTCAGCGAGGCCGTCAGGCCGAGCACGGCGGGGTAGTTCAC  |||||| ||||||||||||||| ||||||||||| |||||| |||||| || ||||||||||||  ISNph20_3pr ...AGCGCGTCGTGGAAGCAGGAAGCCCCACCCTCAGCGAGCGCGTCAGCGCGAGCAGGGTGGGGTAGTTCAC | |
