# Supplementary material for: Haloquadratum walsbyi : Limited Diversity in a Global Pond
Source: PLoS One. 2011 Jun 20;6(6):e20968. doi: 10.1371/journal.pone.0020968 (PMC3119063; doi:10.1371/journal.pone.0020968)
Supplement: Text S1 — Descriptions of genomic changes related to MGEs. (DOC) [file pone.0020968.s011.doc]

## Text S1. Descriptions of genomic changes related to MGEs.

## a) A potential ‘outbreak’ of four transposons from DV10

A 43 kb C23^T^-specific sequence (DV10, #578 in Additional Table 5) is a region that carries a total of 10 transposons or transposon-pseudogenes, several glycosyltransferase genes and one phage integrase homolog. This sequence probably represents an integration of foreign DNA. This region may be responsible for the infection of strain C23^T^ with four transposons (ISHwa4, ISHwa8, ISHwa12, and ISHwa13).

Strain C23^T^ has 10 copies of ISHwa4 of which one is on the 43 kb sequence DV10 while the other 9 are separate strain-specific insertion events (SSEIs) elsewhere in the genome. ISHwa4 is the most active transposon in C23^T^ and is restricted to this strain.

Similar ‘outbreaks’ of ISH elements from DV10 may have occurred for ISHwa8 (four SSEIs), ISHwa12 (four SSEIs), and ISHwa13 (two SSEIs). In all cases, SSEIs are restricted to C23^T^. Thus, four of the seven transposon families which show SSEIs only in C23 ^T^ may have entered the cell in a single infection event.

ISHwa13 is carried as complete transposon on DV10, while ISHwa12 has suffered an internal 42 bp deletion, which includes one copy of a 9 bp direct repeat and removes 14 residues from the transposase gene. Also, the ISHwa8 copy on DV10 has been inactivated by targeting with the small mobile repeat HqIRS40. It has to be assumed that inactivation of the supposed original copies of ISHwa12 and ISHwa8 occurred after the elements had spread to other genome locations.

## b) Mobilization of a transposon with subsequent removal of the original copy: case 1

ISHwa1 occurs only once as a complete transposon in each strain, but in different relative positions. The elements differ by only 3 bases at the nucleotide level. In HBSQ001, ISHwa1 is an SSEI while it occurs within a deletion-coupled insertion region in C23^T^ (DV9, #480 in Additional Table 5). Tetra-nucleotide analysis indicates that C23^T^ has the original sequence. This infers that the deletion-coupled insertion event occurred in HBSQ001, removing the ISHwa1 copy corresponding to that of C23^T^. The remaining copy in HBSQ001 would then represent an earlier mobilization event from the original ISHwa1 that predated the replacement. While there is another ISHwa1 element in HBSQ001, this is probably not the ancestor. The sequence is much more different (25 mutations) than the transposon from the other strain (only 3 mutations). In addition, the transposase is not functional as it has an in-frame stop codon.

## c) Mobilization of a transposon with subsequent removal of the original copy: case 2

Strain C23^T^ has one complete copy of IS605-type transposon ISHwa23 while strain HBSQ001 has two copies. In each strain, one of the copies is an SSEI. The other copy of HBSQ001, a 1500 bp sequence which codes for an IS1341-type transposase (TnpB), occurs at the same genome position as a 52 bp PATE in C23^T^. This PATE represents a terminal-only copy of the element with a core deletion that has occurred at a 19 bp direct repeat which contains the near-terminal palindrome (Additional Table 6).

This configuration results in a long (1500 bp) stretch of near-identical sequence at distinct genome positions, representing a candidate for a genome rearrangement. However, it has to be concluded that the configuration is due to transposition of the element by mobilization with subsequent removal of the original copy, in this case by repeat-mediated core deletion within the transposon.

## d) Mobilization of a MITE and a transposon despite lack of a functional suitable transposase

Mobilisation of MITE HqIRS39 would be expected to have occurred by the transposase of the parent transposon HqIRS12, but both strains contain exclusively pseudogenes of the HqIRS12 transposase, due to (multiple) frame-shifts or in-frame stop codons. Either, the transposases have been inactivated only after HqIRS39 mobilization or HqIRS39 can be mobilized by another transposase, possibly that of ISHwa6 which is homologous to the HqIRS12 transposase. However, the ITRs of HqIRS12 and ISHwa6 show only limited similarity and the transposases show only 36% aa identity. A related case occurs for another homolog of the ISHwa6 transposase, the HqIRS11 transposase for which only pseudogenes are present. HqIRS11 is found only in C23^T^ in the form of two SSEIs.

## e) Switch_repeat: an MGE is targeted at the same position by either distinct elements or the same element in opposite orientation

In five cases, an equivalent pair of PATEs in the two *Haloquadratum* strains may have been targeted independently at an identical position. This is designated as “switch_repeats” in Additional Table 5.

In four cases, the same type of element has inserted in opposite orientation. This occurred for each of the small mobile repeats HqIRS42 and HqIRS43 as well as for MITE HqIRS35 (2 cases). In the 5^th^ case, the element is targeted by HqIRS43 in C23^T^ while it is targeted by HqIRS42 in HBSQ001.
